# A global assessment of the vulnerability of shellfish aquaculture to climate change and ocean acidification

**DOI:** 10.1002/ece3.6149

**Published:** 2020-03-12

**Authors:** Phoebe J. Stewart‐Sinclair, Kim S. Last, Ben L. Payne, Thomas A. Wilding

**Affiliations:** ^1^ The Scottish Association for Marine Science Scottish Marine Institute Oban UK; ^2^ Natural England York UK

**Keywords:** aquaculture, climate change, food‐security, ocean acidification, shellfish, vulnerability assessment

## Abstract

Human‐induced climate change and ocean acidification (CC‐OA) is changing the physical and biological processes occurring within the marine environment, with poorly understood implications for marine life. Within the aquaculture sector, molluskan culture is a relatively benign method of producing a high‐quality, healthy, and sustainable protein source for the expanding human population. We modeled the vulnerability of global bivalve mariculture to impacts of CC‐OA over the period 2020–2100, under RCP8.5. Vulnerability, assessed at the national level, was dependent on CC‐OA‐related exposure, taxon‐specific sensitivity and adaptive capacity in the sector. Exposure risk increased over time from 2020 to 2100, with ten nations predicted to experience very high exposure to CC‐OA in at least one decade during the period 2020–2100. Predicted high sensitivity in developing countries resulted, primarily, from the cultivation of species that have a narrow habitat tolerance, while in some European nations (France, Ireland, Italy, Portugal, and Spain) high sensitivity was attributable to the relatively high economic value of the shellfish production sector. Predicted adaptive capacity was low in developing countries primarily due to governance issues, while in some developed countries (Denmark, Germany, Iceland, Netherlands, Sweden, and the United Kingdom) it was linked to limited species diversity in the sector. Developing and least developed nations (*n* = 15) were predicted to have the highest overall vulnerability. Across all nations, 2060 was identified as a tipping point where predicted CC‐OA will be associated with the greatest challenge to shellfish production. However, rapid declines in mollusk production are predicted to occur in the next decade for some nations, notably North Korea. Shellfish culture offers human society a low‐impact source of sustainable protein. This research highlights, on a global scale, the likely extent and nature of the CC‐OA‐related threat to shellfish culture and this sector enabling early‐stage adaption and mitigation.

## Graphical abstract

Bivalve culture has enormous potential to deliver protein for an expanding population, but bivalves are vulnerable to climate change and ocean acidification (CC‐OA). We modeled the future (2020–2100) vulnerability of global bivalve culture to CC‐OA and conclude that the greatest challenge will occur from 2060 onwards. In Asia, the threat from decreased primary productivity will be challenging to address and we recommend mitigation through diversification in the European sector.

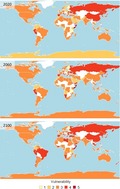

## 1 INTRODUCTION

Climate change and ocean acidification (CC‐OA), caused by mankind's release of carbon dioxide and other greenhouse gases, poses a major threat to current and future human well‐being (Doney, Fabry, Feely, & Kleypas, 2009; Houghton et al., 2001). Securing food, particularly protein, for the growing human population is a major global concern, considering the current fully or overexploited status of many wild food stocks and limited supply of freshwater for terrestrial protein production (Alleway et al., 2019; Callaway et al., 2012). Outside of food provisioning, the ecosystem services generated by bivalve aquaculture have been valued at ~$6.5 billion per year (van der Schatte Olivier et al., 2018).

Mariculture, the farming of food organisms in the sea is an increasingly important source of animal protein that is independent of freshwater and which usually occurs within a complex and space‐competitive coastal margin (Callaway et al., 2012). Mariculture operations generate income and employment in coastal communities, and mollusk‐culture operations are considered to have minor environmental impact compared to other forms of farming (De Silva & Soto, 2009; Mohanty, Sharma, Sahoo, & Mohanty, 2010; Wilding & Nickell, 2013). “Conservation aquaculture,” based around bivalves, can offer relief for wild‐caught fisheries (Froehlich, Gentry, & Halpern, 2017), while simultaneously increasing water quality and providing habitats for other species.

Noncephalopod mollusks (termed “mollusks” here), and the focus of the current paper, are cultured both inter‐ and subtidally in every populous continent and are of considerable economic value (>US$17 billion per annum in 2015; FAO, 2016). Molluskan aquaculture offers considerable scope for expansion including into areas damaged by climate change‐linked sea water intrusion or coastal flooding (De Silva & Soto, 2009). Mollusks are cultured at a range of scales and constitute a staple protein or a niche, luxury food (e.g., Europe; De Silva & Soto, 2009). Mollusk mariculture growth potential extends to nations not currently culturing mollusks (De Silva & Soto, 2009).

Climate change and ocean acidification will have direct consequences on molluskan mariculture operations and also a range of indirect effects via changing patterns of precipitation, salinity, the frequency and severity of extreme weather events (Brugère & De Young, 2015; Frost et al., 2012), changes in primary productivity, the nature/frequency of harmful algal blooms, and the incidence/spread of disease and invasive species (Karvonen, Rintamäki, Jokela, & Valtonen, 2010). CC‐OA will threaten the viability of molluskan mariculture at local and national spatial scales which depend primarily on the geographical location of the farms, the species being cultivated, the hosting nation's ability to adapt to CC‐OA, developments in market and trading patterns, and the emergence of new culture practices and technologies (Brugère & De Young, 2015).

Mollusks have been identified as a group particularly vulnerable to the combined effects of CC‐OA (Hughes et al., 2012). Vulnerability assessments are routinely applied to fishery stocks and fish‐aquaculture operations, from both environmental and socioeconomic perspectives (reviewed in Barsley, De, Young, & Brugère, 2013). While the vulnerability of aquaculture more broadly has been recently investigated (Handisyde, Telfer, & Ross, 2017), the specific vulnerabilities of shellfish mariculture to CC‐OA are currently poorly understood and national‐level predictions are urgently needed to identify at‐risk populations and economies (Allison, Perry, et al., 2009). Identification of nations with high sensitivity and low adaptive capacity to future CC‐OA changes is crucial for future decision making and policy development. Froehlich, Gentry, and Halpern (2018) quantified the effect of climate change on the future production potential of finfish and bivalves, while Gentry et al. (2017) mapped the potential for global development of marine aquaculture. However, these studies did not incorporate socio‐political factors such as governance, and the economic and nutritional importance of mariculture at a national level (Froehlich et al., 2018; Gentry et al., 2017). For nations with the highest nutritional or economic dependence on mariculture, declines in production due to CC‐OA or other threats, will be disproportionately felt (Froehlich et al., 2018). In Froehlich et al. (2018), while the species‐specific growth limits are used to map where cultivation could occur, the current diversity and consequent resilience of the national mariculture industry is not taken into account. This is an important factor to capture, as the diversity of a system, natural or anthropogenic, confers adaptive capacity to threats related to CC‐OA.

Our objective was to conduct a vulnerability assessment of shellfish production on a global scale and to highlight where and when the global mollusk mariculture sector will be at highest risk (tipping points). In order to achieve this objective, we predicted the vulnerability of global mollusk mariculture, over the period 2010–2100, to the effects of CC‐OA, by deriving then combining, at a national level, indices of exposure, sensitivity, and adaptive capacity.

## 2 METHODS

**Figure 1:**
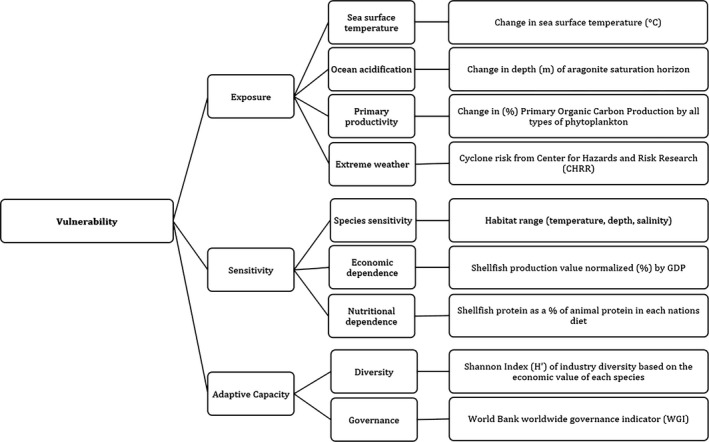
Schematic representation of the vulnerability assessment model illustrating that overall vulnerability was determined as a combination of three exposure, sensitivity, and adaptive capacity each of which consisted of subfactors. Details for each factor/subfactor are given in the methods

The background and context to the model are described; then, we detail the model construction. The model structure and components are summarized in Figure 1.

### 2.1 Model rationale

This vulnerability assessment was applied at a national level since this is the level at which changes in policy and infrastructure can best be initiated (Brugère & De Young, 2015). Antarctica was considered a “nation” for the purposes of this analysis. All coastal nations were assessed, including those where there is no Food and Agriculture Organization (FAO) record of mollusk mariculture. As most mollusk aquaculture production (97.5% in 2014) takes place in the marine environment (FAO, 2016), this analysis was limited to marine mollusk aquaculture.

Vulnerability is defined by the Intergovernmental Panel on Climate Change (IPCC, 2007) as the degree to which a system is susceptible to adverse effects. In this study, we use the approach outlined by the IPCC to build a vulnerability assessment using indicators to build sublayers of exposure, sensitivity, and adaptive capacity in the mollusk mariculture industry (Adger, 2006; IPCC, 2007; Figure 1).

The exposure sublayer is a measure of the predicted physical effects of CC‐OA on the mollusk mariculture industry including their direct effects (elevated sea surface temperatures and decreased pH) and indirect effects (primary productivity, and risk of extreme weather events; Adger, 2006). Exposure to predicted sea surface temperatures (SST) outside of a species' thermal tolerance and a reduction in aragonite saturation (AΩ) are both detrimental to mollusks (Cooley, Lucey, Kite‐Powell, & Doney, 2012; Pörtner & Farrell, 2008). OA was represented by the predicted change in depth (m) of the aragonite saturation horizon, since availability of suitable habitat will decrease as the saturation horizon becomes shallower.

Food satiation provides a buffer against the detrimental effects of OA (Melzner et al., 2011) and, since maricultured mollusks are primarily filter feeders consuming phytoplankton (Winter, 1978), predicted ocean primary productivity (PP) was used as an indicator for food availability. It was assumed that predicted decreases in primary productivity would render mollusks proportionally less able to buffer the effects of OA (Burgiel & Muir, 2010; Dupont & Thorndyke, 2009; Parker et al., 2012). Extreme weather events have the potential to cause substantial mariculture‐infrastructure damage. Predicting future extreme weather events is challenging (Nehls & Thiel, 1993) and, consequently, we used the approach adopted by Handisyde et al., (2006), which used current‐day cyclone risk from CCCMA (2016) as an indicator for future extreme weather risk. Other relevant variables, such as occurrence of harmful algal blooms, disease, and pest species, were not included in this study due to paucity of data availability at the spatial and temporal scale considered here.

Climate change and ocean acidification projections (sea surface temperature, aragonite saturation, and primary productivity) were derived from the IPCC's Representative Concentration Pathways 8.5 (RCP8.5) “business as usual” model (AR5; Moss et al., 2010; Stocker, 2014). RCP8.5 assumes the highest greenhouse gas emissions and, consequently, the greatest degree of CC‐OA (Riahi et al., 2011; van Vuuren et al., 2011).

The sublayer sensitivity (Figure 1) is defined by the IPCC as the intrinsic degree to which biophysical, social, and economic conditions are likely to be influenced by extrinsic stresses or hazards (Houghton et al., 2001; IPCC, 2007). Here, we measure sensitivity using indicators for species sensitivity and the relative economic and nutritional contribution made by mollusks. Different species have different inherent capacity to adapt to environmental changes, and species with a wider habitat range are likely to be less sensitive to those changes (Pörtner & Farrell, 2008). Our species sensitivity index assumes that species characterized by broad environmental tolerances will be more robust against future environmental change (Morrison et al., 2015). The economic and nutritional contribution index assumed that nations with a higher proportion of their aquaculture sector invested in mollusk mariculture and with a larger per‐capita consumption of mollusks are more likely to be sensitive to CC‐OA‐related fluctuations in mariculture industry production (Allison, Perry, et al., 2009; Handisyde, Ross, Badjeck, & Allison, 2006).

The sublayer adaptive capacity includes social, economic, technological, biophysical, and political indicators that determine the capacity of systems to adapt to change (IPCC, 2007). CC‐OA represents a significant test to the stability and adaptive capacity of the mariculture sector, and factors such as socioeconomic status have not been incorporated in prior studies (Froehlich et al., 2018). Adaptive capacity was measured here using indicators for industry diversity (number of species and quantity per species) and governance (national ability to manage the necessary investment into mariculture to overcome CC‐OA; Handisyde et al., 2006).

The impacts of CC‐OA on mariculture will change over time; identification of tipping points, where CC‐OA could trigger sharp declines in industry production, is important to identify appropriate timelines for mitigation/management plans (Cai, Judd, Lenton, Lontzek, & Narita, 2015; Lenton, 2013). Further details in relation to model parameterization and the derivation of indices are provided in following subsections.

### 2.2 Model construction

The vulnerability assessment was based on overlaying spatially explicit CC‐OA projections and national mollusk production statistics/metrics and socioeconomic data. All spatial data were standardized onto the World Geodetic System 1984 (WGS84), using R version 3.3.2 (R Core Team, 2013).

All indices generated within the sublayers exposure, sensitivity, and adaptive capacity (Figure 1) were applied to United Nations recognized nations (and Antarctica, see caveat above), and these were assigned World Bank development classifications (Prince & Fantom, 2014). The numerical values generated for all indices were reclassified to an impact scale of one to five (Handisyde et al., 2006), with five indicating the greatest impact. Since the data used here were varied in type, scale, and composition, it was not possible to apply a standard method of reclassification to all indices. Reclassification of each variable was made on a case‐by‐case basis as indicated by relevant literature (see below). For variables where there was no reclassification precedent, value‐reclassification followed Allison, Perry, et al. (2009); all values were normalized onto a scale from 0 to 1 $\left(X^{\prime }=\frac{X-X_{\min}}{X_{\max}-X_{\min}}\right)$and linearly reclassified to our 1–5 risk score. Missing data for indices within each sublayer were not included and an average value calculated for each nation. The overall score for each sublayer was assigned using the mean value from indices within each sublayer (0–1 = very low, 1–2 = low, 2–3 = moderate, 3–4 = high, and 4–5 = very high).

To test for redundant indices within each sublayer of our vulnerability model, we conducted principal component analysis (PCA; Wold, Esbensen, & Geladi, 1987) following data normalization. To facilitate global analysis, all indicators were necessarily coarse‐scale, and we acknowledge that this reduces accuracy and resolution of results at finer scales.

#### 2.2.1 Exposure sublayer

The exposure sublayer was generated by combining indicators for sea surface temperature (SST), OA (as indicated by aragonite saturation horizon; AΩ), primary productivity (PP), and risk of extreme weather events (Figure 1). Forecasted data for the exposure submodel (period 2006–2100) were obtained from the CMIP5 archive (see Data availability statement, 1) based on the “business as usual” RCP8.5 scenario (Moss et al., 2010). We used the RCP8.5 scenario exclusively in this analysis, as other scenarios include future changes in socioeconomic status of nations not modeled here. As such, it is appropriate that the scenario used is in line with the static nature of the indicators in the sensitivity and adaptive capacity sublayers. In order to set a baseline for each index, the mean values from historical data for the period 1860–2005 were calculated (Henson, Beaulieu, & Lampitt, 2016). The projected data were projected on to a latitude–longitude grid with a 1° and 1/3° resolution between 90° to 30° and 30° to zero degree latitude, respectively. Our model was based on RCP8.5 water column parameter projections from surface to 100 m resolved to 10 m in the CMIP5 data (Collins et al., 2008).

Sea surface temperature, depth of the aragonite saturation horizon, and primary productivity (primary organic carbon production combined across all phytoplankton) were averaged for 120‐monthly values as predicted for each decade between 2011–2020 and 2091–2100 (Cooley et al., 2012). This average was then expressed as % change (for aragonite saturation horizon and primary productivity) or change in °C (for SST), with respect to the baseline mean for 1986–2005 (Henson et al., 2016). Changes in SST (°C), saturation depth, and primary productivity were reclassified to represent higher levels of exposure to CC‐OA as in Allison, Perry, et al. (2009; Table 1) (see Tables S1–S3 for temperature, aragonite saturation, and productivity, respectively).

**Table 1:** Reclassification of data layers used in the exposure to CC‐OA submodel, where 5 indicates the highest exposure to CC‐OA for the year 2100

Exposure	Sea surface temperature (°C) increase	Aragonite saturation depth (%) decline	Primary productivity (%) decline	CHHR cyclone index
1	0–2	<0	<0	1–2
2	2–3	0–25	0–25	3–4
3	3–4	25–50	25–50	5–6
4	4–5	50–75	50–75	7–8
5	5	75–100	75–100	9–10

Note

CHHR—Center for Hazards and Risk Research (see main text for additional explanation).

The index for extreme weather event submodel used data from the Center for Hazards and Risk Research (CHRR) at Columbia University (see Data availability statement, 2), based on global 2.5° grid of 1,600 cyclone events over the period 1980–2000 (CCCMA, 2016). In the absence of relevant, usable forecasted data, it was assumed that the number/intensity of extreme events was most likely to increase in areas that currently experience high frequencies of these events (Handisyde et al., 2006). Layers were linearly reclassified from the CHRR risk deciles to our impact scale of 1–5 (Table 1, Table S4).

All spatial data predictions for indices within the exposure submodel (sea surface temperature, aragonite saturation horizon, primary productivity, and cyclone risk) were reprojected on to a standard WGS84 grid and assigned to nations using national boundary data from the Flanders Marine Institute Maritime Boundaries Geodatabase (see Data availability statement, 3) (Cooley et al., 2012; VLIZ, 2012). As in Froehlich et al. (2018), we assume that nations will not establish mariculture operations outside of their jurisdiction, and so we restrict our estimates for exposure to the areas bounded by each nation's exclusive economic zone (EEZ). Mean exposure submodel predictions were determined for each time period (2020–2100). The exposure values for discontinuous EEZ's (i.e., those nations with overseas territories) were collated prior to the average being determined.

#### 2.2.2 Sensitivity sublayer

The sensitivity sublayer of the mollusk mariculture industry to CC‐OA was quantified by collating cultured‐species sensitivity and economic/nutritional contribution indicators (Figure 1).

Species sensitivity was determined by the thermal, depth, and salinity tolerances of each species based on their distribution (see Data availability statement, 4) (Ocean Biogeographic Information System; Grassle, 2000). A species sensitivity score was calculated for each species for each of the temperature, depth, and salinity variables (Table 2, Table S6 for species detail and Table S7 for national species sensitivity scores). The arithmetic mean of the sensitivity scores for all species was calculated for each nation, providing an average overall species sensitivity score for the mollusk mariculture industry. Species with greater habitat range were assigned a lower sensitivity score (Table 2). In the absence of species‐specific data, data from congenerics were used and, where this was absent, the species was excluded from this analysis.

**Table 2:** Reclassification of data layers used in the species sensitivity index within the sensitivity sublayer, where 5 indicates the highest sensitivity to CC‐OA

Sensitivity score	Species' distribution		
	Depth range (m)	Temperature range (°C)	Salinity range (PSS)
1	>100	> 20	> 20
2	75–100	15–20	15–20
3	50–75	10–15	10–15
4	25–50	5–10	5–10
5	0–25	0–5	0–5

Note

An unweighted average of the scores for each layer (depth, temperature, and salinity) determined the overall species sensitivity for each species cultured within each nation.

The economic contribution index was calculated as the total molluskan mariculture production value (US$; FAO, 2016) expressed as a percent of GDP (see Data availability statements 5 and 6, respectively) (World Bank Development Indicators; Prince & Fantom, 2014) as directed in Allison, Perry, et al. (2009) and Cooley et al. (2012). For each nation, the mean mollusk production value from 2012 to 2014 was determined (Handisyde et al., 2006) as a percent of GDP (Allison, Perry, et al., 2009). The nutritional contribution index was, for each nation, represented by the percentage of dietary protein contributed by mollusks (Allison, Perry, et al., 2009; Cooley et al., 2012) as indicated on FAO food balance sheets (FAO, 2016). The economic and dietary contributions were then normalized and linearly reclassified to our 1–5 impact scale (detail given above; Allison, Perry, et al., 2009).

#### 2.2.3 Adaptive capacity sublayer

The adaptive capacity sublayer for the mollusk mariculture industry was calculated for each nation and was based on a combination of current industry diversity (i.e., number of species cultivated, see Data availability statement, 7) (US$; FAO, 2016) and national governance indices (Figure 1 and see below). For diversity, we determined the Shannon Index, H' (base = natural logarithm) (Shannon, 2001), which balances the richness (species count) and evenness (production tonnage per species; FAO, 2016). The diversity index value for each nation was normalized (see “Model construction”) and inverted (1 = high diversity, 5 = low diversity; Table S12).

The governance index was defined by the World Bank Worldwide Governance Indicator (WGI), which combines six dimensions of governance (Cooley et al., 2012; Kaufmann, Kraay, & Mastruzzi, 2011). The WGI used here see (Data availability statement, 8) was the average determined over the period 1996–2015 equally weighted for the six components and divided into 5 equal classes and inverted to reclassify on to our 1–5 scale.

#### 2.2.4 Overall Vulnerability

Overall vulnerability, per nation, was determined by combining the unweighted average score for sublayers exposure, sensitivity, and adaptive capacity (Allison, Perry, et al., 2009). The sublayers of vulnerability have been given various objective‐dependent weightings in prior studies (Brugère & De Young, 2015). Some studies have sourced expert elicitation (Handisyde et al., 2006; Morrison et al., 2015) while others based weighting on scientific evidence supporting their relative importance (Cinner et al., 2013), or have given equal weighting to all components (Allison, Beveridge, & van Brakel, 2009). Given this, and the absence of an intuitive relationship between the three components in our model, we opted to leave sublayers unweighted. As the robustness of vulnerability scores to additive and multiplicative approaches is highly correlated (Allison, Perry, et al., 2009), we opted for an additive model where the unweighted mean score of the sublayers (exposure, sensitivity, and adaptive capacity) was combined to give an overall vulnerability score (0–1 = very low, 1–2 = low, 2–3 = moderate, 3–4 = high, and 4–5 = very high). Missing data were omitted in the determination of the mean (see Table S14 for overall vulnerability over time, by nation).

##### Tipping points

Nonmetric multidimensional scaling (MDS; Kenkel & Orlóci, 1986) was performed on the overall vulnerability score for each nation for the decades 2011–2020 to 2090–2100 to identify temporal trends in predicted vulnerability and tipping points. MDS creates an ordination that displays the differences between objects (here: decades), where objects with greater difference are displayed further apart. For each of the top 15 mollusk producers, MDS axis scores were correlated with exposure scores for indices within the exposure sublayers to assess which sublayer was driving any observed patterns. Environmental indices were added to MDS plots to demonstrate potential drivers of tipping points. Cyclone risk was excluded from the MDS‐based analysis as it was calculated from historic observations and did not vary over time. For correlations between MDS axis scores and indices within the exposure sublayer, for the top 15 mollusk mariculture‐producing nations (Table S15).

## 3 RESULTS

Indices for exposure, sensitivity, and adaptive capacity were determined for 137, 142, and 208 nations, respectively, and were combined to calculate overall vulnerability for 117 nations. Differences in the number of indices generated between the different submodel components occurred because not all indices were applicable to all nations (e.g., not all nations currently culture mollusks) and because of data availability.

### 3.1 Exposure

Exposure was calculated for sea surface temperature (SST), aragonite saturation horizon (AΩ), primary productivity (PP), and risk of extreme weather events, for 52,646 locations in 137 maritime nations, for each decade from 2020 to 2100. Within the exposure sublayer, PCA of the initial four indices found no redundant indices. The strongest correlation (*r* = −.39) was between SST and AΩ.

**Figure 2:**
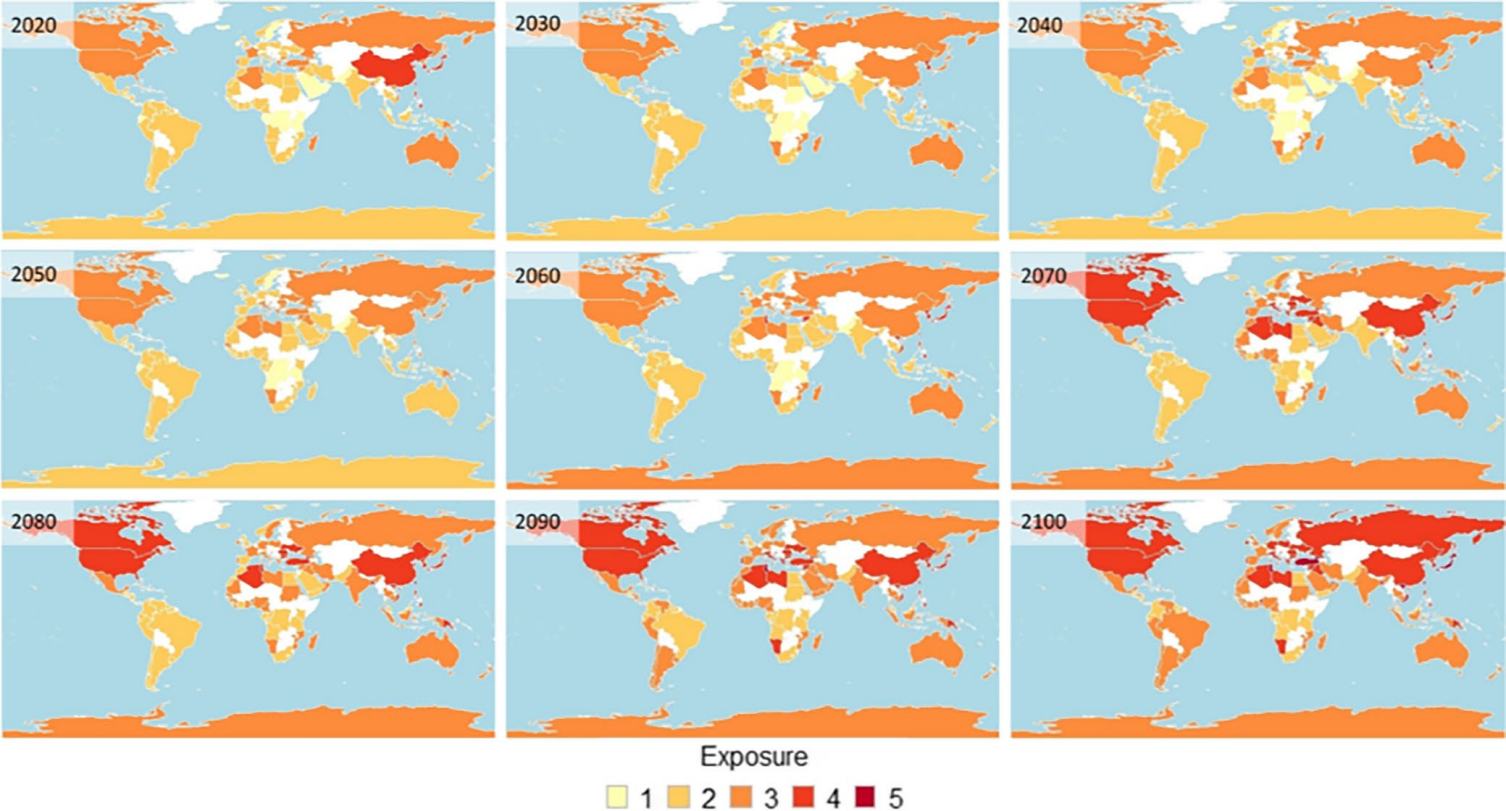
Exposure to CC‐OA for nations with current mollusk aquaculture operations, measured as nation‐specific mean of indices within the exposure sublayer from 2020 to 2100, for IPCC scenario RCP8.5. Colors represent exposure score (1 = very low, 2 = low, 3 = moderate, 4 = high, and 5 = very high), with white where no data were available. See Table S5 for national values

Exposure risk increased over time from 2020 to 2100, with ten nations predicted to experience very high exposure to CC‐OA in at least one decade during the period from 2020 to 2100 (Figure 2).

Nations that scored high exposure for changes in SST also tended to score high for changes in AΩ (Table 3). Georgia and neighboring Turkey, along with Tunisia, scored very high exposure for both SST (>5 C increase) and AΩ (>75% shallower; Table 3). Japan, Taiwan, and Vietnam were also categorized into the highest exposure risk, but this was primarily attributable to a combination of predicted declining primary productivity (>75% by 2100) and high cyclone risk.

**Table 3:** Exposure risk values for nations with very high overall exposure to CC‐OA in the decade ending 2100, showing indices that make up the exposure sublayer

Country	Sea surface temperature (°C)	Aragonite saturation depth	Primary productivity	Cyclone risk	Exposure 2100
Developed					
Japan	4	4	4	**5**	5
Developing					
Bahamas*				**5**	5
Taiwan	3	4	**5**	**5**	5
Vietnam*	3		**5**	**5**	5
Georgia*	**5**	**5**	3		5
Tunisia*	**5**	**5**	3		5
Turkey*	**5**	**5**	3		5

Note

Values in bold indicate very high exposure to an index within any layer. Blank cells indicate missing data. The overall score for exposure was assigned using the mean value from indices within the layer and categorized thus: 0–1 = very low, 1–2 = low, 2–3 = moderate, 3–4 = high, and 4–5 = very high. Nations missing data for any single index have been identified with an asterisk (*).

Eight of the 15 top mollusk mariculture‐producing nations scored high or very high in terms of overall exposure (Table 4). Six of the 15 top‐producing nations are in Asia (China, Japan, South Korea, Vietnam, Taiwan, and North Korea), with overall high exposure primarily attributable to high cyclone risk (Table 4). Outside of Asia, the United States and Italy were the only top‐producing nations with predicted high exposure (attributable to SST/cyclone and SST/aragonite depth, respectively; Table 4).

**Table 4:** Predicted exposure to CC‐OA for 15 largest producers of mollusk mariculture in 2014 (FAO, 2016), for the year 2100 including indices sea surface temperature (SST), aragonite saturation horizon, primary productivity, and cyclone risk

Nation	Sea surface temperature (°C)	Aragonite saturation depth	Primary productivity	Cyclone risk	Exposure 2100
**China**	3	4	3	**5**	4
**Japan**	4	4	4	**5**	**5**
**South Korea**	3	**5**	3	**5**	4
Chile*	2	3	2		3
Spain	4	2	2	1	3
Thailand	3	1	3	2	3
**Vietnam**	3		**5**	**5**	**5**
USA	4	3	3	4	4
France	3	3	2	4	3
Italy*	**5**	4	2		4
New Zealand	3	4	2	2	3
**Taiwan**	3	4	**5**	**5**	**5**
**North Korea**	4	**5**	2	4	4
Netherlands	4	1	2	2	3
Peru*	3	2	2		3

Note

For other table details (highlights, asterisk, missing values, and scoring), see Table 3 caption.

### 3.2 Sensitivity

Sensitivity was calculated for 144 nations. Indices for species sensitivity, nutritional contribution, and economic sensitivity were calculated for 55, 124, and 136 nations, respectively (see Tables S7–S9 for details). PCA of the initial four indices showed that the first three principal components contributed 98.5% to the total variation; a correlation of 0.88 was observed between the mollusk economic value and the production index so the production index was dropped.

**Figure 3:**
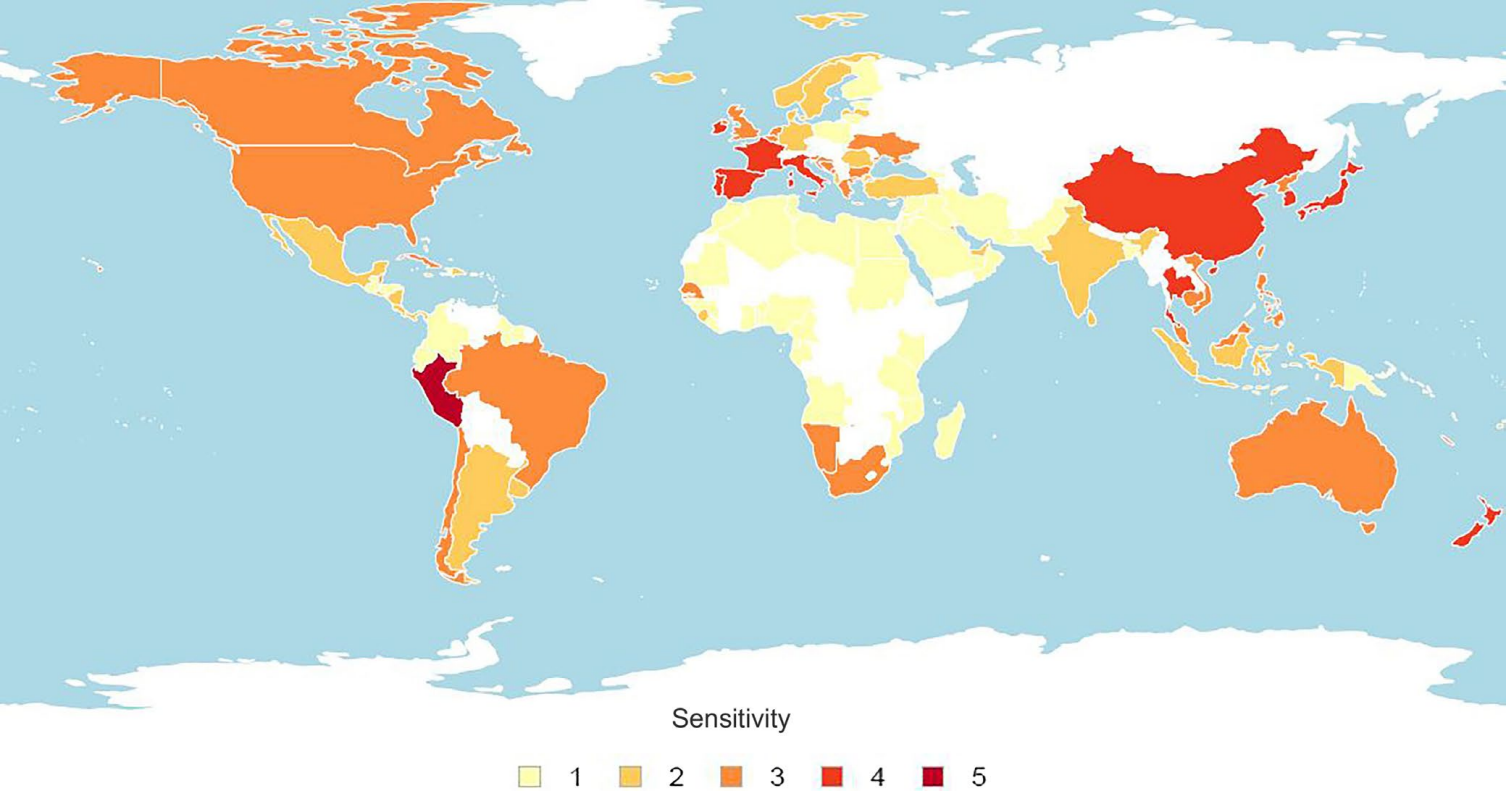
Sensitivity to CC‐OA for nations with current mollusk aquaculture operations, measured as nation‐specific mean of indices within the sensitivity sublayer. Colors represent sensitivity score (1 = very low, 2 = low, 3 = moderate, 4 = high, and 5 = very high). White areas indicate nations where no data were available

Nations predicted with the very high sensitivity score were Peru, Antigua and Barbuda, and the Cook Islands. This very high sensitivity was primarily attributed to the narrow habitat range of species cultured, for example, the Cook Islands' and Peru's mollusk mariculture sectors are dominated by the clam *Tridacna* spp. and Peruvian calico scallop (*Argopecten purpuratus*), respectively (FAO, 2016), which are both habitat specialists (Grassle, 2000). Five of 11 nations with high sensitivity were in Europe (France, Ireland, Italy, Portugal, and Spain) primarily driven by the mariculture sector's economic value (Figure 3; Table 5). Conversely, high sensitivity in Asia (China, Japan, and Thailand) was attributed mostly to the dietary importance of mollusks (Figure 3).

**Table 5:** Sensitivity risk values for nations with high to very high overall sensitivity to CC‐OA, showing indices that make up the sensitivity sublayer

Nation	Economic contribution	Nutritional contribution	Species sensitivity	Sensitivity
Developed				
France	**5**	4	1	4
Ireland	**5**	3	2	4
Italy	4	4	4	4
Japan	4	**5**	1	4
New Zealand	**5**	2	3	4
Portugal	4	4	2	4
Spain	**5**	4	2	4
Developing				
Antigua and Barbuda*		**5**		**5**
Bermuda*		4		4
China	3	**5**	2	4
Cook Islands*	**5**		**5**	**5**
Palau*	3		**5**	4
Peru	4	**5**	**5**	**5**
Thailand	3	4	3	4

Note

For table details (highlights, asterisk, missing values, and scoring), see Table 3 caption. See Table S10 for the full list of national sensitivities.

Eight of the 15 top mollusk‐producing nations scored high or very high overall sensitivity (Table 6). Peru was the most sensitive of top‐producing countries with high or very high scores for all sensitivity indices. Three of the top 15 producing nations were in Asia (China, Japan, and South Korea), with overall sensitivity primarily attributable to the nutritional contribution made by mollusks. In Europe, four top‐producing nations had high sensitivity due to high relative economic contribution; this also applied to New Zealand.

**Table 6:** Predicted sensitivity to CC‐OA for 15 largest producers (by weight) of mollusk mariculture in 2014 (FAO, 2016), including indices for economic and nutritional contribution and species sensitivity

Nation	Economic contribution	Nutritional contribution	Species sensitivity	Sensitivity
**China**	3	**5**	2	4
**Japan**	4	**5**	1	4
**South Korea**	3	**5**	2	4
Chile	3	2	2	3
**Spain**	**5**	4	2	4
Thailand	3	4	3	4
Vietnam	2	3	2	3
USA	4	3	2	3
**France**	**5**	4	1	4
**Italy**	**4**	4	4	4
**New Zealand**	**5**	2	3	4
Taiwan*	2		3	3
North Korea	3	2	2	3
**Netherlands**	**5**	2	2	3
**Peru**	4	**5**	**5**	**5**

Note

For other table details (highlights, asterisk, missing values, and scoring), see Table 3 caption.

### 3.3 Adaptive capacity

**Figure 4:**
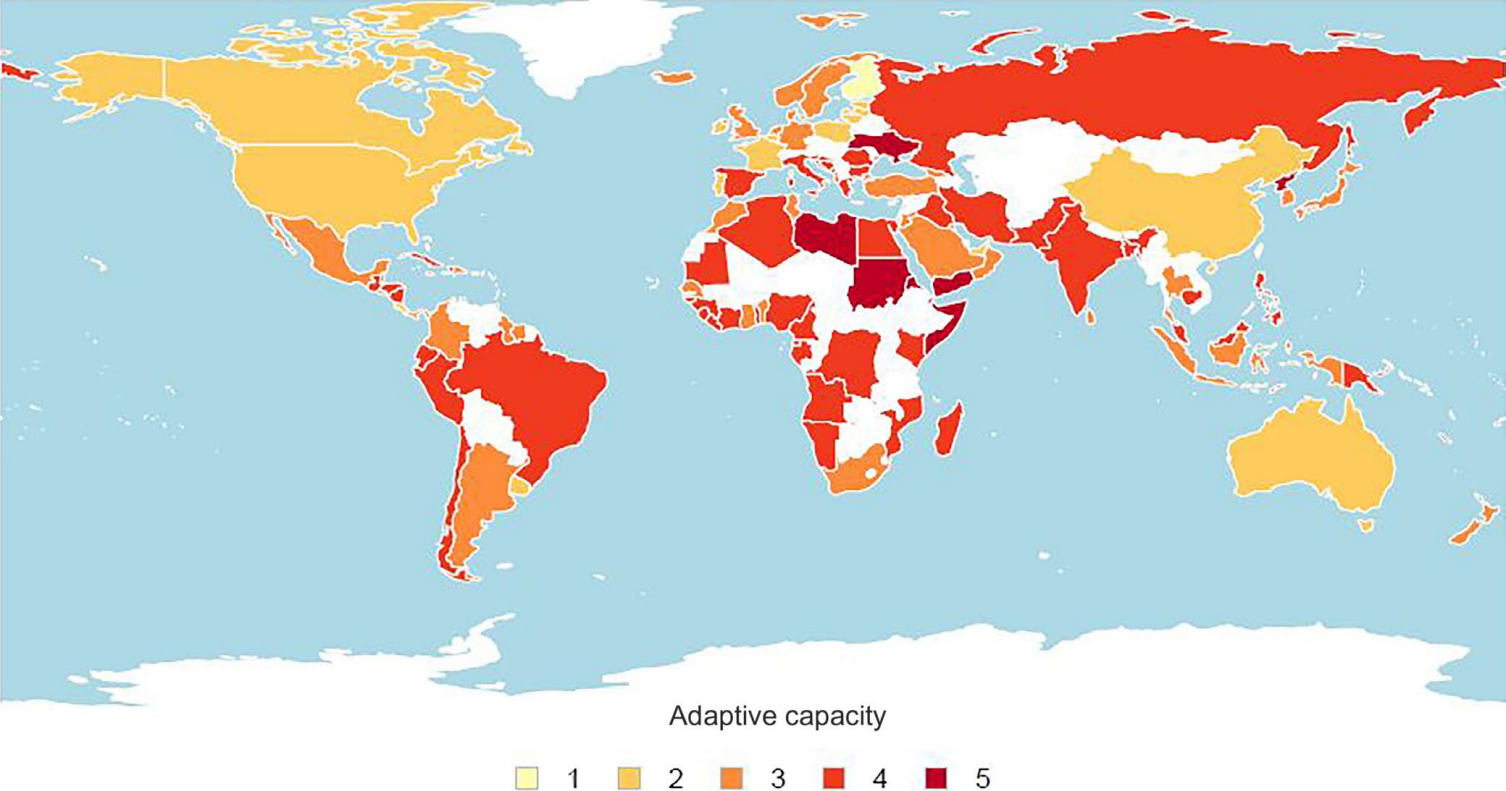
Adaptive capacity to CC‐OA for nations with current mollusk aquaculture operations, measured as nation‐specific mean of indices within the adaptive capacity sublayer. Colors represent adaptive capacity risk (1 = very low, 2 = low, 3 = moderate, 4 = high, and 5 = very high), with white where no data were available

Adaptive capacity, in terms of governance and bivalve mariculture diversity, was determined for 208 and 54 nations, respectively, giving a score for a total of 208 nations. PCA of the initial three indices showed that the first two principle components contributed 92% to the total variation and, given the high correlation (*r* = .75) between the governance and human development indices (HDI), the HDI was dropped. Eight of 14 nations that scored very high risk for adaptive capacity were missing data for industry diversity and had very high governance index impact scores (Figure 4; Table 7). These were mainly developing and least developed nations not currently practicing aquaculture. Scores for governance, by nation, are given in Table S11.

**Table 7:** Adaptive capacity sublayer indices for nations with very high overall adaptive capacity risk values

Nation	Governance	Industry diversity	Adaptive capacity
Developing			
Channel Islands*		**5**	5
Congo*	**5**		5
Cook Islands*		**5**	5
Eritrea*	**5**		5
Libya*	**5**		5
New Caledonia*		**5**	5
North Korea	**5**	**5**	5
Somalia*	**5**		5
St. Pierre and Miquelon*		**5**	5
Sudan*	**5**		5
Syrian Arab Republic*	**5**		5
Ukraine	4	**5**	5
Venezuela	4	**5**	5
Yemen*	**5**		5

Note

For table details (highlights, asterisk, missing values, and scoring), see Table 3 caption. For other nations, see Table S13.

Eight of the 15 top mollusk‐producing nations in the world scored very high in one or both of the indices within the adaptive capacity sublayer (Table 8); all eight scored very high risk for industry diversity and, with the exception of North Korea, were European (Table 8). These included many developed nations where the mollusk mariculture industry was dominated by one species (Denmark, Germany, Iceland, Netherlands, Sweden, United Kingdom all >94% *Mytilus edulis*; New Zealand 98% *Perna canaliculus,* and Norway 98% *Crassostrea gigas*; FAO, 2016).

**Table 8:** Predicted adaptive capacity to CC‐OA for 15 largest producers (listed in order of production output by weight in 2014; FAO, 2016), calculated as the mean of indices for governance and industry diversity

Nation	Governance	Industry diversity	Adaptive capacity
China	3	1	2
Japan	2	3	3
South Korea	2	3	3
**Chile**	2	**5**	4
**Spain**	2	**5**	4
Thailand	3	3	3
**Vietnam**	3	**5**	4
USA	2	2	2
France	2	2	2
**Italy**	3	**5**	4
**New Zealand**	1	**5**	3
Taiwan*	2		2
**North Korea**	**5**	**5**	**5**
**Netherlands**	1	**5**	3
**Peru**	3	**5**	4

Note

For other table details (bold emphasis, asterisk, missing values, and scoring), see Table 3 caption.

### 3.4 Overall vulnerability

**Figure 5:**
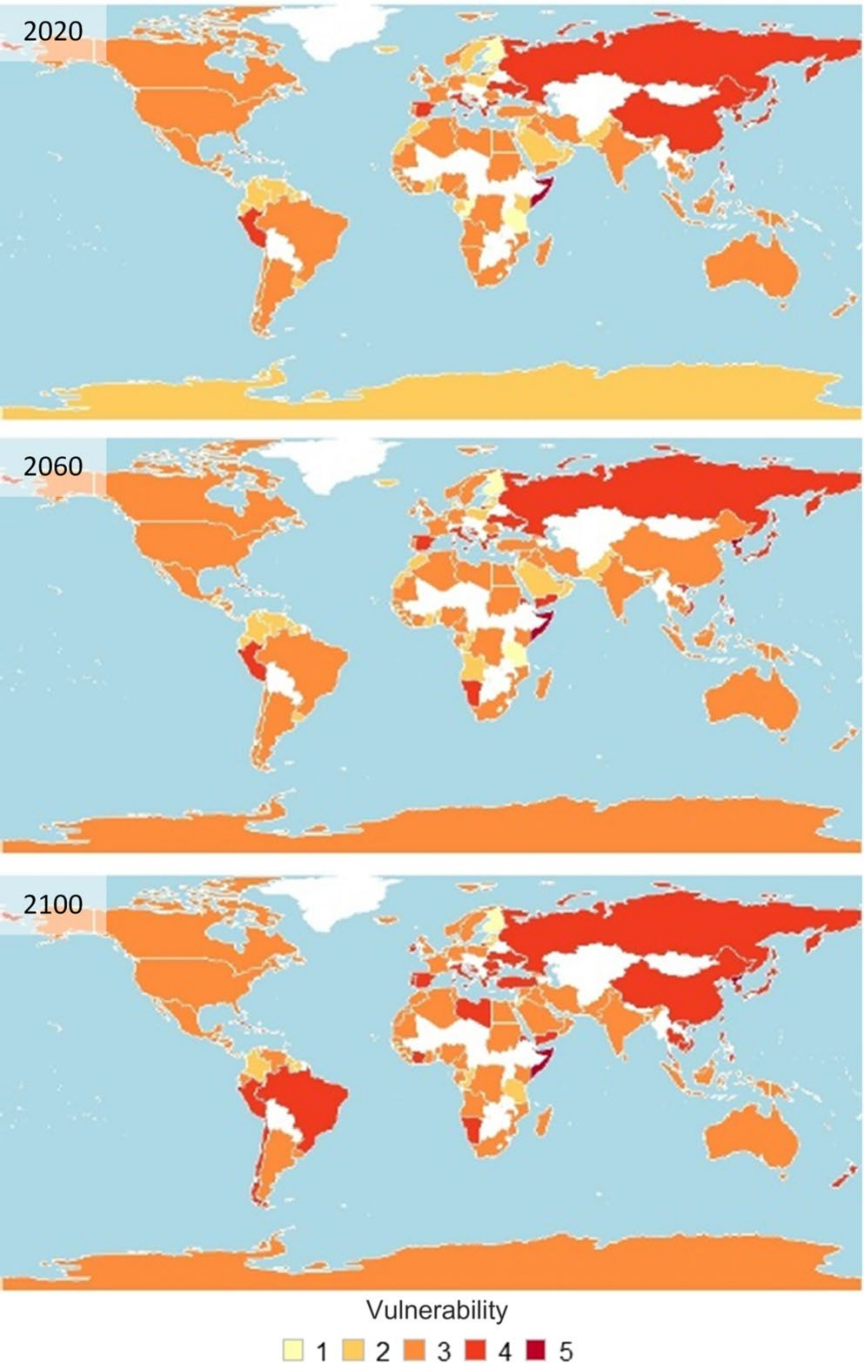
Overall vulnerability to CC‐OA for coastal nations, measured as nation‐specific mean for exposure, sensitivity, and adaptive capacity sublayers for decades 2020, 2060, and 2100. Colors represent overall vulnerability score, while white indicates the absence of data

Overall vulnerability was calculated for 117 coastal nations, for each decade from 2020 to 2100 (Figure 5). In general, predicted vulnerability increased over time to 2100 (Figure 5), with 26 nations predicted high or very high overall vulnerability by 2100 (Table 9). Of these, 17 were developing nations and 9 were developed nations (Table 9).

**Table 9:** Components of overall vulnerability for developed, developing, and least developed nations with high overall vulnerability in 2100

Nation	Exposure 2100	Sensitivity	Adaptive capacity
Developed			
Croatia	4	3	4
Greece	4	3	4
Ireland	4	4	2
Italy	4	4	4
**Japan**	5	4	3
New Zealand	3	4	3
Portugal	4	4	2
Romania	4	2	4
Spain	3	4	4
Developing			
**Antigua and Barbuda**	4	5	2
Brazil	3	3	4
Bulgaria	4	3	4
Chile	3	3	4
China	4	4	2
Cuba	3	3	4
**Libya**	4	1	5
Montenegro	4	3	4
Namibia	4	3	4
Palau	4	4	3
**Peru**	3	5	4
Philippines	4	3	4
Thailand	3	4	3
Tonga	4	3	4
**Turkey**	5	2	3
**Ukraine**	4	3	5
Least developed			
Cambodia	3	3	4

Note

There were no coastal nations with very high overall vulnerability. For other table details (bold emphasis, missing values, and scoring), see Table 3 caption.

By 2100, 12 of the 15 top mollusk‐producing nations were predicted to score high or very high for overall vulnerability (Table 10). Seven of these top‐producing nations are in Asia (China, Japan, South Korea, Thailand, Vietnam, Taiwan, and North Korea), with overall high vulnerability primarily attributable to high exposure (Table 9). However, four of these nations are missing data for one sublayer, which may contribute to their overall high vulnerability score (Table 10). Conversely, the classification of top‐producing nations in South America (Chile and Peru) and Europe (Spain and Italy) as high for overall vulnerability was attributed to their high risk for both the adaptive capacity and sensitivity (Table 9). France, the Netherlands, and the United States were the only top‐producing nations that were predicted to remain in the moderate overall vulnerability category by the year 2100 (Table 10).

**Table 10:** Predicted vulnerability to CC‐OA for the 15 largest producers of mollusks by mariculture in 2014 (FAO, 2016), including sublayers exposure (for the year 2100), current sensitivity, and current adaptive capacity

Nation	Exposure 2100	Sensitivity	Adaptive capacity	Vulnerability 2100
China	4	4	2	4
**Japan**	**5**	4	3	4
South Korea*	4		3	
Chile	3	3	4	4
Spain	3	4	4	4
Thailand	3	4	3	4
Vietnam*	**5**	**3**		
USA	4	3	2	3
France	3	4	2	3
Italy	4	4	4	4
New Zealand	3	4	3	4
Taiwan*	**5**	3		
North Korea*	4		**5**	
Netherlands	3	3	3	3
**Peru**	3	**5**	4	4

Note

For other table details (bold emphasis, missing values, and scoring), see Table 3 caption. Overall vulnerability could not be calculated for any nations missing data for any single sublayer, and these have been identified with an asterisk (*).

#### 3.4.1 Tipping points

**Figure 6:**
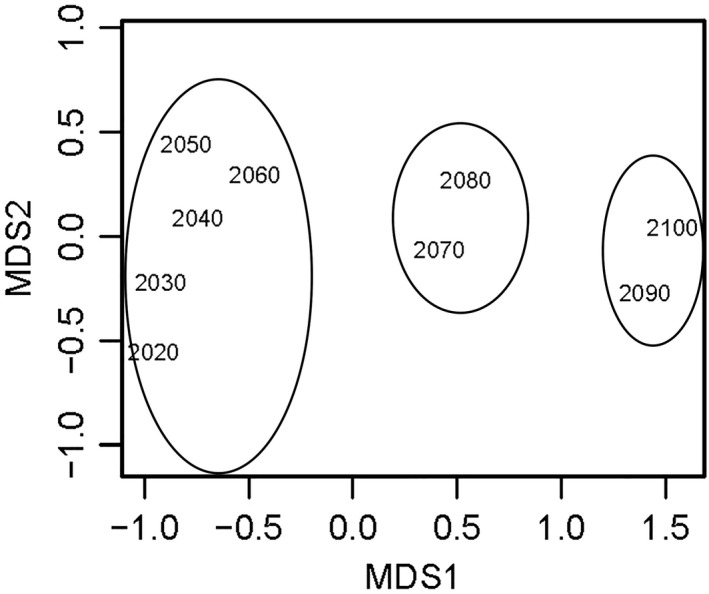
Nonmetric MDS of overall vulnerability to CC‐OA for all coastal, shellfish mariculture‐producing nations from 2020 to 2100. Clusters of decades most similar to each other are grouped. The greatest change (tipping point) occurs after 2060

**Figure 7:**
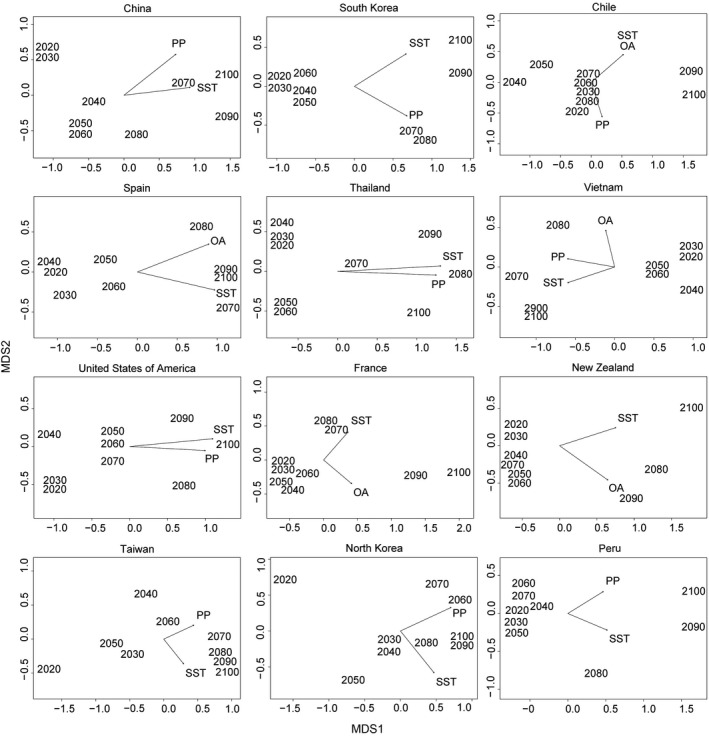
Nonmetric MDS of overall vulnerability for 12 top‐producing mollusk mariculture nations from 2020 to 2100. The strength and correlation between years and changes in sea surface temperature (SST), primary productivity (PP), and aragonite saturation (OA) are represented by arrows (e.g., for Thailand, the long arrows toward 2080 indicate that this ordination was driven by a strong, positive relationship between decade and increasing SST and declining PP, and that these stressors increased toward 2080/90/100). Stress was <0.010 in all nations with the exception of New Zealand, where it was 0.10

Nonmetric multidimensional scaling (MDS) of the overall vulnerability scores for each decade between 2020 and 2100 indicated a clear trend of change over time (Figure 6). When combined across all nations, the decades 2020–2060, 2070–2080, and 2090–2100 formed distinct groups (Figure 6). The period of greatest decline/threat to the global mollusk mariculture sector is predicted to begin in 2060 and continue to accelerate beyond 2080 (Figure 6). Predicted tipping points varied regionally but were earliest in South Korea and Vietnam, and Spain, beginning as early as 2020 in North Korea (Figure 7). Predicted tipping points for South America (Chile and Peru) occurred later than the global average (>2080; Figure 7).

Multidimensional scaling for vulnerability for the top 15 mollusk mariculture‐producing nations identified clear tipping points in 11 nations. MDS 2D‐stress values exceeded 0.20 for Japan, Italy, and the Netherlands, and these are not shown.

Sea surface temperature had the highest correlation with the MDS axis 1 for 11 of the 15 top‐producing mollusk mariculture nations (see Table S15). In some nations, this correlation was strong (i.e., *R* > .50; Chile, China, North Korea, Peru, South Korea, Spain, Thailand, United States, and Vietnam), while in others this correlation was weaker (Japan and the Netherlands; Table S15).

Clear tipping points at 2060 were predicted for South Korea and Vietnam, and this was associated with increases in sea surface temperature and declining primary productivity. In Europe, tipping points in Spain and France are predicted by 2060 and 2080, respectively, and were most closely correlated with sea surface temperature and aragonite saturation (Figure 7). Tipping points were unclear in other nations (Figure 7).

## 4 DISCUSSION

This study is the first of its kind to specifically assess and predict the unique challenges posed by CC‐OA to the global molluskan mariculture industry using the IPCC method for vulnerability assessments. Our global nation‐by‐nation scale perspective provides a context for finer scaled regional, local, or site‐specific analyses of the impacts of CC‐OA and provides an insight into complex and nonintuitive species‐specific spatiotemporal interactions that will play a key role in determining challenges for future global mollusk production. National‐ and international‐level predictions, of the type presented here, facilitate industry and governments in preparing strategies and policies that will mitigate the impacts of CC‐OA. Our approach complements that of Froehlich et al. (2018), but we collate data across nations and include current mollusk mariculture production and adaptive capacity in our assessment of national vulnerability.

Most (15/26) nations with predicted high to very high overall vulnerability were developing or least developed nations. This was largely attributable to both high exposure and low adaptive capacity in these nations, and it follows that increasing adaptive capacity in these vulnerable nations may be the most effective means of mitigating the impacts of CC‐OA to their shellfish mariculture industry (see adaptive capacity below). In addition, we predict that the mariculture industry in the current top‐producing nations will be challenged by CC‐OA for a variety of reasons and, consequently, meeting that challenge should be approached on a case‐by‐case basis. While vulnerability varied regionally and nationally, we predict that it will, generally, increase over time as a result of CC‐OA, with the largest changes beginning in 2060. However, within this general pattern we predict considerable variability.

### 4.1 Exposure

This study highlights that several, linked, CC‐OA‐related factors are likely to act synergistically, and detrimentally, to reduce molluskan mariculture production and that this is likely to increase over time but varies between nations. Our model predicted that existing molluskan mariculture is under the greatest threat in Asia (Japan, Taiwan, and Vietnam) linked primarily to declining primary productivity and high cyclone risk. This concurs with the finding of Froehlich et al. (2018) who linked predicted declines in primary productivity to a loss of suitable habitat for bivalves. Our exposure index captured the main CC‐OA‐related threats to mollusk shell production; it simultaneously accounted for changes in food availability, for example, allowing for the potential for mollusk to offset the effects of CC‐OA and thrive where food is abundant (Melzner et al., 2011). We predict that Georgia, Turkey, and Tunisia will experience the greatest challenge to future mollusk production from OA primarily because of a decline in aragonite saturation and, consequently, space in which successful culture can occur.

### 4.2 Sensitivity

Very high overall sensitivity was generally attributable to high species sensitivity within a given nation's mariculture industry. As such, one solution to the CC‐OA challenge could include switching to the culture of less sensitive species and/or moving culture operations to higher latitudes (Burrows et al., 2014), provided space, and markets exist. Nations that are characterized by limited cultivatable land (e.g., China) and those with deep‐seated cultural identities as “sea‐faring” nations (e.g., Antigua and Barbuda, Cook Islands, New Zealand, Thailand, French territories; Cooley et al., 2012) have tended to focus on marine sources of protein (FAO, 2014) and, as a consequence, such nations are more sensitive to challenges to molluskan mariculture (Cooley et al., 2012). China, with a growing population and concomitant food demand, is likely to face particular challenges.

### 4.3 Adaptive capacity

The adaptive capacity sublayer was composed of indices for industry diversity and governance. Countries with governance‐related low adaptive capacity may require additional support to maintain and develop mariculture operations (Blanchard et al., 2017). In general, high risk for adaptive capacity (8 of 14 nations) was attributed to poor governance as currently prevalent in developing and least developed nations in Africa. Climate change is likely to negatively impact upon terrestrial crop production in these regions necessitating consideration of other protein sources (Cooley et al., 2012; Lobell & Field, 2007), and this could include mollusks. Given the sustainability of molluskan mariculture (relatively low environmental impact, high‐quality, local market development, and employment opportunities), we recommend that future international development goals (e.g., UN Summit on Sustainable Development) for sustainable food production (UN, 2015) should encompass molluskan mariculture. Diversification of the molluskan‐production sector is recommended for many European nations where low mollusk‐culture species diversity reduces resilience and adaptability. Industry diversification will have its own challenges, particularly where markets for existing species are very entrenched and/or where the optimal culture species is non‐native and may pose an invasive‐risk (Callaway et al., 2012).

### 4.4 Overall vulnerability and tipping points

The consequences of a high overall vulnerability to CC‐OA in the molluskan mariculture sector will vary widely between nations. We predict that many of the current top mollusk‐producing nations are likely to be adversely affected by predicted CC‐OA impacts, but for different combinations of reasons. In South America, high overall vulnerability was attributable to low species diversity in Chile, while in Peru it was attributable to high species sensitivity and the dietary importance of shellfish. While not centered around shellfish mariculture, other vulnerability assessments have also found Chile to be a nation highly vulnerable to climate change (Handisyde et al., 2017). Our recommendation, particularly in these nations, is to diversify the industry and/or identify species that are better adapted to the projected CC‐OA‐related change.

In Europe, particularly Spain and Italy, high overall vulnerability was attributable to relatively high economic value of the mollusk mariculture sector. In addition, most of the European industry is based on the blue mussel (*Mytilus* sp), a habitat generalist and low sensitivity species. While *Mytilus* sp. might be robust against the threats, we identify here its dominance makes the European sector potentially vulnerable to indirect CC‐OA‐linked threats such as disease, harmful algal blooms, and invasive species. Our recommendation is to add adaptive capacity by diversifying the sector.

Asia hosts 7 of the 15 top mollusk‐producing nations (China, Japan, South Korea, Thailand, Vietnam, Taiwan, and North Korea) that together produce >98% of the world's total mollusk aquaculture production by weight (FAO, 2014). Our results concur with Froehlich et al. (2018) that many of these Asian nations are predicted to experience overall declines in the area suitable for shellfish cultivation making them vulnerable. These findings are in line with those made by Handisyde et al. (2017), which also found aquaculture in Asian nations including China, Thailand, and Vietnam to be highly vulnerable to climate change. Improvements in production infrastructure may mitigate against increases in extreme weather events, but other solutions will be required to address regional‐scale primary productivity declines. Consideration should be given to generating supplementary feed and selecting stock better adapted to lower levels of primary productivity.

We found that the majority of nations with high overall vulnerability were developing or least developed nations (*n* = 15) and that most of these nations did not currently host a substantive molluskan mariculture industry. High overall vulnerability in these developing/least developed nations was primarily attributable to high exposure and low adaptive capacity, and we predict that building adaptive capacity in these nations by improving governance and/or increasing the species diversity within any existing industry would be the best way to buffer the impacts of CC‐OA to their existing or future shellfish mariculture sector (see above adaptive capacity).

Tipping points indicate when major shifts in mariculture‐success may occur and, in our model, are based on existing industry patterns of production. Sea surface temperature was identified as the driver with the greatest association with tipping points. Our model predicted that overall vulnerability will increase in severity each decade as a result of CC‐OA and, considering the sector as a whole, that the largest decadal changes beginning in 2060 concurring with Froehlich et al. (2018). However, within this generalization, we predict Asian nations including North Korea will suffer CC‐OA‐related change as early as 2020 primarily driven by increases in sea surface temperature and declining primary productivity, while in South America (Chile and Peru) changes in sea surface temperature and aragonite saturation depth over time are predicted to cause the greatest change from 2080. These predictions are, again, in line with those of Froehlich et al. (2018).

### 4.5 Study challenges

Vulnerability assessment (VA) models, of the type and scope adopted here, are required to take complex, diverse data and simplify/reclassify and recombine it to make the goal of predicting overall vulnerability tractable (Adger, 2006). This VA considered the current vulnerability of shellfish mariculture, at a national level, to future climate change and as such, only the exposure sublayer is projected temporally to 2100. While a nation's sensitivity and adaptive capacity will change over time, our goal is to estimate vulnerability under present day conditions (e.g., of species diversity and socioeconomic status) to predict where the greatest vulnerability lies and where the greatest change in practice should be adopted.

While this study considered four environmental indicators, other CC‐OA‐linked factors such as disease, non‐native species, and future changes in trade practices may also have meaningful consequences for mollusk mariculture (Karvonen et al., 2010). This study necessarily adopted an indicator‐based approach but such approaches are based on relatively coarse spatial resolutions (e.g., of marine parameters), and are subject to gaps in data availability (Barsley et al., 2013 see below).

The species‐specific response to CC‐OA is complex and accurately predicting sensitivity is challenging, though habitat range (as adopted here) is a well‐recognized sensitivity indicator (Morrison et al., 2015). In this study, species sensitivity was based on adult habitat tolerances (i.e., the harvested stage of mollusks; Grassle, 2000). While CC‐OA will impact mollusks throughout their life cycle, juveniles are known to be particularly sensitive albeit to the same drivers (Morrison et al., 2015). In this respect, the results presented here may accurately represent patterns of CC‐OA‐induced change, but underestimate the magnitude, particularly for operations dependent upon wild‐sourced juveniles (Morrison et al., 2015).

Missing data are one of the most pervasive problems in trying to create balanced indicator‐based models (De Silva & Soto, 2009). In the current study, many nations were not represented by any of the data layers and most nations were missing information for one or more variables within each sublayer particularly those in developing countries or those without an active mariculture sector (see Table S16). In a similar fisheries‐based study using temperature change as the predictor, only 60 nations had sufficient data to calculate vulnerability (Allison, Perry, et al., 2009).

## CONFLICT OF INTEREST

None declared.

## AUTHOR CONTRIBUTIONS

PSS conceived the development, generated the model, prepared the R‐coding, and wrote the manuscript. KSL conceived the development and design, and revised and edited the manuscript. BLP prepared the R‐coding, and revised and edited the manuscript. TAW conceived the development and design, performed model refinement, and wrote and edited the manuscript.

## Supporting information

Supinfo

## Data Availability

The raw data are available at Dryad https://doi.org/10.5061/dryad.t4b8gthz3. Data associated with this paper were downloaded (November 2017) from the sources as referenced in the main text. These were as follows:
Exposure data: HADGEM2‐CC climate model, https://esgf-node.llnl.gov/projects/cmip5/, data under “CMIP5 Research.” Extreme weather events, “Global Cyclone Hazard Frequency and Distribution”: https://doi.org/10.7927/H4CZ353K
Maritime boundaries geodatabase: http://www.marineregions.org/downloads.php, data under “world EEZ.” Species sensitivity: https://obis.org/manual/access/, data under API, search for species by taxon name. Download.csv file. Molluskan mariculture production value: http://www.fao.org/fishery/statistics/en, data under “Global aquaculture production” accessed via online query, specify environment: “marine,” specify species: “mollusks” (excluding “Squids, cuttlefishes, octopuses”), specify time: 2012, 2013, and 2014. Download.csv file. Global GDP: https://data.worldbank.org/ , data under “Indicators,” then “GDP” (current US$), specify: 2012, 2013, and 2014. Download.csv file. Industry diversity in molluskan mariculture: http://www.fao.org/fishery/statistics/en data under “Global aquaculture production” accessed via online query, specify country: select all nations, specify environment: “marine,” specify species: “mollusks” (excluding “Squids, cuttlefishes, octopuses”), specify time: 2014. Download.csv file. Worldwide governance index: https://info.worldbank.org/governance/wgi/. Download full dataset. The data, as downloaded then transformed, are shown in Appendix S1, Tables S1–S16 (as referenced in main text).
