# Supplementary material for: A global assessment of the vulnerability of shellfish aquaculture to climate change and ocean acidification
Source: Ecol Evol. 2020 Mar 12;10(7):3518–34. doi: 10.1002/ece3.6149 (PMC7141013; doi:10.1002/ece3.6149)
Supplement: Supplementary file 1 — Supinfo [file ECE3-10-3518-s001.docx]

# SUPPLEMENTARY MATERIAL

# Exposure

### Sea surface temperature

Table S1: Exposure to sea surface temperature (°C) change from historical baseline temperatures (1985 - 2005), in the Exclusive Economic Zones (EEZ) of nations during decades from 2020 to 2100 as in Henson, Beaulieu et al. (2016). Exposure score was determined by change in °C where 1. Very-low = 0 to 2°C, 2. Low = 2 to 3°C, 3. Moderate = 3 to 4°C, 4. High = 4 to 5°C, and 5. Very-high = > 5°C. Future sea surface temperature values were projected using IPCC model RCP8.5 (Moss, Edmonds et al. 2010). Underpinning data is from the HadGEM-CC climate model (Collins, Bellouin et al. 2008) and is explained in the main text.

| **Nation** | **2011 to 2020** | **2021 to 2030** | **2031 to 2040** | **2041 to 2050** | **2051 to 2060** | **2061 to 2070** | **2071 to 2080** | **2081 to 2090** | **2091 to 2100** |
| --- | --- | --- | --- | --- | --- | --- | --- | --- | --- |
| Albania | 1 | 1 | 1 | 2 | 2 | 4 | 3 | 5 | 5 |
| Algeria | 1 | 1 | 1 | 2 | 2 | 4 | 3 | 4 | 5 |
| Angola | 1 | 1 | 1 | 1 | 1 | 2 | 2 | 2 | 3 |
| Antarctica | 1 | 1 | 1 | 1 | 1 | 1 | 1 | 1 | 1 |
| Antigua and Barbuda | 1 | 1 | 1 | 1 | 1 | 2 | 2 | 2 | 3 |
| Argentina | 1 | 1 | 1 | 1 | 1 | 1 | 1 | 2 | 2 |
| Australia | 1 | 1 | 1 | 1 | 1 | 1 | 2 | 2 | 2 |
| Azerbaijan | 1 | 1 | 1 | 1 | 2 | 4 | 3 | 3 | 4 |
| Bahrain | 1 | 1 | 1 | 2 | 2 | 4 | 3 | 3 | 4 |
| Bangladesh | 1 | 1 | 1 | 1 | 1 | 2 | 2 | 3 | 3 |
| Barbados | 1 | 1 | 1 | 1 | 1 | 2 | 2 | 2 | 3 |
| Belize | 1 | 1 | 1 | 1 | 1 | 2 | 2 | 3 | 3 |
| Benin | 1 | 1 | 1 | 1 | 1 | 2 | 3 | 3 | 3 |
| Brazil | 1 | 1 | 1 | 1 | 1 | 2 | 2 | 3 | 3 |
| Bulgaria | 1 | 1 | 1 | 1 | 2 | 4 | 3 | 4 | 5 |
| Cambodia | 1 | 1 | 1 | 1 | 1 | 2 | 3 | 2 | 3 |
| Cameroon | 1 | 1 | 1 | 1 | 1 | 2 | 3 | 3 | 3 |
| Canada | 1 | 1 | 1 | 1 | 2 | 4 | 4 | 4 | 5 |
| Cape Verde | 1 | 1 | 1 | 1 | 1 | 2 | 2 | 3 | 3 |
| Chile | 1 | 1 | 1 | 1 | 1 | 1 | 1 | 2 | 2 |
| China | 1 | 1 | 1 | 1 | 1 | 2 | 2 | 3 | 3 |
| Colombia | 1 | 1 | 1 | 1 | 1 | 2 | 2 | 3 | 3 |
| Comores |  | 1 | 1 | 1 | 1 | 1 | 2 | 2 | 2 |
| Costa Rica | 1 | 1 | 1 | 1 | 1 | 2 | 2 | 3 | 3 |
| Croatia | 1 | 1 | 1 | 2 | 2 | 4 | 3 | 5 | 5 |
| Cyprus | 1 | 1 | 1 | 1 | 2 | 2 | 2 | 3 | 4 |
| Democratic Republic of the Congo | 1 | 1 | 1 | 1 | 1 | 2 | 2 | 2 | 3 |
| Denmark | 1 | 1 | 1 | 1 | 1 | 1 | 1 | 2 | 2 |
| Djibouti | 1 | 1 | 1 | 1 | 2 | 2 | 3 | 3 | 3 |
| Dominica | 1 | 1 | 1 | 1 | 1 | 2 | 2 | 2 | 3 |
| Dominican Republic | 1 | 1 | 1 | 1 | 2 | 2 | 2 | 3 | 3 |
| Ecuador | 1 | 1 | 1 | 1 | 1 | 1 | 2 | 3 | 3 |
| Egypt | 1 | 1 | 1 | 1 | 2 | 2 | 2 | 3 | 4 |
| El Salvador | 1 | 1 | 1 | 1 | 1 | 2 | 2 | 3 | 3 |
| Equatorial Guinea | 1 | 1 | 1 | 1 | 1 | 2 | 2 | 3 | 3 |
| Eritrea | 1 | 1 | 1 | 2 | 2 | 4 | 3 | 4 | 4 |
| Federal Republic of Somalia | 1 | 1 | 1 | 1 | 1 | 2 | 3 | 3 | 3 |
| Fiji | 1 | 1 | 1 | 1 | 2 | 2 | 2 | 3 | 3 |
| France | 1 | 1 | 1 | 1 | 1 | 2 | 2 | 2 | 3 |
| Gabon | 1 | 1 | 1 | 1 | 1 | 2 | 2 | 3 | 3 |
| Gambia | 1 | 1 | 1 | 1 | 2 | 2 | 3 | 3 | 4 |
| Georgia | 1 | 1 | 1 | 1 | 2 | 4 | 3 | 4 | 5 |
| Germany | 1 | 1 | 1 | 2 | 3 | 4 | 4 | 4 | 5 |
| Ghana | 1 | 1 | 1 | 1 | 1 | 2 | 2 | 3 | 3 |
| Greece | 1 | 1 | 1 | 1 | 2 | 4 | 3 | 4 | 4 |
| Grenada | 1 | 1 | 1 | 1 | 1 | 2 | 2 | 3 | 3 |
| Guatemala | 1 | 1 | 1 | 1 | 1 | 2 | 2 | 3 | 3 |
| Guinea | 1 | 1 | 1 | 1 | 1 | 2 | 2 | 3 | 3 |
| Guinea-Bissau | 1 | 1 | 1 | 1 | 2 | 2 | 3 | 3 | 4 |
| Guyana | 1 | 1 | 1 | 1 | 1 | 2 | 2 | 3 | 3 |
| Haiti | 1 | 1 | 1 | 1 | 2 | 2 | 3 | 3 | 4 |
| Honduras | 1 | 1 | 1 | 1 | 1 | 2 | 2 | 3 | 3 |
| Iceland | 1 | 1 | 1 | 1 | 1 | 2 | 2 | 3 | 4 |
| India | 1 | 1 | 1 | 1 | 1 | 2 | 3 | 3 | 3 |
| Indonesia | 1 | 1 | 1 | 1 | 1 | 2 | 3 | 3 | 3 |
| Iran | 1 | 1 | 1 | 1 | 2 | 4 | 3 | 3 | 4 |
| Iraq | 1 | 1 | 1 | 2 | 2 | 4 | 3 | 3 | 5 |
| Ireland | 1 | 1 | 1 | 2 | 2 | 4 | 4 | 4 | 5 |
| Israel | 1 | 1 | 1 | 1 | 2 | 2 | 2 | 3 | 4 |
| Italy | 1 | 1 | 1 | 2 | 2 | 4 | 3 | 5 | 5 |
| Ivory Coast | 1 | 1 | 1 | 1 | 1 | 2 | 2 | 3 | 3 |
| Japan | 1 | 1 | 1 | 1 | 1 | 2 | 3 | 3 | 4 |
| Kazakhstan | 1 | 1 | 1 | 1 | 2 | 4 | 3 | 3 | 4 |
| Kenya |  | 1 | 1 | 1 | 1 | 2 | 3 | 3 | 3 |
| Kiribati | 1 | 1 | 1 | 2 | 2 | 2 | 3 | 3 | 4 |
| Kuwait | 1 | 1 | 1 | 2 | 2 | 4 | 3 | 3 | 5 |
| Lebanon | 1 | 1 | 1 | 1 | 2 | 2 | 2 | 3 | 4 |
| Liberia | 1 | 1 | 1 | 1 | 1 | 2 | 2 | 3 | 3 |
| Libya | 1 | 1 | 1 | 1 | 2 | 4 | 3 | 4 | 4 |
| Madagascar | 1 | 1 | 1 | 1 | 1 | 1 | 1 | 2 | 2 |
| Malaysia | 1 | 1 | 1 | 1 | 1 | 2 | 3 | 3 | 3 |
| Maldives | 1 | 1 | 1 | 1 | 2 | 2 | 3 | 3 | 4 |
| Malta | 1 | 1 | 1 | 1 | 2 | 4 | 3 | 4 | 4 |
| Marshall Islands | 1 | 1 | 1 | 1 | 1 | 2 | 3 | 3 | 3 |
| Mauritania | 1 | 1 | 1 | 1 | 1 | 2 | 2 | 2 | 3 |
| Mexico | 1 | 1 | 1 | 1 | 1 | 2 | 2 | 3 | 3 |
| Micronesia | 1 | 1 | 1 | 1 | 1 | 2 | 3 | 3 | 4 |
| Montenegro | 1 | 1 | 1 | 2 | 2 | 4 | 3 | 5 | 5 |
| Morocco | 1 | 1 | 1 | 1 | 2 | 4 | 3 | 4 | 4 |
| Mozambique | 1 | 1 | 1 | 1 | 1 | 1 | 2 | 2 | 2 |
| Myanmar | 1 | 1 | 1 | 1 | 1 | 2 | 3 | 3 | 3 |
| Namibia | 1 | 1 | 1 | 1 | 1 | 2 | 2 | 2 | 3 |
| Nauru | 1 | 1 | 1 | 2 | 2 | 4 | 3 | 3 | 4 |
| Netherlands | 1 | 1 | 1 | 1 | 2 | 4 | 3 | 3 | 4 |
| New Zealand | 1 | 1 | 1 | 1 | 1 | 1 | 2 | 2 | 3 |
| Nicaragua | 1 | 1 | 1 | 1 | 1 | 2 | 2 | 3 | 3 |
| Nigeria | 1 | 1 | 1 | 1 | 1 | 2 | 3 | 3 | 3 |
| North Korea | 1 | 2 | 2 | 2 | 3 | 4 | 3 | 4 | 4 |
| Norway | 1 | 1 | 1 | 1 | 1 | 1 | 1 | 2 | 2 |
| Oman | 1 | 1 | 1 | 1 | 2 | 2 | 3 | 3 | 4 |
| Pakistan | 1 | 1 | 1 | 1 | 1 | 2 | 2 | 2 | 2 |
| Palau | 1 | 1 | 1 | 1 | 1 | 2 | 3 | 3 | 3 |
| Panama | 1 | 1 | 1 | 1 | 1 | 2 | 2 | 3 | 3 |
| Papua New Guinea | 1 | 1 | 1 | 1 | 2 | 2 | 3 | 3 | 4 |
| Peru | 1 | 1 | 1 | 1 | 1 | 1 | 2 | 3 | 3 |
| Philippines | 1 | 1 | 1 | 1 | 1 | 2 | 2 | 2 | 3 |
| Poland | 1 | 1 | 1 | 2 | 3 | 4 | 4 | 4 | 5 |
| Portugal | 1 | 1 | 1 | 1 | 2 | 4 | 3 | 4 | 4 |
| Qatar | 1 | 1 | 1 | 2 | 2 | 4 | 4 | 4 | 5 |
| Republic of Mauritius | | 1 | 1 | 1 | 1 | 1 | 1 | 1 | 2 |
| Republic of the Congo | 1 | 1 | 1 | 1 | 1 | 2 | 2 | 3 | 3 |
| Romania | 1 | 1 | 1 | 2 | 2 | 4 | 3 | 4 | 5 |
| Russia | 1 | 1 | 1 | 1 | 1 | 1 | 1 | 2 | 2 |
| Saint Kitts and Nevis | 1 | 1 | 1 | 1 | 1 | 2 | 2 | 3 | 3 |
| Saint Lucia | 1 | 1 | 1 | 1 | 1 | 2 | 2 | 2 | 3 |
| Saint Vincent and the Grenadines | 1 | 1 | 1 | 1 | 1 | 2 | 2 | 3 | 3 |
| Samoa | 1 | 1 | 1 | 1 | 2 | 2 | 3 | 3 | 3 |
| Sao Tome and Principe | 1 | 1 | 1 | 1 | 1 | 2 | 3 | 3 | 3 |
| Saudi Arabia | 1 | 1 | 1 | 2 | 2 | 4 | 3 | 4 | 5 |
| Senegal | 1 | 1 | 1 | 1 | 2 | 2 | 2 | 3 | 3 |
| Seychelles |  | 1 | 1 | 1 | 1 | 1 | 2 | 2 | 3 |
| Sierra Leone | 1 | 1 | 1 | 1 | 1 | 2 | 2 | 3 | 3 |
| Solomon Islands | 1 | 1 | 1 | 1 | 2 | 2 | 2 | 3 | 3 |
| South Africa | 1 | 1 | 1 | 1 | 1 | 1 | 2 | 2 | 2 |
| South Korea | 1 | 1 | 1 | 1 | 1 | 2 | 2 | 3 | 3 |
| Spain | 1 | 1 | 1 | 2 | 2 | 4 | 3 | 4 | 4 |
| Sri Lanka | 1 | 1 | 1 | 1 | 1 | 2 | 3 | 3 | 4 |
| Sudan | 1 | 1 | 1 | 2 | 2 | 4 | 4 | 4 | 5 |
| Suriname | 1 | 1 | 1 | 1 | 1 | 2 | 2 | 3 | 3 |
| Sweden | 1 | 2 | 1 | 1 | 3 | 5 | 5 | 5 | 5 |
| Syria | 1 | 1 | 1 | 1 | 2 | 2 | 2 | 3 | 4 |
| Taiwan | 1 | 1 | 1 | 1 | 2 | 2 | 3 | 3 | 3 |
| Tanzania |  | 1 | 1 | 1 | 1 | 1 | 2 | 2 | 3 |
| Thailand | 1 | 1 | 1 | 1 | 1 | 2 | 3 | 3 | 3 |
| Togo | 1 | 1 | 1 | 1 | 1 | 2 | 3 | 3 | 3 |
| Tonga | 1 | 1 | 1 | 1 | 2 | 2 | 2 | 2 | 3 |
| Trinidad and Tobago | 1 | 1 | 1 | 1 | 1 | 2 | 2 | 3 | 3 |
| Tunisia | 1 | 1 | 1 | 2 | 2 | 4 | 3 | 5 | 5 |
| Turkey | 1 | 1 | 1 | 1 | 2 | 4 | 3 | 4 | 5 |
| Turkmenistan | 1 | 1 | 1 | 1 | 2 | 4 | 3 | 3 | 4 |
| Tuvalu | 1 | 1 | 1 | 1 | 2 | 2 | 3 | 3 | 3 |
| Ukraine | 1 | 1 | 1 | 1 | 2 | 4 | 3 | 4 | 5 |
| United Arab Emirates | 1 | 1 | 1 | 2 | 2 | 4 | 4 | 4 | 5 |
| United Kingdom | 1 | 1 | 1 | 1 | 1 | 2 | 2 | 2 | 3 |
| United States | 1 | 1 | 1 | 2 | 2 | 4 | 3 | 3 | 4 |
| Uruguay | 1 | 1 | 1 | 1 | 1 | 1 | 1 | 2 | 2 |
| Vanuatu | 1 | 1 | 1 | 1 | 2 | 2 | 2 | 3 | 3 |
| Venezuela | 1 | 1 | 1 | 1 | 1 | 2 | 2 | 3 | 3 |
| Vietnam | 1 | 1 | 1 | 1 | 1 | 2 | 3 | 2 | 3 |
| Western Sahara | 1 | 1 | 1 | 1 | 2 | 2 | 2 | 3 | 3 |
| Yemen | 1 | 1 | 1 | 1 | 1 | 2 | 2 | 3 |  |

### Aragonite saturation horizon depth (m)

Table S2: Exposure to change in aragonite saturation horizon depth (m) from historical baseline aragonite sauration horizon (1985 - 2005), in the Exclusive Economic Zones (EEZ) of nations during decades from 2020 to 2100 as in **Henson, Beaulieu et al. (2016)**. Exposure score was determined by decline in % of aragonite saturation horizon (m) where 1. Very-low = < 0%, 2. Low = 0 to 25%, 3. Moderate = 25 to 50% 4. High = 50 to 75% and 5. Very-high = 75 to 100%. Future aragonite sauration horizon depth values were projected using IPCC model RCP8.5 (Moss, Edmonds et al. 2010). Underpinning data is from the HadGEM-CC climate model (Collins, Bellouin et al. 2008) and is explained in the main text.

| **Nation** | **2011 to 2020** | **2021 to 2030** | **2031 to 2040** | **2041 to 2050** | **2051 to 2060** | **2061 to 2070** | **2071 to 2080** | **2081 to 2090** | **2091 to 2100** |
| --- | --- | --- | --- | --- | --- | --- | --- | --- | --- |
| Albania | 2 | 2 | 2 | 4 | 5 | 5 | 5 | 5 | 5 |
| Algeria | 5 | 4 | 4 | 4 | 5 | 5 | 5 | 5 | 5 |
| Angola | 1 | 1 | 1 | 1 | 1 | 1 | 1 | 1 | 1 |
| Antarctica | 2 | 2 | 2 | 3 | 5 | 5 | 5 | 5 | 5 |
| Antigua and Barbuda | 2 | 2 | 2 | 2 | 3 | 3 | 3 | 3 | 3 |
| Argentina | 1 | 1 | 1 | 1 | 2 | 2 | 2 | 3 | 4 |
| Australia | 3 | 3 | 3 | 3 | 3 | 3 | 3 | 3 | 3 |
| Azerbaijan | 5 | 5 | 5 | 5 | 5 | 5 | 5 | 5 | 5 |
| Bahrain | 2 | 2 | 2 | 3 | 5 | 5 | 5 | 5 | 5 |
| Barbados | 1 | 1 | 1 | 1 | 1 | 1 | 1 | 1 | 1 |
| Belize | 2 | 2 | 2 | 2 | 2 | 2 | 2 | 2 | 2 |
| Benin | 2 | 2 | 2 | 2 | 2 | 2 | 3 | 3 | 3 |
| Brazil | 1 | 1 | 1 | 1 | 1 | 1 | 1 | 1 | 1 |
| Bulgaria | 5 | 5 | 5 | 5 | 5 | 5 | 5 | 5 | 5 |
| Cameroon | 2 | 2 | 2 | 2 | 2 | 2 | 2 | 3 | 3 |
| Canada | 5 | 5 | 5 | 5 | 5 | 5 | 5 | 5 | 5 |
| Cape Verde | 1 | 1 | 1 | 1 | 1 | 1 | 1 | 1 | 1 |
| Chile | 2 | 2 | 2 | 2 | 2 | 2 | 2 | 3 | 3 |
| China | 5 | 4 | 4 | 4 | 4 | 4 | 4 | 4 | 4 |
| Colombia | 1 | 1 | 1 | 1 | 1 | 1 | 1 | 1 | 1 |
| Comores | 1 | 1 | 1 | 1 | 1 | 1 | 1 | 1 | 1 |
| Costa Rica | 2 | 2 | 2 | 2 | 2 | 3 | 3 | 3 | 3 |
| Croatia | 3 | 3 | 5 | 5 | 5 | 5 | 5 | 5 | 5 |
| Cyprus | 1 | 1 | 1 | 1 | 1 | 1 | 1 | 1 | 1 |
| Democratic Republic of the Congo | 1 | 1 | 1 | 1 | 1 | 1 | 1 | 1 | 1 |
| Djibouti | 2 | 2 | 2 | 2 | 2 | 2 | 2 | 2 | 2 |
| Dominica | 1 | 1 | 1 | 1 | 1 | 1 | 1 | 1 | 1 |
| Ecuador | 1 | 1 | 1 | 1 | 1 | 1 | 1 | 1 | 1 |
| Egypt | 1 | 1 | 1 | 1 | 1 | 1 | 1 | 1 | 1 |
| El Salvador | 1 | 1 | 1 | 1 | 1 | 1 | 1 | 1 | 1 |
| Equatorial Guinea | 1 | 1 | 1 | 1 | 1 | 1 | 1 | 1 | 1 |
| Eritrea | 1 | 1 | 1 | 1 | 1 | 1 | 1 | 1 | 1 |
| Federal Republic of Somalia | 1 | 1 | 1 | 1 | 1 | 1 | 1 | 1 | 1 |
| Fiji | 3 | 3 | 3 | 3 | 3 | 3 | 3 | 3 | 3 |
| France | 2 | 2 | 2 | 2 | 2 | 2 | 2 | 3 | 3 |
| Gabon | 1 | 1 | 1 | 1 | 1 | 1 | 1 | 1 | 1 |
| Gambia | 2 | 2 | 2 | 2 | 2 | 2 | 2 | 2 | 2 |
| Georgia | 5 | 5 | 5 | 5 | 5 | 5 | 5 | 5 | 5 |
| Ghana | 1 | 1 | 1 | 1 | 2 | 2 | 2 | 2 | 2 |
| Greece | 3 | 3 | 3 | 3 | 3 | 4 | 4 | 4 | 4 |
| Grenada | 1 | 1 | 1 | 1 | 1 | 1 | 1 | 1 | 1 |
| Guatemala | 1 | 1 | 1 | 1 | 1 | 1 | 1 | 1 | 1 |
| Guinea | 1 | 1 | 1 | 1 | 1 | 1 | 1 | 1 | 1 |
| Guinea-Bissau | 1 | 1 | 1 | 1 | 1 | 1 | 1 | 1 | 1 |
| Guyana | 1 | 1 | 1 | 1 | 1 | 1 | 1 | 1 | 1 |
| Honduras | 1 | 1 | 1 | 1 | 1 | 1 | 1 | 1 | 1 |
| India | 1 | 1 | 1 | 1 | 1 | 1 | 1 | 1 | 1 |
| Indonesia | 2 | 2 | 2 | 2 | 2 | 3 | 3 | 3 | 3 |
| Iran | 1 | 1 | 1 | 2 | 2 | 2 | 2 | 2 | 2 |
| Iraq | 2 | 2 | 2 | 2 | 3 | 3 | 4 | 4 | 5 |
| Ireland | 1 | 1 | 1 | 2 | 5 | 5 | 5 | 5 | 5 |
| Israel | 1 | 1 | 1 | 1 | 1 | 1 | 1 | 1 | 1 |
| Italy | 2 | 2 | 3 | 3 | 4 | 4 | 4 | 4 | 4 |
| Ivory Coast | 1 | 1 | 1 | 1 | 1 | 1 | 1 | 2 | 2 |
| Japan | 5 | 4 | 4 | 4 | 4 | 4 | 4 | 4 | 4 |
| Kazakhstan | 5 | 5 | 5 | 5 | 5 | 5 | 5 | 5 | 5 |
| Kenya | 1 | 1 | 1 | 1 | 1 | 1 | 1 | 1 | 1 |
| Kiribati | 5 | 4 | 4 | 4 | 4 | 4 | 5 | 5 | 4 |
| Kuwait | 2 | 2 | 2 | 2 | 2 | 2 | 2 | 2 | 3 |
| Lebanon | 1 | 1 | 1 | 1 | 1 | 1 | 1 | 1 | 1 |
| Liberia | 1 | 1 | 1 | 1 | 1 | 2 | 2 | 2 | 2 |
| Libya | 2 | 2 | 2 | 3 | 3 | 3 | 3 | 4 | 4 |
| Madagascar | 2 | 2 | 2 | 2 | 2 | 2 | 2 | 3 | 3 |
| Malaysia | 1 | 1 | 1 | 1 | 1 | 1 | 1 | 1 | 1 |
| Maldives | 1 | 1 | 1 | 1 | 1 | 1 | 1 | 1 | 1 |
| Malta | 5 | 5 | 5 | 5 | 5 | 5 | 5 | 5 | 5 |
| Marshall Islands | 5 | 4 | 4 | 4 | 4 | 4 | 4 | 4 | 4 |
| Mauritania | 1 | 1 | 1 | 1 | 1 | 1 | 1 | 1 | 1 |
| Mexico | 2 | 2 | 2 | 2 | 2 | 2 | 2 | 2 | 2 |
| Micronesia | 5 | 4 | 4 | 4 | 4 | 4 | 4 | 4 | 4 |
| Montenegro | 5 | 5 | 5 | 5 | 5 | 5 | 5 | 5 | 5 |
| Morocco | 1 | 1 | 1 | 1 | 1 | 1 | 1 | 1 | 1 |
| Mozambique | 2 | 2 | 3 | 3 | 3 | 3 | 3 | 4 | 4 |
| Namibia | 3 | 3 | 3 | 3 | 3 | 3 | 3 | 4 | 4 |
| Nauru | 5 | 4 | 4 | 5 | 5 | 5 | 5 | 5 | 5 |
| Netherlands | 1 | 1 | 1 | 1 | 1 | 1 | 1 | 1 | 1 |
| New Zealand | 3 | 3 | 3 | 3 | 3 | 3 | 4 | 4 | 4 |
| Nicaragua | 1 | 1 | 1 | 1 | 1 | 1 | 1 | 1 | 1 |
| Nigeria | 2 | 2 | 2 | 2 | 2 | 2 | 2 | 3 | 3 |
| North Korea | 5 | 5 | 5 | 5 | 5 | 5 | 5 | 5 | 5 |
| Norway | 2 | 2 | 2 | 2 | 3 | 3 | 3 | 4 | 5 |
| Oman | 1 | 1 | 1 | 1 | 1 | 1 | 1 | 1 | 1 |
| Pakistan | 1 | 1 | 1 | 1 | 1 | 1 | 1 | 1 | 1 |
| Palau | 5 | 4 | 4 | 4 | 4 | 4 | 4 | 4 | 4 |
| Papua New Guinea | 5 | 4 | 4 | 4 | 4 | 4 | 5 | 5 | 4 |
| Peru | 2 | 2 | 2 | 2 | 2 | 2 | 2 | 2 | 2 |
| Philippines | 5 | 4 | 4 | 4 | 4 | 4 | 4 | 4 | 4 |
| Portugal | 3 | 3 | 3 | 3 | 4 | 4 | 4 | 4 | 4 |
| Qatar | 2 | 2 | 2 | 2 | 2 | 2 | 2 | 2 | 3 |
| Republic of Mauritius | 1 | 1 | 1 | 1 | 1 | 1 | 1 | 1 | 1 |
| Republic of the Congo | 1 | 1 | 1 | 1 | 1 | 1 | 1 | 1 | 1 |
| Romania | 2 | 2 | 5 | 5 | 5 | 5 | 5 | 5 | 5 |
| Russia | 5 | 4 | 5 | 5 | 5 | 5 | 5 | 5 | 5 |
| Saint Kitts and Nevis | 2 | 2 | 2 | 2 | 2 | 2 | 2 | 2 | 2 |
| Saint Lucia | 2 | 2 | 2 | 2 | 2 | 3 | 3 | 3 | 3 |
| Saint Vincent and the Grenadines | 2 | 2 | 2 | 2 | 2 | 2 | 2 | 2 | 2 |
| Samoa | 3 | 3 | 3 | 3 | 3 | 3 | 3 | 3 | 3 |
| Sao Tome and Principe | 1 | 1 | 1 | 1 | 1 | 1 | 1 | 1 | 1 |
| Saudi Arabia | 1 | 1 | 1 | 1 | 1 | 1 | 1 | 1 | 1 |
| Senegal | 1 | 1 | 1 | 1 | 1 | 1 | 1 | 1 | 1 |
| Seychelles | 1 | 1 | 1 | 1 | 1 | 1 | 1 | 1 | 1 |
| Sierra Leone | 1 | 1 | 1 | 1 | 1 | 1 | 1 | 1 | 1 |
| Solomon Islands | 5 | 4 | 4 | 4 | 4 | 4 | 4 | 4 | 4 |
| South Africa | 1 | 1 | 1 | 1 | 1 | 1 | 1 | 1 | 1 |
| South Korea | 5 | 5 | 5 | 5 | 5 | 5 | 5 | 5 | 5 |
| Spain | 1 | 1 | 1 | 1 | 1 | 2 | 2 | 2 | 2 |
| Sri Lanka | 1 | 1 | 1 | 1 | 1 | 1 | 1 | 1 | 1 |
| Sudan | 1 | 1 | 1 | 1 | 1 | 1 | 1 | 1 | 1 |
| Suriname | 1 | 1 | 1 | 1 | 1 | 1 | 1 | 1 | 1 |
| Syria | 5 | 5 | 5 | 5 | 5 | 5 | 5 | 5 | 5 |
| Taiwan | 5 | 4 | 4 | 4 | 4 | 4 | 4 | 4 | 4 |
| Tanzania | 1 | 1 | 1 | 1 | 1 | 1 | 1 | 1 | 1 |
| Thailand | 1 | 1 | 1 | 1 | 1 | 1 | 1 | 1 | 1 |
| Togo | 2 | 2 | 2 | 2 | 2 | 2 | 2 | 2 | 3 |
| Tonga | 3 | 3 | 3 | 3 | 3 | 3 | 3 | 3 | 3 |
| Tunisia | 5 | 4 | 4 | 5 | 5 | 5 | 5 | 5 | 5 |
| Turkey | 5 | 5 | 5 | 5 | 5 | 5 | 5 | 5 | 5 |
| Turkmenistan | 5 | 5 | 5 | 5 | 5 | 5 | 5 | 5 | 5 |
| Tuvalu | 5 | 4 | 4 | 4 | 5 | 5 | 5 | 5 | 4 |
| Ukraine | 1 | 1 | 5 | 5 | 5 | 5 | 5 | 5 | 5 |
| United Arab Emirates | 1 | 1 | 1 | 1 | 1 | 1 | 1 | 1 | 1 |
| United Kingdom | 1 | 1 | 1 | 1 | 1 | 2 | 2 | 2 | 3 |
| United States | 3 | 3 | 3 | 3 | 3 | 3 | 4 | 4 | 3 |
| Uruguay | 3 | 3 | 3 | 3 | 3 | 3 | 3 | 4 | 4 |
| Vanuatu | 3 | 3 | 3 | 3 | 3 | 3 | 3 | 3 | 3 |
| Venezuela | 1 | 1 | 1 | 1 | 1 | 1 | 1 | 1 | 1 |
| Western Sahara | 1 | 1 | 1 | 1 | 1 | 1 | 1 | 1 | 1 |
| Yemen | 1 | 1 | 1 | 1 | 1 | 1 | 1 | 1 | 1 |

### Primary productivity

Table S3: Exposure to change in Primary Organic Carbon Production (PP) (mol m^-2^ s^-1^) from historical baseline PP (1985 - 2005), in the Exclusive Economic Zones (EEZ) of nations during decades from 2020 to 2100 as in Henson, Beaulieu et al. (2016). Exposure score was determined by change in % of PP where 1. Very-low = < 0%, 2. Low = 0 to 25%, 3. Moderate = 25 to 50% 4. High = 50 to 75% and 5. Very-high = 75 to 100%. Future PP values were projected using IPCC model RCP8.5 (Moss, Edmonds et al. 2010). Underpinning data is from the HadGEM-CC climate model (Collins, Bellouin et al. 2008) and is explained in the main text.

| **Nation** | **2011 to 2020** | **2021 to 2030** | **2031 to 2040** | **2041 to 2050** | **2051 to 2060** | **2061 to 2070** | **2071 to 2080** | **2081 to 2090** | **2091 to 2100** |
| --- | --- | --- | --- | --- | --- | --- | --- | --- | --- |
| Albania | 2 | 2 | 1 | 2 | 1 | 2 | 1 | 3 | 2 |
| Algeria | 2 | 2 | 2 | 2 | 2 | 2 | 2 | 3 | 2 |
| Angola | 2 | 1 | 1 | 1 | 1 | 1 | 1 | 1 | 1 |
| Antarctica | 1 | 1 | 1 | 1 | 1 | 1 | 1 | 1 | 1 |
| Antigua and Barbuda | 2 | 2 | 2 | 2 | 2 | 2 | 3 | 3 | 2 |
| Argentina | 2 | 2 | 2 | 2 | 2 | 2 | 2 | 2 | 2 |
| Australia | 2 | 2 | 2 | 2 | 2 | 2 | 2 | 2 | 2 |
| Azerbaijan | 1 | 1 | 2 | 1 | 1 | 1 | 2 | 1 | 3 |
| Bahrain | 2 | 2 | 4 | 3 | 3 | 4 | 4 | 2 | 3 |
| Bangladesh | 3 | 3 | 3 | 4 | 4 | 5 | 5 | 5 | 5 |
| Barbados | 2 | 2 | 2 | 1 | 1 | 1 | 2 | 2 | 1 |
| Belize | 2 | 2 | 2 | 2 | 2 | 2 | 2 | 3 | 2 |
| Benin | 2 | 2 | 2 | 3 | 3 | 3 | 3 | 3 | 4 |
| Brazil | 2 | 2 | 2 | 2 | 2 | 2 | 2 | 2 | 3 |
| Bulgaria | 2 | 2 | 2 | 2 | 1 | 2 | 2 | 2 | 2 |
| Cambodia | 2 | 2 | 3 | 3 | 3 | 4 | 4 | 4 | 4 |
| Cameroon | 2 | 2 | 2 | 3 | 3 | 3 | 3 | 3 | 3 |
| Canada | 1 | 1 | 2 | 1 | 1 | 1 | 1 | 1 | 1 |
| Cape Verde | 3 | 3 | 4 | 4 | 4 | 4 | 5 | 4 | 3 |
| Chile | 2 | 2 | 1 | 1 | 2 | 2 | 2 | 2 | 2 |
| China | 2 | 2 | 2 | 2 | 2 | 3 | 2 | 3 | 3 |
| Colombia | 1 | 1 | 2 | 2 | 1 | 1 | 1 | 2 | 2 |
| Comores | 1 | 1 | 1 | 1 | 1 | 1 | 1 | 1 | 1 |
| Costa Rica | 2 | 2 | 2 | 2 | 2 | 2 | 2 | 2 | 2 |
| Croatia | 2 | 2 | 2 | 2 | 1 | 2 | 1 | 2 | 2 |
| Cyprus | 4 | 3 | 3 | 3 | 3 | 4 | 1 | 3 | 3 |
| Democratic Republic of the Congo | 1 | 1 | 1 | 1 | 1 | 1 | 2 | 1 | 1 |
| Denmark | 1 | 1 | 1 | 1 | 1 | 1 | 1 | 1 | 1 |
| Djibouti | 2 | 2 | 3 | 2 | 2 | 3 | 3 | 3 | 3 |
| Dominica | 2 | 3 | 3 | 2 | 3 | 3 | 4 | 4 | 3 |
| Dominican Republic | 3 | 3 | 3 | 3 | 3 | 3 | 3 | 4 | 3 |
| Ecuador | 1 | 1 | 1 | 1 | 1 | 1 | 1 | 2 | 1 |
| Egypt | 2 | 2 | 3 | 2 | 2 | 2 | 1 | 1 | 1 |
| El Salvador | 2 | 2 | 1 | 1 | 1 | 2 | 2 | 3 | 2 |
| Equatorial Guinea | 1 | 2 | 2 | 2 | 2 | 2 | 2 | 2 | 2 |
| Eritrea | 1 | 1 | 3 | 3 | 2 | 3 | 3 | 2 | 3 |
| Federal Republic of Somalia | 1 | 2 | 2 | 2 | 2 | 2 | 2 | 2 | 3 |
| Fiji | 2 | 2 | 2 | 2 | 2 | 3 | 2 | 3 | 3 |
| France | 2 | 2 | 2 | 2 | 2 | 2 | 2 | 2 | 2 |
| Gabon | 1 | 1 | 2 | 2 | 2 | 2 | 2 | 2 | 2 |
| Gambia | 1 | 1 | 4 | 5 | 4 | 4 | 4 | 5 | 4 |
| Georgia | 2 | 3 | 2 | 2 | 2 | 2 | 2 | 2 | 3 |
| Germany | 2 | 2 | 2 | 2 | 2 | 2 | 2 | 2 | 2 |
| Ghana | 2 | 2 | 2 | 3 | 2 | 3 | 3 | 3 | 3 |
| Greece | 3 | 3 | 2 | 3 | 3 | 3 | 2 | 4 | 3 |
| Grenada | 2 | 3 | 3 | 2 | 3 | 2 | 3 | 4 | 3 |
| Guatemala | 2 | 1 | 1 | 2 | 1 | 2 | 2 | 2 | 2 |
| Guinea | 2 | 2 | 3 | 4 | 3 | 4 | 4 | 3 | 3 |
| Guinea-Bissau | 2 | 2 | 4 | 4 | 3 | 4 | 4 | 4 | 4 |
| Guyana | 2 | 1 | 2 | 2 | 1 | 1 | 1 | 1 | 1 |
| Haiti | 3 | 2 | 2 | 2 | 3 | 3 | 3 | 3 | 3 |
| Honduras | 2 | 2 | 2 | 2 | 2 | 2 | 2 | 2 | 2 |
| Iceland | 1 | 1 | 1 | 1 | 1 | 1 | 1 | 1 | 1 |
| India | 1 | 2 | 2 | 2 | 2 | 2 | 2 | 2 | 2 |
| Indonesia | 2 | 2 | 2 | 2 | 2 | 2 | 3 | 2 | 3 |
| Iran | 2 | 2 | 2 | 2 | 2 | 3 | 3 | 2 | 3 |
| Iraq | 1 | 2 | 3 | 3 | 1 | 3 | 1 | 1 | 2 |
| Ireland | 1 | 2 | 2 | 2 | 2 | 2 | 2 | 2 | 2 |
| Israel | 1 | 1 | 1 | 1 | 1 | 1 | 1 | 1 | 1 |
| Italy | 2 | 2 | 2 | 2 | 2 | 2 | 1 | 3 | 2 |
| Ivory Coast | 2 | 2 | 2 | 3 | 2 | 3 | 3 | 3 | 3 |
| Japan | 2 | 2 | 2 | 2 | 3 | 3 | 3 | 4 | 4 |
| Kazakhstan | 1 | 2 | 2 | 2 | 1 | 2 | 2 | 2 | 3 |
| Kenya | 1 | 1 | 2 | 2 | 2 | 2 | 2 | 2 | 2 |
| Kiribati | 2 | 2 | 2 | 3 | 3 | 3 | 3 | 3 | 3 |
| Kuwait | 1 | 1 | 3 | 2 | 1 | 3 | 1 | 1 | 1 |
| Lebanon | 2 | 2 | 2 | 2 | 1 | 2 | 1 | 1 | 1 |
| Liberia | 2 | 2 | 2 | 3 | 3 | 3 | 3 | 3 | 3 |
| Libya | 2 | 3 | 2 | 3 | 4 | 4 | 2 | 4 | 3 |
| Madagascar | 1 | 1 | 1 | 1 | 1 | 1 | 1 | 1 | 1 |
| Malaysia | 1 | 2 | 2 | 2 | 2 | 2 | 2 | 2 | 2 |
| Maldives | 1 | 2 | 2 | 2 | 2 | 2 | 2 | 2 | 2 |
| Malta | 2 | 3 | 2 | 3 | 3 | 4 | 2 | 4 | 3 |
| Marshall Islands | 2 | 1 | 2 | 2 | 2 | 2 | 3 | 2 | 3 |
| Mauritania | 3 | 4 | 5 | 3 | 4 | 4 | 5 | 5 | 4 |
| Mexico | 2 | 2 | 2 | 2 | 2 | 2 | 2 | 2 | 2 |
| Micronesia | 2 | 2 | 2 | 2 | 3 | 3 | 3 | 3 | 3 |
| Montenegro | 2 | 2 | 2 | 2 | 1 | 2 | 1 | 2 | 2 |
| Morocco | 2 | 2 | 2 | 2 | 2 | 3 | 2 | 3 | 3 |
| Mozambique | 1 | 2 | 1 | 2 | 1 | 1 | 2 | 1 | 1 |
| Myanmar | 2 | 2 | 3 | 3 | 4 | 4 | 4 | 5 | 5 |
| Namibia | 1 | 4 | 4 | 4 | 4 | 4 | 4 | 4 | 4 |
| Nauru | 2 | 2 | 2 | 2 | 3 | 3 | 3 | 3 | 3 |
| Netherlands | 2 | 2 | 2 | 2 | 2 | 2 | 2 | 2 | 2 |
| New Zealand | 2 | 2 | 2 | 2 | 2 | 2 | 2 | 2 | 2 |
| Nicaragua | 2 | 2 | 2 | 2 | 2 | 2 | 2 | 3 | 2 |
| Nigeria | 2 | 2 | 2 | 3 | 3 | 3 | 3 | 3 | 3 |
| North Korea | 1 | 2 | 2 | 2 | 2 | 2 | 2 | 2 | 2 |
| Norway | 2 | 1 | 1 | 1 | 1 | 1 | 1 | 1 | 1 |
| Oman | 1 | 1 | 1 | 3 | 2 | 3 | 3 | 3 | 3 |
| Pakistan | 1 | 1 | 1 | 1 | 1 | 2 | 2 | 2 | 3 |
| Palau | 2 | 2 | 2 | 2 | 3 | 3 | 3 | 3 | 4 |
| Panama | 2 | 2 | 2 | 2 | 2 | 2 | 2 | 3 | 2 |
| Papua New Guinea | 2 | 2 | 2 | 2 | 3 | 3 | 3 | 3 | 3 |
| Peru | 1 | 1 | 1 | 1 | 1 | 1 | 1 | 2 | 2 |
| Philippines | 2 | 2 | 2 | 3 | 3 | 3 | 3 | 4 | 4 |
| Poland | 2 | 2 | 2 | 2 | 2 | 2 | 2 | 2 | 2 |
| Portugal | 2 | 2 | 2 | 2 | 2 | 2 | 2 | 2 | 2 |
| Qatar | 1 | 2 | 4 | 3 | 4 | 4 | 4 | 3 | 4 |
| Republic of Mauritius | 1 | 1 | 1 | 1 | 1 | 1 | 2 | 2 | 1 |
| Republic of the Congo | 1 | 2 | 1 | 2 | 2 | 1 | 2 | 2 | 2 |
| Romania | 2 | 2 | 2 | 2 | 1 | 2 | 2 | 2 | 2 |
| Russia | 2 | 2 | 2 | 2 | 2 | 2 | 2 | 2 | 3 |
| Saint Kitts and Nevis | 3 | 3 | 3 | 3 | 3 | 3 | 4 | 4 | 3 |
| Saint Lucia | 2 | 3 | 3 | 2 | 3 | 3 | 4 | 4 | 3 |
| Saint Vincent and the Grenadines | 2 | 3 | 3 | 2 | 3 | 2 | 3 | 4 | 3 |
| Samoa | 2 | 2 | 2 | 2 | 2 | 3 | 3 | 3 | 3 |
| Sao Tome and Principe | 1 | 2 | 2 | 2 | 2 | 2 | 2 | 2 | 3 |
| Saudi Arabia | 1 | 1 | 1 | 3 | 2 | 1 | 2 | 2 | 2 |
| Senegal | 2 | 2 | 4 | 5 | 3 | 4 | 4 | 4 | 4 |
| Seychelles | 1 | 1 | 2 | 2 | 2 | 2 | 2 | 2 | 2 |
| Sierra Leone | 2 | 2 | 2 | 3 | 3 | 4 | 3 | 3 | 3 |
| Solomon Islands | 2 | 2 | 2 | 2 | 2 | 3 | 3 | 3 | 3 |
| South Africa | 2 | 2 | 2 | 2 | 2 | 2 | 2 | 2 | 2 |
| South Korea | 2 | 2 | 2 | 2 | 2 | 3 | 3 | 3 | 3 |
| Spain | 2 | 2 | 2 | 2 | 2 | 2 | 2 | 2 | 2 |
| Sri Lanka | 1 | 2 | 2 | 2 | 2 | 2 | 3 | 2 | 3 |
| Sudan | 2 | 1 | 1 | 2 | 1 | 1 | 2 | 1 | 2 |
| Suriname | 2 | 1 | 2 | 1 | 1 | 1 | 1 | 1 | 1 |
| Sweden | 1 | 1 | 1 | 1 | 1 | 1 | 1 | 1 | 1 |
| Syria | 3 | 3 | 3 | 3 | 3 | 4 | 1 | 3 | 2 |
| Taiwan | 2 | 3 | 4 | 3 | 4 | 5 | 5 | 5 | 5 |
| Tanzania | 1 | 1 | 1 | 1 | 1 | 1 | 2 | 2 | 1 |
| Thailand | 2 | 2 | 2 | 2 | 2 | 2 | 3 | 3 | 3 |
| Togo | 2 | 2 | 2 | 3 | 3 | 3 | 3 | 3 | 3 |
| Tonga | 2 | 2 | 2 | 2 | 2 | 2 | 2 | 3 | 3 |
| Trinidad and Tobago | 2 | 2 | 2 | 2 | 2 | 1 | 2 | 3 | 1 |
| Tunisia | 2 | 2 | 2 | 2 | 3 | 3 | 2 | 3 | 3 |
| Turkey | 2 | 3 | 2 | 2 | 2 | 2 | 2 | 3 | 3 |
| Turkmenistan | 1 | 1 | 2 | 1 | 1 | 1 | 2 | 1 | 2 |
| Tuvalu | 2 | 2 | 2 | 2 | 2 | 3 | 3 | 3 | 3 |
| Ukraine | 2 | 2 | 2 | 2 | 2 | 2 | 2 | 2 | 2 |
| United Arab Emirates | 1 | 3 | 4 | 3 | 4 | 4 | 4 | 3 | 5 |
| United Kingdom | 2 | 2 | 2 | 2 | 2 | 2 | 2 | 2 | 2 |
| United States | 2 | 2 | 2 | 2 | 2 | 3 | 3 | 3 | 3 |
| Uruguay | 2 | 1 | 1 | 1 | 2 | 2 | 2 | 2 | 2 |
| Vanuatu | 2 | 2 | 2 | 3 | 3 | 3 | 3 | 3 | 3 |
| Venezuela | 2 | 3 | 3 | 2 | 3 | 2 | 3 | 4 | 3 |
| Vietnam | 2 | 2 | 3 | 3 | 4 | 4 | 4 | 5 | 5 |
| Western Sahara | 2 | 2 | 3 | 3 | 3 | 3 | 3 | 3 | 3 |
| Yemen | 1 | 2 | 2 | 2 | 2 | 3 | 3 | 3 | 3 |

### Cyclone risk

Table S4: Exposure to cyclone risk based on cyclone track data from the ‘Center for Hazards and Risk Research’ (CHRR) at Columbia University, with a layer showing cyclone risk based on event-frequency over the period 1980 - 2000 (CCCMA 2016). Mean CHRR score for each nations EEZ were linearly reclassified from the CHRR risk-deciles (scale of 1 – 10) to our impact scale of 1 – 5 (1 = Very-low, 2 = Low, 3 = Moderate, 4 = High, 5 = Very-high).

| **Nation** | **Mean CHRR score** | **Cyclone risk** |
| --- | --- | --- |
| Antigua and Barbuda | 9.0 | 5 |
| Australia | 4.6 | 3 |
| Bahamas | 7.5 | 5 |
| Bangladesh | 5.0 | 3 |
| Barbados | 1.0 | 1 |
| Belize | 8.3 | 5 |
| Cambodia | 1.0 | 1 |
| Canada | 3.1 | 3 |
| Cape Verde | 1.0 | 1 |
| China | 8.0 | 5 |
| Colombia | 1.4 | 2 |
| Comores | 4.4 | 3 |
| Costa Rica | 1.0 | 1 |
| Cuba | 4.8 | 3 |
| Dominica | 8.0 | 4 |
| Dominican Republic | 6.4 | 4 |
| East Timor | 1.0 | 1 |
| El Salvador | 1.0 | 1 |
| Fiji | 7.0 | 4 |
| France | 6.3 | 4 |
| Grenada | 1.5 | 2 |
| Guatemala | 1.0 | 1 |
| Haiti | 3.0 | 2 |
| Honduras | 3.8 | 3 |
| India | 3.1 | 3 |
| Indonesia | 1.7 | 2 |
| Ireland | 1.0 | 1 |
| Jamaica | 2.7 | 2 |
| Japan | 9.7 | 5 |
| Madagascar | 8.1 | 5 |
| Mexico | 4.6 | 3 |
| Micronesia | 6.5 | 4 |
| Mozambique | 6.0 | 4 |
| Myanmar | 1.7 | 2 |
| Netherlands | 3.0 | 2 |
| New Zealand | 2.3 | 2 |
| Nicaragua | 2.8 | 2 |
| North Korea | 6.1 | 4 |
| Pakistan | 1.0 | 1 |
| Palau | 2.2 | 2 |
| Panama | 1.0 | 1 |
| Papua New Guinea | 3.4 | 3 |
| Philippines | 9.1 | 5 |
| Portugal | 3.8 | 3 |
| Republic of Mauritius | 10.0 | 5 |
| Russia | 4.5 | 3 |
| Saint Kitts and Nevis | 8.0 | 4 |
| Saint Lucia | 1.4 | 2 |
| Saint Vincent and the Grenadines | 2.2 | 2 |
| Samoa | 3.0 | 2 |
| Solomon Islands | 5.8 | 4 |
| South Korea | 8.9 | 5 |
| Spain | 1.0 | 1 |
| Sri Lanka | 4.8 | 3 |
| Taiwan | 10.0 | 5 |
| Thailand | 1.0 | 2 |
| Tonga | 6.8 | 4 |
| Trinidad and Tobago | 2.6 | 2 |
| Tuvalu | 1.0 | 1 |
| United Kingdom | 3.2 | 2 |
| United States | 7.0 | 4 |
| Vanuatu | 9.1 | 5 |
| Venezuela | 1.1 | 2 |
| Vietnam | 7.4 | 5 |

## Overall Exposure

Table S5: Overall Exposure to CC-OA for nations with current mollusc aquaculture operations, measured as nation-specific mean of indices within the Exposure sub-layer from 2020 - 2100, for IPCC scenario RCP8.5. Exposure scores are 1 = Very-low, 2 = Low, 3 = Moderate, 4 = High, 5 = Very-high. Underpinning data is from the HadGEM-CC climate model (Collins, Bellouin et al. 2008) and is explained in the main text. Blank cells indicate missing data.

| **Nation** | **2020** | **2030** | **2040** | **2050** | **2060** | **2070** | **2080** | **2090** | **2100** |
| --- | --- | --- | --- | --- | --- | --- | --- | --- | --- |
| Albania | 2 | 2 | 2 | 3 | 3 | 4 | 3 | 5 | 4 |
| Algeria | 3 | 3 | 3 | 3 | 3 | 4 | 4 | 4 | 4 |
| Angola | 2 | 1 | 1 | 1 | 1 | 2 | 2 | 2 | 2 |
| Antarctica | 2 | 2 | 2 | 2 | 3 | 3 | 3 | 3 | 3 |
| Antigua and Barbuda | 3 | 3 | 3 | 2 | 3 | 3 | 4 | 4 | 4 |
| Argentina | 2 | 2 | 2 | 2 | 2 | 2 | 2 | 3 | 3 |
| Australia | 3 | 3 | 3 | 2 | 3 | 3 | 3 | 3 | 3 |
| Bahamas | 5 | 5 | 5 | 3 | 5 | 5 | 5 | 5 | 5 |
| Bahrain | 2 | 2 | 3 | 3 | 4 | 5 | 4 | 4 | 4 |
| Bangladesh | 3 | 3 | 3 | 1 | 3 | 4 | 4 | 4 | 4 |
| Barbados | 2 | 2 | 2 | 2 | 1 | 2 | 2 | 2 | 2 |
| Belize | 3 | 3 | 3 | 2 | 3 | 3 | 3 | 4 | 3 |
| Benin | 2 | 2 | 2 | 2 | 2 | 3 | 3 | 3 | 4 |
| Brazil | 2 | 2 | 2 | 3 | 2 | 2 | 2 | 2 | 3 |
| Bulgaria | 3 | 3 | 3 | 2 | 3 | 4 | 4 | 4 | 4 |
| Cambodia | 2 | 2 | 2 | 2 | 2 | 3 | 3 | 3 | 3 |
| Cameroon | 2 | 2 | 2 | 3 | 2 | 3 | 3 | 3 | 3 |
| Canada | 3 | 3 | 3 | 2 | 3 | 4 | 4 | 4 | 4 |
| Cape Verde | 2 | 2 | 2 | 2 | 2 | 2 | 3 | 3 | 2 |
| Chile | 2 | 2 | 2 | 3 | 2 | 2 | 2 | 3 | 3 |
| China | 4 | 3 | 3 | 2 | 3 | 4 | 4 | 4 | 4 |
| Colombia | 2 | 2 | 2 | 2 | 2 | 2 | 2 | 2 | 2 |
| Costa Rica | 2 | 2 | 2 | 3 | 2 | 2 | 2 | 3 | 3 |
| Croatia | 2 | 2 | 3 | 2 | 3 | 4 | 3 | 4 | 4 |
| Cuba | 3 | 3 | 3 | 1 | 3 | 3 | 3 | 3 | 3 |
| Cyprus | 2 | 2 | 2 | 1 | 2 | 3 | 2 | 3 | 3 |
| Democratic Republic of the Congo | 1 | 1 | 1 | 2 | 1 | 2 | 2 | 2 | 2 |
| Denmark | 1 | 1 | 1 | 2 | 1 | 1 | 1 | 2 | 2 |
| Djibouti | 2 | 2 | 2 | 2 | 2 | 3 | 3 | 3 | 3 |
| Dominica | 2 | 3 | 3 | 1 | 3 | 3 | 3 | 3 | 3 |
| Dominican Republic | 3 | 3 | 3 | 2 | 3 | 3 | 3 | 4 | 4 |
| Ecuador | 1 | 1 | 1 | 1 | 1 | 1 | 2 | 2 | 2 |
| Egypt | 2 | 2 | 2 | 2 | 2 | 2 | 2 | 2 | 2 |
| El Salvador | 2 | 2 | 1 | 2 | 1 | 2 | 2 | 2 | 2 |
| Equatorial Guinea | 1 | 2 | 2 | 2 | 2 | 2 | 2 | 2 | 2 |
| Eritrea | 1 | 1 | 2 | 2 | 2 | 3 | 3 | 3 | 3 |
| Fiji | 3 | 3 | 3 | 2 | 3 | 3 | 3 | 4 | 4 |
| France | 3 | 3 | 3 | 3 | 3 | 3 | 3 | 3 | 3 |
| Gabon | 1 | 1 | 2 | 3 | 2 | 2 | 2 | 2 | 2 |
| Gambia | 2 | 2 | 3 | 2 | 3 | 3 | 3 | 4 | 4 |
| Georgia | 3 | 3 | 3 | 2 | 3 | 4 | 4 | 4 | 5 |
| Germany | 2 | 2 | 2 | 3 | 3 | 3 | 3 | 3 | 4 |
| Ghana | 2 | 2 | 2 | 2 | 2 | 3 | 3 | 3 | 3 |
| Greece | 3 | 3 | 2 | 2 | 3 | 4 | 3 | 4 | 4 |
| Grenada | 2 | 2 | 2 | 2 | 2 | 2 | 2 | 3 | 3 |
| Guatemala | 2 | 1 | 1 | 2 | 1 | 2 | 2 | 2 | 2 |
| Guinea | 2 | 2 | 2 | 2 | 2 | 3 | 3 | 3 | 3 |
| Guinea-Bissau | 2 | 2 | 2 | 2 | 2 | 3 | 3 | 3 | 3 |
| Guyana | 2 | 1 | 2 | 2 | 1 | 2 | 2 | 2 | 2 |
| Haiti | 2 | 2 | 2 | 1 | 3 | 3 | 3 | 3 | 3 |
| Honduras | 2 | 2 | 2 | 2 | 2 | 2 | 2 | 3 | 3 |
| Iceland | 1 | 1 | 1 | 2 | 1 | 2 | 2 | 2 | 3 |
| India | 2 | 2 | 2 | 2 | 2 | 2 | 3 | 3 | 3 |
| Indonesia | 2 | 2 | 2 | 3 | 2 | 3 | 3 | 3 | 3 |
| Iran | 2 | 2 | 2 | 2 | 2 | 3 | 3 | 3 | 3 |
| Iraq | 2 | 2 | 2 | 1 | 2 | 4 | 3 | 3 | 4 |
| Ireland | 1 | 2 | 2 | 3 | 3 | 3 | 3 | 3 | 4 |
| Israel | 1 | 1 | 1 | 2 | 2 | 2 | 2 | 2 | 2 |
| Italy | 2 | 2 | 2 | 3 | 3 | 4 | 3 | 4 | 4 |
| Ivory Coast | 2 | 2 | 2 | 2 | 2 | 2 | 2 | 3 | 3 |
| Jamaica | 2 | 2 | 2 | 3 | 2 | 2 | 2 | 2 | 2 |
| Japan | 4 | 3 | 3 | 2 | 4 | 4 | 4 | 4 | 5 |
| Kenya | 1 | 1 | 2 | 2 | 2 | 2 | 2 | 2 | 2 |
| Kiribati | 3 | 3 | 3 | 2 | 3 | 3 | 4 | 4 | 4 |
| Kuwait | 2 | 2 | 2 | 3 | 2 | 3 | 2 | 2 | 3 |
| Lebanon | 2 | 2 | 2 | 2 | 2 | 2 | 2 | 2 | 2 |
| Liberia | 2 | 2 | 2 | 2 | 2 | 3 | 3 | 3 | 3 |
| Libya | 2 | 2 | 2 | 2 | 3 | 4 | 3 | 4 | 4 |
| Madagascar | 3 | 3 | 3 | 3 | 3 | 3 | 3 | 3 | 3 |
| Malaysia | 1 | 2 | 2 | 3 | 2 | 2 | 2 | 2 | 2 |
| Maldives | 1 | 2 | 2 | 2 | 2 | 2 | 2 | 2 | 3 |
| Malta | 3 | 3 | 3 | 2 | 4 | 5 | 4 | 5 | 4 |
| Marshall Islands | 3 | 2 | 3 | 3 | 3 | 3 | 4 | 3 | 4 |
| Mauritania | 2 | 2 | 3 | 3 | 2 | 3 | 3 | 3 | 3 |
| Mexico | 2 | 2 | 2 | 2 | 2 | 3 | 3 | 3 | 3 |
| Micronesia | 3 | 3 | 3 | 2 | 3 | 4 | 4 | 4 | 4 |
| Montenegro | 3 | 3 | 3 | 3 | 3 | 4 | 3 | 4 | 4 |
| Morocco | 2 | 2 | 2 | 3 | 2 | 3 | 2 | 3 | 3 |
| Mozambique | 2 | 3 | 3 | 2 | 3 | 3 | 3 | 3 | 3 |
| Namibia | 2 | 3 | 3 | 2 | 3 | 3 | 3 | 4 | 4 |
| Nauru | 3 | 3 | 3 | 2 | 4 | 4 | 4 | 4 | 4 |
| Netherlands | 2 | 2 | 2 | 2 | 2 | 3 | 2 | 2 | 3 |
| New Zealand | 2 | 2 | 2 | 3 | 2 | 2 | 3 | 3 | 3 |
| Nicaragua | 2 | 2 | 2 | 2 | 2 | 2 | 2 | 3 | 2 |
| Nigeria | 2 | 2 | 2 | 2 | 2 | 3 | 3 | 3 | 3 |
| North Korea | 3 | 4 | 4 | 1 | 4 | 4 | 4 | 4 | 4 |
| Norway | 2 | 2 | 2 | 3 | 2 | 2 | 2 | 3 | 3 |
| Oman | 1 | 1 | 1 | 2 | 2 | 2 | 3 | 3 | 3 |
| Pakistan | 1 | 1 | 1 | 3 | 1 | 2 | 2 | 2 | 2 |
| Palau | 3 | 3 | 3 | 2 | 3 | 3 | 3 | 3 | 4 |
| Panama | 2 | 2 | 2 | 3 | 2 | 2 | 2 | 3 | 2 |
| Papua New Guinea | 3 | 3 | 3 | 2 | 3 | 3 | 4 | 4 | 4 |
| Peru | 2 | 2 | 2 | 2 | 2 | 2 | 2 | 3 | 3 |
| Philippines | 4 | 3 | 3 | 3 | 4 | 4 | 4 | 4 | 4 |
| Poland | 2 | 2 | 2 | 2 | 3 | 3 | 3 | 3 | 4 |
| Portugal | 3 | 3 | 3 | 3 | 3 | 4 | 3 | 4 | 4 |
| Qatar | 2 | 2 | 3 | 3 | 3 | 4 | 4 | 3 | 4 |
| Republic of the Congo | 1 | 2 | 1 | 2 | 2 | 2 | 2 | 2 | 2 |
| Romania | 2 | 2 | 3 | 2 | 3 | 4 | 4 | 4 | 4 |
| Russia | 3 | 3 | 3 | 2 | 3 | 3 | 3 | 3 | 4 |
| Saint Kitts and Nevis | 3 | 3 | 3 | 2 | 3 | 3 | 3 | 4 | 3 |
| Saint Lucia | 2 | 2 | 2 | 2 | 2 | 3 | 3 | 3 | 3 |
| St Vincent and the Grenadines | 2 | 2 | 2 | 2 | 2 | 2 | 3 | 3 | 3 |
| Samoa | 2 | 2 | 2 | 3 | 3 | 3 | 3 | 3 | 3 |
| Sao Tome and Principe | 1 | 2 | 2 | 2 | 2 | 2 | 2 | 2 | 3 |
| Saudi Arabia | 1 | 1 | 1 | 2 | 2 | 2 | 2 | 3 | 3 |
| Senegal | 2 | 2 | 2 | 3 | 2 | 3 | 3 | 3 | 3 |
| Seychelles | 1 | 1 | 2 | 2 | 2 | 2 | 2 | 2 | 2 |
| Sierra Leone | 2 | 2 | 2 | 3 | 2 | 3 | 2 | 3 | 3 |
| Solomon Islands | 3 | 3 | 3 | 2 | 3 | 4 | 4 | 4 | 4 |
| South Africa | 2 | 2 | 2 | 2 | 2 | 2 | 2 | 2 | 2 |
| South Korea | 4 | 4 | 4 | 2 | 4 | 4 | 4 | 4 | 4 |
| Spain | 2 | 2 | 2 | 1 | 2 | 3 | 2 | 3 | 3 |
| Sri Lanka | 2 | 2 | 2 | 1 | 2 | 2 | 3 | 3 | 3 |
| Sudan | 2 | 1 | 1 | 3 | 2 | 2 | 3 | 2 | 3 |
| Suriname | 2 | 1 | 2 | 3 | 1 | 2 | 2 | 2 | 2 |
| Sweden | 1 | 1 | 1 | 1 | 2 | 3 | 3 | 3 | 3 |
| Syria | 3 | 3 | 3 | 2 | 4 | 4 | 3 | 4 | 4 |
| Taiwan | 4 | 4 | 4 | 2 | 4 | 4 | 5 | 5 | 5 |
| Tanzania | 1 | 1 | 1 | 2 | 1 | 1 | 2 | 2 | 2 |
| Thailand | 2 | 2 | 2 | 2 | 2 | 2 | 3 | 3 | 3 |
| Togo | 2 | 2 | 2 | 3 | 2 | 3 | 3 | 3 | 3 |
| Tonga | 3 | 3 | 3 | 3 | 3 | 3 | 3 | 3 | 4 |
| Trinidad and Tobago | 2 | 2 | 2 | 3 | 2 | 2 | 2 | 3 | 2 |
| Tunisia | 3 | 3 | 3 | 3 | 4 | 4 | 4 | 5 | 5 |
| Turkey | 3 | 3 | 3 | 2 | 3 | 4 | 4 | 4 | 5 |
| Tuvalu | 3 | 2 | 2 | 2 | 3 | 3 | 3 | 3 | 3 |
| Ukraine | 2 | 2 | 3 | 3 | 3 | 4 | 4 | 4 | 4 |
| United Arab Emirates | 1 | 2 | 2 | 2 | 3 | 3 | 3 | 3 | 4 |
| United Kingdom | 2 | 2 | 2 | 3 | 2 | 2 | 2 | 2 | 3 |
| United States | 3 | 3 | 3 | 2 | 3 | 4 | 4 | 4 | 4 |
| Uruguay | 2 | 2 | 2 | 2 | 2 | 2 | 2 | 3 | 3 |
| Vanuatu | 3 | 3 | 3 | 2 | 4 | 4 | 4 | 4 | 4 |
| Venezuela | 2 | 2 | 2 | 2 | 2 | 2 | 2 | 3 | 3 |
| Vietnam | 3 | 3 | 3 |  | 4 | 4 | 4 | 4 | 5 |
| Western Sahara | 2 | 2 | 2 |  | 2 | 2 | 2 | 3 | 3 |
| Yemen | 1 | 2 | 2 |  | 2 | 2 | 2 | 3 | 2 |

# Sensitivity

### Species Sensitivity

Table S6: To build the species sensitivity index for mollusc species cultured in mariculture operations globally, data on the distribution of species in terms of depth, temperature, and salinity were collated from the Ocean Biogeographic Information System (Grassle 2000). Habitat tolerances for congenerics were used in the absence of species-level data. Missing data were ignored when generating average species sensitivity values for each nation. *Represent taxa that have genus information used in absence of species level data.

| **Common name** | **Scientific name** | **Min depth (m)** | **Max depth (m)** | **Min temp (°C)** | **Max temp (°C)** | **Min salinity (PPS)** | **Max salinity (PPS)** |
| --- | --- | --- | --- | --- | --- | --- | --- |
| Queen scallop | *Aequipecten opercularis* | 0 | 160 | 5 | 20 | 30 | 40 |
| Grand ark | *Anadara grandis** | 0 | 50 | 5 | 30 | 15 | 35 |
| Peruvian calico scallop | *Argopecten purpuratus* | 0 | 20 | 15 | 20 | 30 | 35 |
| Pen shells nei | *Atrina spp.* | 0 | 40 | 10 | 30 | 30 | 40 |
| Cholga mussel | *Aulacomya ater* | 0 | 90 | -5 | 15 | 30 | 35 |
| Clams, etc. nei | Bivalvia | 0 | 2000 | -5 | 30 | 5 | 40 |
| Cockles nei | Cardiidae | 0 | 500 | -5 | 30 | 5 | 40 |
| Common edible cockle | *Cerastoderma edule* | 0 | 60 | 5 | 15 | 5 | 40 |
| Variegated scallop | *Chlamys varia** | 0 | 700 | -5 | 30 | 30 | 40 |
| Choro mussel | *Choromytilus chorus** | 0 | 100 |  |  |  |  |
| Gasar cupped oyster | *Crassostrea gasar** | 0 | 60 | -5 | 25 | 30 | 40 |
| Pacific cupped oyster | *Crassostrea gigas* | 0 | 40 | 1.8 | 35 | 10 | 35 |
| Slipper cupped oyster | *Crassostrea iredalei** | 0 | 60 | -5 | 25 | 30 | 40 |
| Indian backwater oyster | *Crassostrea madrasensis** | 0 | 60 | -5 | 25 | 30 | 40 |
| Mangrove cupped oyster | *Crassostrea rhizophorae** | 0 | 60 | -5 | 25 | 30 | 40 |
| Cupped oysters nei | *Crassostrea spp.* | 0 | 60 | -5 | 25 | 30 | 40 |
| American cupped oyster | *Crassostrea virginica* | 0 | 40 | -5 | 25 | 30 | 40 |
| Gastropods nei | Gastropoda | 0 | 5000 | -5 | 30 | 30 | 40 |
| Japanese abalone | *Haliotis discus** | 0 | 60 | 5 | 30 | 30 | 40 |
| Perlemoen abalone | *Haliotis midae** | 0 | 60 | 5 | 30 | 30 | 40 |
| Red abalone | *Haliotis rufescens** | 0 | 60 | 5 | 30 | 30 | 40 |
| Abalones nei | *Haliotis spp.* | 0 | 60 | 5 | 30 | 30 | 40 |
| Bear paw clam | *Hippopus hippopus* | 0 | 10 | 20 | 30 | 30 | 40 |
| Northern quahog | *Mercenaria mercenaria* | 0 | 40 | 5 | 25 | 30 | 40 |
| Japanese hard clam | *Meretrix lusoria** | 20 | 30 | 25 | 30 | 30 | 35 |
| Marine molluscs nei | Mollusca | 0 | 5000 | -5 | 30 | 30 | 40 |
| Sand gaper | *Mya arenaria* | 0 | 120 | 0 | 15 | 5 | 40 |
| Sea mussels nei | Mytilidae | 0 | 5000 | -5 | 30 | 5 | 40 |
| Chilean mussel | *Mytilus chilensis** | 0 | 5000 | -5 | 30 | 5 | 40 |
| Blue mussel | *Mytilus edulis* | 0 | 400 | -5 | 25 | 5 | 40 |
| Mediterranean mussel | *Mytilus galloprovincialis* | 10 | 70 | 5 | 15 | 15 | 20 |
| Australian mussel | *Mytilus planulatus* | 0 | 10 | -5 | 15 | 30 | 35 |
| River Plata mussel | *Mytilus platensis** | 0 | 5000 | -5 | 30 | 5 | 40 |
| Chilean flat oyster | *Ostrea chilensis* | 40 | 110 | 5 | 15 | 30 | 40 |
| European flat oyster | *Ostrea edulis* | 0 | 50 | 5 | 25 | 30 | 40 |
| Flat and cupped oysters nei | Ostreidae | 0 | 70 | 5 | 30 | 30 | 35 |
| Pacific geoduck | *Panopea generosa** | 0 | 30 | 0 | 15 | 30 | 35 |
| Yesso scallop | *Patinopecten yessoensis** | 0 | 110 | 0 | 15 | 30 | 35 |
| Great Mediterranean scallop | *Pecten jacobaeus* | 0 | 130 | 15 | 20 | 35 | 40 |
| Great Atlantic scallop | *Pecten maximus* | 0 | 90 | 5 | 15 | 30 | 40 |
| Scallops nei | Pectinidae | 0 | 5000 | -5 | 30 | 30 | 40 |
| New Zealand mussel | *Perna canaliculus* | 0 | 10 | 15 | 20 | 35 | 40 |
| South American rock mussel | *Perna perna** | 0 | 10 | 15 | 25 | 35 | 40 |
| Green mussel | *Perna viridis* | 0 | 10 | 20 | 25 | 35 | 40 |
| Sea snails | *Rapana spp* | 0 | 50 | 5 | 30 | 15 | 40 |
| Grooved carpet shell | *Ruditapes decussatus* | 0 | 30 | 10 | 15 | 35 | 40 |
| Japanese carpet shell | *Ruditapes philippinarum* | 0 | 60 | 10 | 25 | 30 | 35 |
| Hooded oyster | *Saccostrea cuccullata** | 0 | 140 | 15 | 30 | 35 | 40 |
| Butter clam | *Saxidomus giganteus** | 0 | 30 | 5 | 15 | 30 | 35 |
| Solen razor clams nei | *Solen spp.* | 0 | 130 | 5 | 30 | 30 | 40 |
| Pacific horse clam | *Tresus nuttallii** | 0 | 30 | 5 | 15 | 30 | 35 |
| Smooth giant clam | *Tridacna derasa** | 0 | 20 | 25 | 30 | 30 | 40 |
| Elongate giant clam | *Tridacna maxima* | 0 | 20 | 25 | 30 | 30 | 40 |
| Giant clams nei | *Tridacna spp.* | 0 | 20 | 25 | 30 | 30 | 40 |
| Fluted giant clam | *Tridacna squamosa* | 0 | 20 | 25 | 30 | 30 | 40 |
| Golden carpet shell | *Venerupis aurea** | 0 | 60 | 5 | 25 | 30 | 40 |
| Pullet carpet shell | *Venerupis pullastra** | 0 | 60 | 5 | 25 | 30 | 40 |
| Warty venus | *Venus verrucosa* | 0 | 40 | 5 | 25 | 30 | 40 |

#### Reclassified Species sensitivity

Table S7: The overall Species sensitivity score for the molluscs cultured by mariculture-producing nations in 2014(FAO 2016), was assigned using the average value from indices within each layer (0 to 1 = Very-low, 1 to 2 = Low, 2 to 3 = Moderate, 3 to 4 = High, 4 to 5 = Very-high). Sensitivity scores for depth (1 = > 100m, 2 = 75 to 100m, 3 = 50 to 75m, 4 = 25 to 50m, 5 = 0 to 25m), temperature (1 = > 20°C, 2 = 15 to 20°C, 3 = 10 to 15°C, 4 = 5 to 10°C, 5 = 0 to 5°C), and salinity range (1 = > 20pps, 2 = 15 to 20pps, 3 = 10 to 15pps, 4 = 5 to 10 pps, 5 = 0 to 5 pps) were calculated by assigning species with greater habitat range a lower sensitivity score. Data on the distribution of species in terms of depth, temperature, and salinity were collated from the Ocean Biogeographic Information System (Grassle 2000).

|  | **Species sensitivity indices** | | |
| --- | --- | --- | --- |
| **Nation** | **Depth** | **Temperature** | **Salinity** |
| Argentina | 1 | 1 | 1 |
| Australia | 4 | 1 | 4 |
| Bosnia and Herzegovina | 3 | 4 | 5 |
| Brazil | 4 | 2 | 4 |
| Bulgaria | 3 | 4 | 5 |
| Cambodia | 4 | 3 | 3 |
| Canada | 1 | 1 | 3 |
| Channel Islands | 1 | 1 | 2 |
| Chile | 1 | 2 | 3 |
| China | 1 | 1 | 3 |
| Cook Islands | 5 | 5 | 4 |
| Croatia | 2 | 3 | 4 |
| Cuba | 3 | 1 | 4 |
| Denmark | 1 | 1 | 1 |
| El Salvador | 3 | 1 | 2 |
| France | 1 | 1 | 1 |
| Germany | 1 | 1 | 1 |
| Greece | 3 | 4 | 5 |
| Iceland | 1 | 1 | 1 |
| India | 4 | 2 | 4 |
| Ireland | 1 | 1 | 2 |
| Italy | 3 | 4 | 5 |
| Japan | 1 | 1 | 1 |
| North Korea | 1 | 1 | 4 |
| South Korea | 1 | 2 | 3 |
| Malaysia | 4 | 2 | 3 |
| Mauritius | 1 | 3 | 5 |
| Mexico | 1 | 1 | 1 |
| Montenegro | 3 | 3 | 4 |
| Namibia | 1 | 1 | 2 |
| Netherlands | 1 | 1 | 2 |
| New Caledonia | 4 | 1 | 1 |
| New Zealand | 4 | 1 | 3 |
| Norway | 1 | 1 | 2 |
| Palau | 5 | 4 | 4 |
| Peru | 5 | 5 | 5 |
| Philippines | 4 | 2 | 4 |
| Portugal | 1 | 1 | 3 |
| Romania | 3 | 4 | 5 |
| Russia | 1 | 1 | 2 |
| Senegal | 1 | 1 | 1 |
| Singapore | 5 | 2 | 3 |
| Slovenia | 3 | 4 | 5 |
| South Africa | 3 | 1 | 3 |
| Spain | 1 | 1 | 3 |
| St. Pierre and Miquelon | 1 | 1 | 1 |
| Sweden | 1 | 1 | 1 |
| Taiwan | 1 | 2 | 4 |
| Thailand | 4 | 2 | 3 |
| Tonga | 5 | 5 | 4 |
| Ukraine | 3 | 4 | 5 |
| United Kingdom | 1 | 2 | 2 |
| United States of America | 1 | 1 | 3 |
| Venezuela | 5 | 4 | 5 |
| Viet Nam | 1 | 1 | 4 |

### Nutritional contribution index

Table S8: The nutritional contribution index was represented by the percentage of dietary protein contributed by molluscs as directed in (Allison, Perry et al. 2009, Cooley, Lucey et al. 2012). FAO food balance sheets reported protein consumption data for each nation for non-cephalopod molluscs (‘molluscs, other’) and total consumption (g/capita/day), for the year 2011 (FAO 2012). These values were then normalised and linearly reclassified to our 1 – 5 risk scale where high relative contribution by molluscs to overall protein determines Sensitivity.

| **Nation** | **Mollusc protein (g/capita/day)** | **Animal Products Protein (g/capita/day)** | **Percent (%) of protein** |
| --- | --- | --- | --- |
| Albania | 0.03 | 51.23 | 0.06 |
| Algeria | 0.00 | 25.03 | 0.00 |
| Angola | 0.00 | 17.32 | 0.00 |
| Antigua and Barbuda | 0.70 | 59.27 | 1.18 |
| Argentina | 0.06 | 64.64 | 0.09 |
| Armenia | 0.00 | 39.56 | 0.00 |
| Australia | 0.18 | 71.94 | 0.25 |
| Austria | 0.05 | 64.37 | 0.08 |
| Azerbaijan | 0.00 | 29.66 | 0.00 |
| Bahamas | 0.08 | 53.58 | 0.15 |
| Bangladesh | 0.00 | 9.79 | 0.00 |
| Barbados | 0.04 | 51.43 | 0.08 |
| Belarus | 0.00 | 51.11 | 0.00 |
| Belgium | 0.32 | 58.71 | 0.55 |
| Belize | 0.24 | 27.62 | 0.87 |
| Benin | 0.00 | 11.75 | 0.00 |
| Bermuda | 0.34 | 66.14 | 0.51 |
| Bolivia | 0.00 | 29.81 | 0.00 |
| Bosnia and Herzegovina | 0.04 | 31.28 | 0.13 |
| Botswana | 0.00 | 25.40 | 0.00 |
| Brazil | 0.01 | 50.99 | 0.02 |
| Brunei Darussalam | 0.28 | 46.71 | 0.60 |
| Bulgaria | 0.02 | 39.23 | 0.05 |
| Burkina Faso | 0.00 | 12.58 | 0.00 |
| Cote d'Ivoire | 0.00 | 14.24 | 0.00 |
| Cabo Verde | 0.05 | 34.87 | 0.14 |
| Cambodia | 0.04 | 17.82 | 0.22 |
| Cameroon | 0.00 | 13.64 | 0.00 |
| Canada | 0.23 | 57.84 | 0.40 |
| Central African Republic | 0.00 | 21.28 | 0.00 |
| Chad | 0.00 | 10.16 | 0.00 |
| Chile | 0.06 | 44.37 | 0.14 |
| China | 5.12 | 274.41 | 1.87 |
| Colombia | 0.00 | 32.39 | 0.00 |
| Congo | 0.01 | 25.29 | 0.04 |
| Costa Rica | 0.04 | 38.14 | 0.10 |
| Croatia | 0.10 | 45.91 | 0.22 |
| Cuba | 0.02 | 29.87 | 0.07 |
| Cyprus | 0.07 | 47.37 | 0.15 |
| Czech Republic | 0.02 | 52.22 | 0.04 |
| North Korea | 0.01 | 10.04 | 0.10 |
| Denmark | 0.08 | 68.33 | 0.12 |
| Djibouti | 0.00 | 12.13 | 0.00 |
| Dominica | 0.00 | 46.54 | 0.00 |
| Dominican Republic | 0.02 | 26.19 | 0.08 |
| Ecuador | 0.00 | 41.39 | 0.00 |
| Egypt | 0.00 | 24.46 | 0.00 |
| El Salvador | 0.01 | 22.89 | 0.04 |
| Estonia | 0.02 | 51.25 | 0.04 |
| Ethiopia | 0.00 | 7.72 | 0.00 |
| Fiji | 0.03 | 32.75 | 0.09 |
| Finland | 0.02 | 69.72 | 0.03 |
| France | 0.70 | 71.03 | 0.99 |
| French Polynesia | 0.26 | 66.10 | 0.39 |
| Gabon | 0.00 | 42.37 | 0.00 |
| Gambia | 0.00 | 17.16 | 0.00 |
| Georgia | 0.01 | 26.67 | 0.04 |
| Germany | 0.03 | 62.21 | 0.05 |
| Ghana | 0.00 | 17.30 | 0.00 |
| Greece | 0.10 | 61.90 | 0.16 |
| Grenada | 0.00 | 42.74 | 0.00 |
| Guatemala | 0.00 | 18.28 | 0.00 |
| Guinea | 0.00 | 8.65 | 0.00 |
| Guinea-Bissau | 0.00 | 8.79 | 0.00 |
| Haiti | 0.00 | 10.33 | 0.00 |
| Honduras | 0.01 | 25.38 | 0.04 |
| Hungary | 0.01 | 44.17 | 0.02 |
| Iceland | 0.07 | 96.19 | 0.07 |
| India | 0.00 | 12.13 | 0.00 |
| Indonesia | 0.01 | 17.44 | 0.06 |
| Iran | 0.00 | 23.25 | 0.00 |
| Iraq | 0.00 | 12.18 | 0.00 |
| Ireland | 0.12 | 59.59 | 0.20 |
| Israel | 0.02 | 72.24 | 0.03 |
| Italy | 0.35 | 59.90 | 0.58 |
| Jamaica | 0.09 | 36.75 | 0.24 |
| Japan | 0.61 | 49.06 | 1.24 |
| Jordan | 0.00 | 29.60 | 0.00 |
| Kazakhstan | 0.00 | 56.88 | 0.00 |
| Kenya | 0.00 | 17.15 | 0.00 |
| Kiribati | 0.00 | 37.53 | 0.00 |
| Kuwait | 0.10 | 51.54 | 0.19 |
| Kyrgyzstan | 0.00 | 36.33 | 0.00 |
| Lao | 0.00 | 13.18 | 0.00 |
| Latvia | 0.08 | 57.69 | 0.14 |
| Lebanon | 0.02 | 30.48 | 0.07 |
| Lesotho | 0.00 | 12.04 | 0.00 |
| Liberia | 0.00 | 7.38 | 0.00 |
| Lithuania | 0.03 | 75.55 | 0.04 |
| Luxembourg | 0.32 | 72.06 | 0.44 |
| Madagascar | 0.00 | 10.44 | 0.00 |
| Malawi | 0.00 | 5.87 | 0.00 |
| Malaysia | 0.22 | 43.11 | 0.51 |
| Maldives | 0.03 | 72.21 | 0.04 |
| Mali | 0.00 | 26.52 | 0.00 |
| Malta | 0.32 | 59.17 | 0.54 |
| Mauritania | 0.00 | 32.97 | 0.00 |
| Mauritius | 0.05 | 39.68 | 0.13 |
| Mexico | 0.11 | 40.27 | 0.27 |
| Mongolia | 0.00 | 44.77 | 0.00 |
| Montenegro | 0.06 | 58.54 | 0.10 |
| Morocco | 0.00 | 24.28 | 0.00 |
| Mozambique | 0.00 | 6.15 | 0.00 |
| Myanmar | 0.00 | 34.12 | 0.00 |
| Namibia | 0.02 | 23.95 | 0.08 |
| Nepal | 0.00 | 11.03 | 0.00 |
| Netherlands | 0.08 | 70.78 | 0.11 |
| New Caledonia | 0.33 | 53.14 | 0.62 |
| New Zealand | 0.07 | 65.83 | 0.11 |
| Nicaragua | 0.02 | 20.53 | 0.10 |
| Niger | 0.00 | 14.73 | 0.00 |
| Nigeria | 0.00 | 10.27 | 0.00 |
| Norway | 0.06 | 64.41 | 0.09 |
| Oman | 0.00 | 45.87 | 0.00 |
| Pakistan | 0.00 | 26.48 | 0.00 |
| Panama | 0.04 | 40.93 | 0.10 |
| Paraguay | 0.00 | 33.30 | 0.00 |
| Peru | 0.30 | 26.12 | 1.15 |
| Philippines | 0.24 | 24.95 | 0.96 |
| Poland | 0.00 | 52.56 | 0.00 |
| Portugal | 0.38 | 69.03 | 0.55 |
| South Korea | 0.75 | 44.71 | 1.68 |
| Republic of Moldova | 0.00 | 34.08 | 0.00 |
| Romania | 0.00 | 49.21 | 0.00 |
| Russian Federation | 0.03 | 54.11 | 0.06 |
| Rwanda | 0.00 | 5.28 | 0.00 |
| Saint Kitts and Nevis | 0.10 | 47.96 | 0.21 |
| Saint Lucia | 0.02 | 51.63 | 0.04 |
| Saint Vincent and the Grenadines | 0.04 | 50.12 | 0.08 |
| Samoa | 0.20 | 48.42 | 0.41 |
| Sao Tome and Principe | 0.00 | 16.57 | 0.00 |
| Saudi Arabia | 0.00 | 36.20 | 0.00 |
| Senegal | 0.06 | 16.12 | 0.37 |
| Serbia | 0.00 | 37.27 | 0.00 |
| Sierra Leone | 0.01 | 14.72 | 0.07 |
| Slovakia | 0.00 | 35.94 | 0.00 |
| Slovenia | 0.06 | 55.64 | 0.11 |
| Solomon Islands | 0.06 | 18.74 | 0.32 |
| South Africa | 0.02 | 34.29 | 0.06 |
| Spain | 0.65 | 65.15 | 1.00 |
| Sri Lanka | 0.01 | 16.33 | 0.06 |
| Sudan | 0.00 | 29.88 | 0.00 |
| Suriname | 0.00 | 27.81 | 0.00 |
| Swaziland | 0.00 | 15.41 | 0.00 |
| Sweden | 0.07 | 70.86 | 0.10 |
| Switzerland | 0.12 | 58.66 | 0.20 |
| Tajikistan | 0.00 | 11.49 | 0.00 |
| Thailand | 0.13 | 24.20 | 0.54 |
| Macedonia | 0.00 | 31.98 | 0.00 |
| Timor-Leste | 0.00 | 16.42 | 0.00 |
| Togo | 0.00 | 7.27 | 0.00 |
| Trinidad and Tobago | 0.02 | 38.08 | 0.05 |
| Tunisia | 0.01 | 25.77 | 0.04 |
| Turkey | 0.03 | 32.79 | 0.09 |
| Turkmenistan | 0.00 | 38.66 | 0.00 |
| Uganda | 0.00 | 12.18 | 0.00 |
| Ukraine | 0.04 | 40.25 | 0.10 |
| United Arab Emirates | 0.03 | 37.70 | 0.08 |
| United Kingdom | 0.17 | 58.42 | 0.29 |
| United Republic of Tanzania | 0.00 | 10.05 | 0.00 |
| United States of America | 0.21 | 70.70 | 0.30 |
| Uruguay | 0.04 | 47.67 | 0.08 |
| Uzbekistan | 0.00 | 28.58 | 0.00 |
| Vanuatu | 0.03 | 28.18 | 0.11 |
| Venezuela | 0.16 | 45.76 | 0.35 |
| Viet Nam | 0.12 | 31.79 | 0.38 |
| Yemen | 0.00 | 12.42 | 0.00 |
| Zambia | 0.00 | 9.47 | 0.00 |
| Zimbabwe | 0.00 | 12.85 | 0.00 |

### Economic contribution

Table S9: The economic contribution index was calculated as the total molluscan mariculture production value (US$, FOA 2016) expressed as a percent of GDP (World Bank development indicators, World Bank, 2003) as directed in Allison, Perry et al. (2009) and Cooley, Kite-Powell et al. (2009). A mean of production values from 2012 – 2014 was calculated in order to minimise any inaccuracies in reporting (Handisyde, Ross et al. 2006). Mean (2012 - 2014) production values as a percent of GDP were normalised as in Allison, Perry et al. (2009), and linearly reclassified on to 1 – 5 risk scale, where relative economic importance determines Sensitivity.

| **Nation** | **GDP (million $USD)** | **Mollusc value (million $USD)** | **Percent of GDP** |
| --- | --- | --- | --- |
| Afghanistan | 20211 | 0.0 | 0E+00 |
| Albania | 12793 | 0.0 | 0E+00 |
| Algeria | 210756 | 0.0 | 0E+00 |
| Andorra | 3198 | 0.0 | 0E+00 |
| Angola | 122362 | 0.0 | 0E+00 |
| Antigua and Barbuda | 1209 | 0.0 | 0E+00 |
| Argentina | 544272 | 0.1 | 3E-05 |
| Armenia | 11128 | 0.0 | 0E+00 |
| Australia | 1518701 | 120.8 | 8E-03 |
| Austria | 424320 | 0.0 | 0E+00 |
| Azerbaijan | 72496 | 0.0 | 0E+00 |
| Bahamas, The | 8392 | 0.0 | 0E+00 |
| Bahrain | 32502 | 0.0 | 0E+00 |
| Bangladesh | 152077 | 0.0 | 0E+00 |
| Barbados | 4316 | 0.0 | 0E+00 |
| Belarus | 70939 | 0.0 | 0E+00 |
| Belgium | 516807 | 0.0 | 0E+00 |
| Belize | 1639 | 0.0 | 0E+00 |
| Benin | 8934 | 0.0 | 0E+00 |
| Bermuda | 5556 | 0.0 | 0E+00 |
| Bhutan | 1860 | 0.0 | 0E+00 |
| Bolivia | 30247 | 0.0 | 0E+00 |
| Bosnia and Herzegovina | 17961 | 0.1 | 8E-04 |
| Botswana | 15127 | 0.0 | 0E+00 |
| Brazil | 2447826 | 33.4 | 1E-03 |
| Brunei Darussalam | 16723 | 0.0 | 0E+00 |
| Bulgaria | 55307 | 2.0 | 4E-03 |
| Burkina Faso | 11943 | 0.0 | 0E+00 |
| Burundi | 2760 | 0.0 | 0E+00 |
| Cabo Verde | 1820 | 0.0 | 0E+00 |
| Cambodia | 15422 | 2.9 | 2E-02 |
| Cameroon | 29363 | 0.0 | 0E+00 |
| Canada | 1815169 | 80.0 | 4E-03 |
| Channel Islands |  | 4.9 |  |
| Central African Republic | 1790 | 0.0 | 0E+00 |
| Chad | 13080 | 0.0 | 0E+00 |
| Chile | 267015 | 2057.7 | 8E-01 |
| China | 9761013 | 10550.4 | 1E-01 |
| Colombia | 376089 | 0.0 | 0E+00 |
| Comoros | 591 | 0.0 | 0E+00 |
| Congo | 44067 | 0.0 | 0E+00 |
| Cook Islands | 311 | 0.0 | 5E-03 |
| Costa Rica | 48030 | 0.0 | 0E+00 |
| Cote d'Ivoire | 30862 | 0.0 | 0E+00 |
| Croatia | 57131 | 1.9 | 3E-03 |
| Cuba | 75144 | 1.5 | 2E-03 |
| Cyprus | 24075 | 0.0 | 0E+00 |
| Czech Republic | 206680 | 0.0 | 0E+00 |
| Denmark | 336686 | 1.1 | 3E-04 |
| Djibouti | 1466 | 0.0 | 0E+00 |
| Dominica | 505 | 0.0 | 0E+00 |
| Dominican Republic | 61921 | 0.0 | 0E+00 |
| Ecuador | 94539 | 0.0 | 0E+00 |
| Egypt, Arab Rep. | 287955 | 0.0 | 0E+00 |
| El Salvador | 24406 | 0.0 | 1E-04 |
| Equatorial Guinea | 16892 | 0.0 | 0E+00 |
| Estonia | 24956 | 0.0 | 0E+00 |
| Ethiopia | 48857 | 0.0 | 0E+00 |
| Faroe Islands | 2485 | 0.0 | 0E+00 |
| Fiji | 4235 | 0.0 | 0E+00 |
| Finland | 266341 | 0.0 | 0E+00 |
| France | 2773040 | 704.1 | 3E-02 |
| Gabon | 17647 | 0.0 | 0E+00 |
| Gambia, The | 889 | 0.0 | 0E+00 |
| Georgia | 16165 | 0.0 | 0E+00 |
| Germany | 3717741 | 14.8 | 4E-04 |
| Ghana | 42787 | 0.0 | 0E+00 |
| Greece | 240252 | 8.4 | 4E-03 |
| Greenland | 2405 | 0.0 | 0E+00 |
| Grenada | 851 | 0.0 | 0E+00 |
| Guatemala | 54321 | 0.0 | 0E+00 |
| Guinea | 6174 | 0.0 | 0E+00 |
| Guinea-Bissau | 1044 | 0.0 | 0E+00 |
| Guyana | 2973 | 0.0 | 0E+00 |
| Haiti | 8373 | 0.0 | 0E+00 |
| Honduras | 18802 | 0.0 | 0E+00 |
| Hungary | 133308 | 0.0 | 0E+00 |
| Iceland | 15536 | 0.1 | 8E-04 |
| India | 1910202 | 29.1 | 2E-03 |
| Indonesia | 906960 | 0.0 | 0E+00 |
| Iran, Islamic Rep. | 508052 | 0.0 | 0E+00 |
| Iraq | 224669 | 0.0 | 0E+00 |
| Ireland | 237909 | 70.1 | 3E-02 |
| Isle of Man | 6594 | 0.0 | 0E+00 |
| Israel | 285899 | 0.0 | 0E+00 |
| Italy | 2113898 | 78.7 | 4E-03 |
| Jamaica | 14312 | 0.0 | 0E+00 |
| Japan | 5154090 | 677.7 | 1E-02 |
| Jordan | 33453 | 0.0 | 0E+00 |
| Kazakhstan | 229038 | 0.0 | 0E+00 |
| Kenya | 55635 | 0.0 | 0E+00 |
| Kiribati | 170 | 0.0 | 0E+00 |
| North Korea | 17396 | 39.2 | 2E-01 |
| South Korea | 1313249 | 464.6 | 4E-02 |
| Kosovo | 6986 | 0.0 | 0E+00 |
| Kuwait | 170615 | 0.0 | 0E+00 |
| Kyrgyz Republic | 7136 | 0.0 | 0E+00 |
| Lao PDR | 10756 | 0.0 | 0E+00 |
| Latvia | 29844 | 0.0 | 0E+00 |
| Lebanon | 44429 | 0.0 | 0E+00 |
| Lesotho | 2261 | 0.0 | 0E+00 |
| Liberia | 1898 | 0.0 | 0E+00 |
| Libya | 62851 | 0.0 | 0E+00 |
| Liechtenstein | 5488 | 0.0 | 0E+00 |
| Lithuania | 45875 | 0.0 | 0E+00 |
| Luxembourg | 60885 | 0.0 | 0E+00 |
| Macedonia, FYR | 10627 | 0.0 | 0E+00 |
| Madagascar | 10515 | 0.0 | 0E+00 |
| Malawi | 5865 | 0.0 | 0E+00 |
| Malaysia | 325297 | 25.2 | 8E-03 |
| Maldives | 2791 | 0.0 | 0E+00 |
| Mali | 13359 | 0.0 | 0E+00 |
| Malta | 9263 | 0.0 | 0E+00 |
| Marshall Islands | 187 | 0.0 | 0E+00 |
| Mauritania | 5440 | 0.0 | 0E+00 |
| Mauritius | 11997 | 0.0 | 3E-04 |
| Mexico | 1248759 | 5.8 | 5E-04 |
| Micronesia | 320 | 0.0 | 0E+00 |
| Moldova | 7751 | 0.0 | 0E+00 |
| Mongolia | 12367 | 0.0 | 0E+00 |
| Montenegro | 4380 | 0.3 | 8E-03 |
| Morocco | 105170 | 0.0 | 0E+00 |
| Mozambique | 15833 | 0.0 | 0E+00 |
| Myanmar | 65891 | 0.0 | 0E+00 |
| Namibia | 12858 | 2.3 | 2E-02 |
| Nepal | 19297 | 0.0 | 0E+00 |
| New Caledonia | 10234 | 0.3 | 3E-03 |
| Netherlands | 857478 | 128.2 | 1E-02 |
| New Zealand | 189150 | 350.7 | 2E-01 |
| Nicaragua | 11035 | 0.0 | 0E+00 |
| Niger | 7618 | 0.0 | 0E+00 |
| Nigeria | 514806 | 0.0 | 0E+00 |
| Norway | 510990 | 5.0 | 1E-03 |
| Oman | 78774 | 0.0 | 0E+00 |
| Pakistan | 233060 | 0.0 | 0E+00 |
| Palau | 231 | 0.0 | 7E-03 |
| Panama | 44659 | 0.0 | 0E+00 |
| Papua New Guinea | 15911 | 0.0 | 0E+00 |
| Paraguay | 28153 | 0.0 | 0E+00 |
| Peru | 199196 | 451.0 | 2E-01 |
| Philippines | 268932 | 9.7 | 4E-03 |
| Poland | 523090 | 0.0 | 0E+00 |
| Portugal | 224186 | 30.5 | 1E-02 |
| Puerto Rico | 102108 | 0.0 | 0E+00 |
| Qatar | 200762 | 0.0 | 0E+00 |
| Romania | 187513 | 0.0 | 2E-06 |
| Russian Federation | 2143915 | 8.3 | 4E-04 |
| Rwanda | 7551 | 0.0 | 0E+00 |
| Samoa | 800 | 0.0 | 0E+00 |
| Saudi Arabia | 744041 | 0.0 | 0E+00 |
| St. Pierre and Miquelon | 0 | 0.2 |  |
| Senegal | 14885 | 0.7 | 4E-03 |
| Serbia | 43491 | 0.0 | 0E+00 |
| Seychelles | 1323 | 0.0 | 0E+00 |
| Sierra Leone | 4606 | 0.0 | 0E+00 |
| Singapore | 298634 | 0.4 | 1E-04 |
| Slovak Republic | 97110 | 0.0 | 0E+00 |
| Slovenia | 47802 | 0.3 | 6E-04 |
| Solomon Islands | 1081 | 0.0 | 0E+00 |
| Somalia | 5530 | 0.0 | 0E+00 |
| South Africa | 371106 | 46.4 | 1E-02 |
| South Sudan | 12303 | 0.0 | 0E+00 |
| Spain | 1363517 | 135.2 | 1E-02 |
| Sri Lanka | 74252 | 0.0 | 0E+00 |
| St. Kitts and Nevis | 795 | 0.0 | 0E+00 |
| St. Lucia | 1350 | 0.0 | 0E+00 |
| St. Vincent and the Grenadines | 714 | 0.0 | 0E+00 |
| Sudan | 67661 | 0.0 | 0E+00 |
| Suriname | 5107 | 0.0 | 0E+00 |
| Swaziland | 4629 | 0.0 | 0E+00 |
| Sweden | 564574 | 1.4 | 3E-04 |
| Switzerland | 683788 | 0.0 | 0E+00 |
| Tajikistan | 8459 | 0.0 |  |
| Tanzania | 43817 | 0.0 | 0E+00 |
| Thailand | 407166 | 0.0 | 0E+00 |
| Timor-Leste | 1328 | 114.7 | 3E-02 |
| Togo | 4143 | 0.0 | 0E+00 |
| Tonga | 442 | 0.0 | 0E+00 |
| Trinidad and Tobago | 26904 | 0.0 | 3E-04 |
| Tunisia | 46301 | 0.0 | 0E+00 |
| Turkey | 803634 | 0.0 | 0E+00 |
| Turkmenistan | 39282 | 0.0 | 0E+00 |
| Tuvalu | 39 | 0.0 | 0E+00 |
| Uganda | 24966 | 0.0 | 0E+00 |
| Ukraine | 162974 | 0.0 | 0E+00 |
| United Arab Emirates | 386691 | 0.1 | 6E-05 |
| United Kingdom | 2777657 | 0.0 | 0E+00 |
| United States | 16722162 | 67.1 | 2E-03 |
| Uruguay | 55344 | 277.3 | 2E-03 |
| Uzbekistan | 57037 | 0.0 | 0E+00 |
| Vanuatu | 799 | 0.0 | 0E+00 |
| Venezuela | 376311 | 0.0 | 0E+00 |
| Vietnam | 171082 | 0.0 | 2E-06 |
| West Bank and Gaza | 12157 | 168.1 | 1E-01 |
| Yemen | 34015 | 0.0 | 0E+00 |
| Zambia | 26894 | 0.0 | 0E+00 |
| Zimbabwe | 13360 | 0.0 | 0E+00 |

## Overall Sensitivity

Table S10: The Sensitivity sub-layer was generated by calculating a mean risk score for indicators for economic contribution, nutritional contribution, and species sensitivity for each nation. Missing data for indices were discounted. The overall Sensitivity risk score for each nation was categorised using the average for indices within the Sensitivity sub-layer (0 to 1 = Very-low, 1 to 2 = Low, 2 to 3 = Moderate, 3 to 4 = High, 4 to 5 = Very-high). Blank cells indicate nations where no data were available.

| **Nation** | **Economic contribution** | **Nutritional contribution** | **Species sensitivity** | **Sensitivity (S)** |
| --- | --- | --- | --- | --- |
| Afghanistan | 2 |  |  | 2 |
| Albania | 1 | 2 |  | 2 |
| Algeria | 1 | 1 |  | 1 |
| American Samoa | 1 |  |  | 1 |
| Andorra |  |  |  |  |
| Angola | 1 | 1 |  | 1 |
| Antigua and Barbuda |  | 5 |  | 5 |
| Argentina | 1 | 2 | 1 | 2 |
| Armenia | 1 | 1 |  | 1 |
| Australia | 3 | 3 | 3 | 3 |
| Austria | 1 | 2 |  | 2 |
| Azerbaijan | 1 | 1 |  | 1 |
| Bahamas |  | 2 |  | 2 |
| Bahrain | 1 |  |  | 1 |
| Bangladesh | 1 | 1 |  | 1 |
| Barbados | 1 | 2 |  | 2 |
| Belarus | 1 | 1 |  | 1 |
| Belgium | 1 | 4 |  | 3 |
| Belize | 1 | 4 |  | 3 |
| Benin | 1 | 1 |  | 1 |
| Bermuda |  | 4 |  | 4 |
| Bhutan | 1 |  |  | 1 |
| Bolivia | 1 | 1 |  | 1 |
| Bosnia and Herzegovina | 1 | 2 | 4 | 3 |
| Botswana | 1 |  |  | 1 |
| Brazil | 2 | 1 | 4 | 3 |
| Brunei Darussalam | 1 | 4 |  | 3 |
| Bulgaria | 3 | 2 | 4 | 3 |
| Burkina Faso | 1 | 1 |  | 1 |
| Burundi | 1 |  |  | 1 |
| Cabo Verde | 2 |  |  | 2 |
| Cambodia | 2 | 3 | 4 | 3 |
| Cameroon | 1 | 1 |  | 1 |
| Canada | 4 | 3 | 2 | 3 |
| Central African Republic | 1 | 1 |  | 1 |
| Chad | 1 | 1 |  | 1 |
| Channel Islands | 5 |  | 2 | 4 |
| Chile | 3 | 2 | 2 | 3 |
| China | 3 | 5 | 2 | 4 |
| Colombia | 1 | 1 |  | 1 |
| Comoros |  |  |  |  |
| Congo | 1 | 1 |  | 1 |
| Cook Islands | 5 |  | 5 | 5 |
| Costa Rica | 1 | 2 |  | 2 |
| Croatia | 2 | 3 | 3 | 3 |
| Cuba | 2 | 2 | 3 | 3 |
| Cyprus | 1 | 2 |  | 2 |
| Czech Republic | 1 | 1 |  | 1 |
| Denmark | 2 | 2 | 1 | 2 |
| Djibouti |  | 1 |  | 1 |
| Dominica | 1 | 1 |  | 1 |
| Dominican Republic | 1 | 2 |  | 2 |
| Ecuador | 1 | 1 |  | 1 |
| Egypt | 1 | 1 |  | 1 |
| El Salvador | 1 | 1 | 2 | 2 |
| Equatorial Guinea | 1 |  |  | 1 |
| Estonia | 1 | 1 |  | 1 |
| Ethiopia | 1 | 1 |  | 1 |
| Faroe Islands | 1 |  |  | 1 |
| Fiji | 1 | 2 |  | 2 |
| Finland | 1 | 1 |  | 1 |
| France | 5 | 4 | 1 | 4 |
| French Guiana | 1 |  |  | 1 |
| French Polynesia | 1 | 3 |  | 2 |
| Gabon | 1 | 1 |  | 1 |
| Gambia | 1 | 1 |  | 1 |
| Georgia | 1 | 1 |  | 1 |
| Germany | 3 | 1 | 1 | 2 |
| Ghana | 1 | 1 |  | 1 |
| Greece | 3 | 2 | 4 | 3 |
| Grenada |  | 1 |  | 1 |
| Guadeloupe | 1 |  |  | 1 |
| Guam | 1 |  |  | 1 |
| Guatemala | 1 | 1 |  | 1 |
| Guinea | 1 | 1 |  | 1 |
| Guinea-Bissau | 1 |  |  | 1 |
| Guyana | 1 |  |  | 1 |
| Haiti | 1 | 1 |  | 1 |
| Honduras | 1 | 1 |  | 1 |
| Hungary | 1 | 1 |  | 1 |
| Iceland | 1 | 2 | 1 | 2 |
| India | 1 | 1 | 4 | 2 |
| Indonesia | 1 | 2 |  | 2 |
| Iran | 1 | 1 |  | 1 |
| Iraq | 1 | 1 |  | 1 |
| Ireland | 5 | 3 | 2 | 4 |
| Israel | 1 | 1 |  | 1 |
| Italy | 4 | 4 | 4 | 4 |
| Ivory coast | 1 | 1 |  | 1 |
| Jamaica | 1 | 3 |  | 2 |
| Japan | 4 | 5 | 1 | 4 |
| Jordan | 1 | 1 |  | 1 |
| Kazakhstan | 1 | 1 |  | 1 |
| Kenya | 1 | 1 |  | 1 |
| Kiribati | 1 | 1 |  | 1 |
| North Korea | 3 | 2 | 2 | 3 |
| South Korea | 3 | 5 | 2 | 4 |
| Kosovo |  |  |  |  |
| Kuwait | 1 | 2 |  | 2 |
| Kyrgyzstan | 1 | 1 |  | 1 |
| Lao | 1 | 1 |  | 1 |
| Latvia | 1 | 2 |  | 2 |
| Lebanon | 1 | 2 |  | 2 |
| Lesotho | 1 | 1 |  | 1 |
| Liberia | 1 | 1 |  | 1 |
| Libya | 1 |  |  | 1 |
| Lithuania | 1 | 1 |  | 1 |
| Luxembourg | 3 |  |  | 3 |
| Macedonia | 1 | 1 |  | 1 |
| Madagascar | 1 | 1 |  | 1 |
| Malawi | 1 | 1 |  | 1 |
| Malaysia | 2 | 4 | 3 | 3 |
| Maldives |  | 1 |  | 1 |
| Mali | 1 | 1 |  | 1 |
| Malta | 1 | 4 |  | 3 |
| Marshall Islands | 1 |  |  | 1 |
| Martinique | 1 |  |  | 1 |
| Mauritania | 1 |  |  | 1 |
| Mauritius | 1 | 2 | 3 | 2 |
| Mayotte | 1 |  |  | 1 |
| Mexico | 2 | 3 | 1 | 2 |
| Moldova | 1 | 1 |  | 1 |
| Mongolia |  | 1 |  | 1 |
| Montenegro | 3 | 2 | 4 | 3 |
| Morocco | 1 | 1 |  | 1 |
| Mozambique | 1 | 1 |  | 1 |
| Myanmar | 1 | 1 |  | 1 |
| Namibia | 5 | 2 | 2 | 3 |
| Nauru | 1 |  |  | 1 |
| Nepal | 1 | 1 |  | 1 |
| Netherlands | 5 | 2 | 2 | 3 |
| New Caledonia | 1 | 4 | 2 | 3 |
| New Zealand | 5 | 2 | 3 | 4 |
| Nicaragua | 1 | 2 |  | 2 |
| Niger | 1 | 1 |  | 1 |
| Nigeria | 1 | 1 |  | 1 |
| Northern Mariana Is. | 1 |  |  | 1 |
| Norway | 1 | 2 | 2 | 2 |
| Oman | 1 | 1 |  | 1 |
| Pakistan | 1 | 1 |  | 1 |
| Palau | 3 |  | 5 | 4 |
| Palestine | 1 |  |  | 1 |
| Panama | 1 | 2 |  | 2 |
| Papua New Guinea | 1 |  |  | 1 |
| Paraguay | 1 | 1 |  | 1 |
| Peru | 4 | 5 | 5 | 5 |
| Philippines | 1 | 4 | 4 | 3 |
| Poland | 1 | 1 |  | 1 |
| Portugal | 4 | 4 | 2 | 4 |
| Puerto Rico | 1 |  |  | 1 |
| Qatar | 1 |  |  | 1 |
| Réunion | 1 |  |  | 1 |
| Romania | 1 | 1 | 4 | 2 |
| Russia | 1 | 2 | 2 | 2 |
| Rwanda | 1 | 1 |  | 1 |
| Saint Kitts and Nevis | 1 | 3 |  | 2 |
| Saint Lucia | 1 | 1 |  | 1 |
| Saint Pierre and Miquelon | 5 |  | 1 | 3 |
| Samoa | 1 | 3 |  | 2 |
| Sao Tome and Principe | 1 |  |  | 1 |
| Saudi Arabia | 1 | 1 |  | 1 |
| Senegal | 4 | 3 | 1 | 3 |
| Serbia | 1 | 1 |  | 1 |
| Sierra Leone | 1 | 2 |  | 2 |
| Singapore | 2 |  | 4 | 3 |
| Slovakia | 1 | 1 | 4 | 2 |
| Slovenia | 4 | 2 |  | 3 |
| Solomon Islands | 1 | 3 |  | 2 |
| Somalia |  |  |  |  |
| South Africa | 4 | 2 | 3 | 3 |
| South Sudan | 1 |  |  | 1 |
| Spain | 5 | 4 | 2 | 4 |
| Sri Lanka | 1 | 2 |  | 2 |
| St. Vincent and the Grenadines | 2 |  |  | 2 |
| Sudan | 1 | 1 |  | 1 |
| Suriname | 1 | 1 |  | 1 |
| Swaziland | 1 | 1 |  | 1 |
| Sweden | 3 | 2 | 1 | 2 |
| Switzerland | 1 | 3 |  | 2 |
| Syria | 1 |  |  | 1 |
| Taiwan | 2 |  | 3 | 3 |
| Tajikistan | 1 | 1 |  | 1 |
| Tanzania | 1 | 1 |  | 1 |
| Thailand | 3 | 4 | 3 | 4 |
| Timor-Leste | 1 | 1 |  | 1 |
| Togo | 1 | 1 |  | 1 |
| Tonga | 1 |  | 5 | 3 |
| Trinidad and Tobago | 1 | 2 |  | 2 |
| Tunisia | 1 | 1 |  | 1 |
| Turkey | 1 | 2 |  | 2 |
| Turkmenistan | 1 | 1 |  | 1 |
| Tuvalu | 1 |  |  | 1 |
| Uganda | 1 | 1 |  | 1 |
| Ukraine | 1 | 2 | 4 | 3 |
| United Arab Emirates | 1 | 2 |  | 2 |
| United Kingdom | 3 | 3 | 2 | 3 |
| United States | 4 | 3 | 2 | 3 |
| Uruguay | 1 | 2 |  | 2 |
| US Virgin Islands | 1 |  |  | 1 |
| Uzbekistan | 1 | 1 |  | 1 |
| Vanuatu | 1 | 2 |  | 2 |
| Venezuela | 1 | 3 | 5 | 3 |
| Vietnam | 2 | 3 | 2 | 3 |
| West Bank and Gaza | 1 |  |  | 1 |
| Yemen | 1 | 1 |  | 1 |
| Zambia | 1 | 1 |  | 1 |
| Zimbabwe | 1 | 1 |  | 1 |

# Adaptive capacity

### Governance

Table S11: The Governance index was defined by the World Bank Worldwide Governance Indicator (WGI), which combines six dimensions of governance (Kaufmann, Kraay et al. 2011) consisting of voice and accountability, political stability and absence of violence/terrorism, government effectiveness, regulatory quality, rule of law and control of corruption (Allison, Perry et al. 2009, Kaufmann, Kraay et al. 2011). The World Bank overall Worldwide Governance Indicator (WGI) equally weights each of the six components that were reclassifed on to our 1-5 scale from 0 (weak) to 5 (strong) governance. The average governance was determined for the year 2015. The mean WGI score was then inverted to calculate an Adaptive Capacity score, where high WGI score gives a low score for Adaptive capacity (i.e. indicates a stable functional state).

| **Nation** | **Voice and Accountability** | **Political Stability and Absence of Violence/ Terrorism** | **Government Effectiveness** | **Regulatory Quality** | **Rule of Law** | **Control of Corruption** | **Mean WGI** |
| --- | --- | --- | --- | --- | --- | --- | --- |
| Afghanistan | 1.3 | 0.0 | 1.2 | 1.5 | 0.9 | 1.2 | 1.0 |
| Albania | 2.7 | 2.9 | 2.5 | 2.7 | 2.1 | 2.1 | 2.5 |
| Algeria | 1.6 | 1.5 | 2.0 | 1.3 | 1.7 | 1.8 | 1.7 |
| Andorra | 3.7 | 3.9 | 4.3 | 3.4 | 4.1 | 3.7 | 3.9 |
| Angola | 1.3 | 1.9 | 1.5 | 1.6 | 1.4 | 1.1 | 1.5 |
| Antigua and Barbuda | 3.2 | 3.6 | 2.7 | 3.0 | 2.7 | 3.2 | 3.1 |
| Argentina | 2.8 | 2.4 | 2.4 | 1.5 | 1.7 | 1.9 | 2.1 |
| Armenia | 2.0 | 2.2 | 2.4 | 2.7 | 2.2 | 2.0 | 2.2 |
| Aruba | 3.7 | 3.8 | 3.4 | 3.9 | 3.8 | 3.8 | 3.7 |
| Australia | 3.9 | 3.4 | 4.1 | 4.3 | 4.3 | 4.4 | 4.1 |
| Austria | 3.9 | 3.7 | 4.0 | 3.9 | 4.4 | 4.0 | 4.0 |
| Azerbaijan | 1.0 | 1.8 | 2.3 | 2.2 | 1.9 | 1.7 | 1.8 |
| Bahamas | 3.6 | 3.5 | 3.2 | 2.8 | 3.1 | 3.8 | 3.3 |
| Bahrain | 1.2 | 1.4 | 3.1 | 3.3 | 3.0 | 2.7 | 2.4 |
| Bangladesh | 2.0 | 1.3 | 1.8 | 1.6 | 1.8 | 1.6 | 1.7 |
| Barbados | 3.7 | 3.8 | 3.5 | 3.0 | 3.6 | 4.3 | 3.7 |
| Belarus | 1.0 | 2.5 | 2.0 | 1.5 | 1.7 | 2.1 | 1.8 |
| Belgium | 3.9 | 3.1 | 3.9 | 3.8 | 3.9 | 4.1 | 3.8 |
| Belize | 3.1 | 2.5 | 1.8 | 2.0 | 1.8 | 2.3 | 2.2 |
| Benin | 2.8 | 2.5 | 1.9 | 1.9 | 1.9 | 1.9 | 2.2 |
| Bhutan | 2.4 | 3.6 | 2.9 | 1.8 | 3.0 | 3.5 | 2.9 |
| Bolivia | 2.4 | 2.2 | 1.8 | 1.6 | 1.3 | 1.8 | 1.9 |
| Bosnia and Herzegovina | 2.4 | 2.0 | 2.0 | 2.3 | 2.2 | 2.1 | 2.2 |
| Botswana | 2.9 | 3.5 | 3.0 | 3.0 | 3.1 | 3.3 | 3.2 |
| Brazil | 2.9 | 2.1 | 2.3 | 2.3 | 2.3 | 2.1 | 2.3 |
| Brunei Darussalam | 1.7 | 3.7 | 3.6 | 3.3 | 2.9 | 3.1 | 3.1 |
| Bulgaria | 2.9 | 2.5 | 2.7 | 3.1 | 2.4 | 2.2 | 2.6 |
| Burkina Faso | 2.3 | 1.8 | 1.9 | 2.1 | 2.0 | 2.2 | 2.1 |
| Burundi | 1.2 | 0.8 | 1.4 | 1.8 | 1.4 | 1.3 | 1.3 |
| Cambodia | 1.4 | 2.4 | 1.8 | 2.0 | 1.6 | 1.5 | 1.8 |
| Cameroon | 1.5 | 1.5 | 1.7 | 1.6 | 1.5 | 1.5 | 1.6 |
| Canada | 3.9 | 3.7 | 4.3 | 4.2 | 4.3 | 4.4 | 4.1 |
| Cape Verde | 3.5 | 3.3 | 2.7 | 2.2 | 3.1 | 3.4 | 3.0 |
| Cayman Islands | 2.9 | 3.7 | 3.6 | 3.3 | 3.3 | 3.5 | 3.4 |
| Central African Republic | 1.2 | 0.4 | 0.7 | 1.1 | 0.8 | 1.2 | 0.9 |
| Chad | 1.1 | 1.5 | 1.0 | 1.3 | 1.3 | 1.2 | 1.3 |
| Chile | 3.5 | 2.9 | 3.6 | 3.9 | 3.8 | 3.8 | 3.6 |
| China | 0.9 | 1.9 | 2.9 | 2.2 | 2.2 | 2.2 | 2.1 |
| Colombia | 2.4 | 1.4 | 2.5 | 3.0 | 2.2 | 2.2 | 2.3 |
| Comoros | 2.2 | 2.4 | 1.0 | 1.5 | 1.7 | 1.9 | 1.8 |
| Congo, Dem. Rep. | 1.2 | 0.3 | 0.9 | 1.2 | 0.9 | 1.2 | 1.0 |
| Congo | 1.5 | 2.0 | 1.5 | 1.3 | 1.5 | 1.3 | 1.5 |
| Costa Rica | 3.6 | 3.1 | 2.9 | 3.0 | 3.0 | 3.2 | 3.1 |
| Côte D'ivoire | 2.1 | 1.6 | 1.8 | 2.0 | 1.9 | 2.1 | 1.9 |
| Croatia | 3.0 | 3.1 | 3.0 | 2.9 | 2.7 | 2.7 | 2.9 |
| Cuba | 1.0 | 3.1 | 2.5 | 1.3 | 1.9 | 2.6 | 2.0 |
| Cyprus | 3.5 | 3.0 | 3.5 | 3.6 | 3.5 | 3.5 | 3.4 |
| Czech Republic | 3.5 | 3.5 | 3.6 | 3.6 | 3.6 | 2.9 | 3.4 |
| Denmark | 4.1 | 3.4 | 4.3 | 4.2 | 4.5 | 4.7 | 4.2 |
| Djibouti | 1.1 | 2.1 | 1.5 | 1.8 | 1.6 | 1.9 | 1.7 |
| Dominica | 3.6 | 3.7 | 2.6 | 2.8 | 3.1 | 3.1 | 3.2 |
| Dominican Republic | 2.6 | 2.7 | 2.2 | 2.5 | 2.0 | 1.7 | 2.3 |
| Ecuador | 2.2 | 2.4 | 2.1 | 1.4 | 1.5 | 1.8 | 1.9 |
| Egypt | 1.4 | 1.2 | 1.7 | 1.7 | 2.0 | 1.9 | 1.7 |
| El Salvador | 2.6 | 2.4 | 2.3 | 2.7 | 1.9 | 2.1 | 2.3 |
| Equatorial Guinea | 0.5 | 2.3 | 1.1 | 1.2 | 1.1 | 0.7 | 1.1 |
| Eritrea | 0.5 | 1.6 | 0.9 | 0.4 | 1.1 | 1.2 | 0.9 |
| Estonia | 3.7 | 3.1 | 3.6 | 4.2 | 3.8 | 3.8 | 3.7 |
| Ethiopia | 1.2 | 1.0 | 1.9 | 1.5 | 2.1 | 2.1 | 1.6 |
| Fiji | 2.6 | 3.1 | 2.2 | 2.1 | 2.1 | 2.4 | 2.4 |
| Finland | 4.1 | 3.5 | 4.3 | 4.3 | 4.6 | 4.8 | 4.3 |
| France | 3.7 | 2.8 | 3.9 | 3.7 | 3.9 | 3.8 | 3.6 |
| French Guiana | 3.7 | 3.0 | 3.6 | 3.9 | 3.8 | 3.5 | 3.6 |
| Gabon | 1.6 | 2.5 | 1.8 | 1.7 | 2.0 | 1.8 | 1.9 |
| Gambia | 1.0 | 2.5 | 1.6 | 2.0 | 1.8 | 1.7 | 1.8 |
| Georgia | 2.7 | 2.1 | 2.9 | 3.4 | 2.8 | 3.1 | 2.8 |
| Germany | 3.9 | 3.2 | 4.2 | 4.2 | 4.3 | 4.3 | 4.0 |
| Ghana | 3.0 | 2.5 | 2.2 | 2.5 | 2.6 | 2.3 | 2.5 |
| Greece | 3.1 | 2.3 | 2.7 | 2.9 | 2.7 | 2.4 | 2.7 |
| Greenland | 3.7 | 4.4 | 3.3 | 3.9 | 4.2 | 3.7 | 3.9 |
| Grenada | 3.4 | 3.3 | 2.3 | 2.5 | 2.3 | 2.8 | 2.8 |
| Guatemala | 2.1 | 1.9 | 1.8 | 2.3 | 1.5 | 1.8 | 1.9 |
| Guinea | 1.6 | 2.1 | 1.4 | 1.6 | 1.3 | 1.5 | 1.6 |
| Guinea-Bissau | 1.7 | 2.1 | 0.9 | 1.3 | 1.2 | 1.1 | 1.4 |
| Guyana | 2.7 | 2.3 | 2.2 | 1.9 | 2.0 | 1.7 | 2.1 |
| Haiti | 1.7 | 1.8 | 0.5 | 1.3 | 1.3 | 1.2 | 1.3 |
| Honduras | 2.1 | 2.0 | 1.7 | 2.1 | 1.5 | 1.9 | 1.9 |
| Hong Kong | 3.0 | 3.5 | 4.4 | 4.7 | 4.3 | 4.2 | 4.0 |
| Hungary | 3.0 | 3.2 | 3.0 | 3.3 | 2.9 | 2.6 | 3.0 |
| Iceland | 3.9 | 3.8 | 4.0 | 3.8 | 4.2 | 4.5 | 4.0 |
| India | 2.9 | 1.6 | 2.6 | 2.1 | 2.4 | 2.1 | 2.3 |
| Indonesia | 2.6 | 1.9 | 2.3 | 2.3 | 2.1 | 2.0 | 2.2 |
| Iran | 1.0 | 1.6 | 2.3 | 1.2 | 1.5 | 1.9 | 1.6 |
| Iraq | 1.3 | 0.2 | 1.2 | 1.3 | 1.0 | 1.1 | 1.0 |
| Ireland | 3.9 | 3.4 | 4.0 | 4.3 | 4.3 | 4.1 | 4.0 |
| Israel | 3.2 | 1.4 | 3.9 | 3.8 | 3.7 | 3.4 | 3.2 |
| Italy | 3.5 | 2.8 | 3.0 | 3.2 | 2.8 | 2.5 | 3.0 |
| Jamaica | 3.0 | 2.6 | 2.7 | 2.6 | 2.3 | 2.2 | 2.6 |
| Japan | 3.5 | 3.5 | 4.3 | 3.7 | 4.0 | 4.1 | 3.8 |
| Jersey | 3.7 | 4.0 | 3.8 | 3.7 | 4.2 | 3.7 | 3.9 |
| Jordan | 1.7 | 1.9 | 2.6 | 2.5 | 3.0 | 2.8 | 2.4 |
| Kazakhstan | 1.3 | 2.4 | 2.4 | 2.5 | 2.1 | 1.7 | 2.1 |
| Kenya | 2.3 | 1.2 | 2.2 | 2.2 | 2.0 | 1.5 | 1.9 |
| Kiribati | 3.5 | 3.4 | 2.0 | 1.6 | 2.3 | 2.7 | 2.6 |
| North Korea | 0.4 | 1.3 | 0.9 | 0.2 | 0.9 | 1.2 | 0.8 |
| South Korea | 3.2 | 2.6 | 3.5 | 3.7 | 3.5 | 3.0 | 3.2 |
| Kosovo | 2.3 | 2.2 | 2.1 | 2.2 | 2.0 | 2.0 | 2.1 |
| Kuwait | 1.8 | 2.4 | 2.5 | 2.3 | 2.5 | 2.3 | 2.3 |
| Kyrgyz Republic | 2.0 | 1.6 | 1.6 | 2.0 | 1.5 | 1.4 | 1.7 |
| Lao Pdr | 0.8 | 3.0 | 2.0 | 1.7 | 1.7 | 1.7 | 1.8 |
| Latvia | 3.3 | 3.0 | 3.6 | 3.6 | 3.3 | 2.9 | 3.3 |
| Lebanon | 2.0 | 0.8 | 2.0 | 2.2 | 1.7 | 1.6 | 1.7 |
| Lesotho | 2.6 | 2.4 | 1.8 | 2.1 | 2.3 | 2.6 | 2.3 |
| Liberia | 2.2 | 1.8 | 1.1 | 1.6 | 1.6 | 1.9 | 1.7 |
| Libya | 1.1 | 0.3 | 0.8 | 0.3 | 0.8 | 0.8 | 0.7 |
| Liechtenstein | 3.9 | 4.0 | 4.2 | 3.9 | 4.2 | 4.6 | 4.1 |
| Lithuania | 3.5 | 3.2 | 3.7 | 3.8 | 3.5 | 3.1 | 3.4 |
| Luxembourg | 4.0 | 3.9 | 4.2 | 4.2 | 4.4 | 4.6 | 4.2 |
| Macao | 2.4 | 3.7 | 3.9 | 4.2 | 3.4 | 3.1 | 3.5 |
| Macedonia | 2.3 | 2.3 | 2.6 | 2.9 | 2.3 | 2.4 | 2.5 |
| Madagascar | 2.1 | 2.1 | 1.2 | 1.7 | 1.8 | 1.7 | 1.8 |
| Malawi | 2.5 | 2.4 | 1.8 | 1.7 | 2.2 | 1.7 | 2.1 |
| Malaysia | 2.2 | 2.7 | 3.5 | 3.3 | 3.1 | 2.8 | 2.9 |
| Maldives | 2.0 | 3.0 | 2.1 | 2.1 | 2.0 | 2.2 | 2.2 |
| Mali | 2.3 | 0.8 | 1.6 | 1.9 | 1.7 | 1.9 | 1.7 |
| Malta | 3.7 | 3.5 | 3.4 | 3.7 | 3.7 | 3.4 | 3.6 |
| Marshall Islands | 3.7 | 3.5 | 0.9 | 1.4 | 2.3 | 2.4 | 2.4 |
| Mauritania | 1.6 | 1.8 | 1.5 | 1.6 | 1.7 | 1.6 | 1.6 |
| Mauritius | 3.3 | 3.4 | 3.5 | 3.6 | 3.4 | 2.9 | 3.4 |
| Mexico | 2.4 | 1.6 | 2.7 | 2.9 | 2.0 | 1.8 | 2.2 |
| Micronesia | 3.7 | 3.6 | 2.1 | 1.5 | 2.2 | 3.2 | 2.7 |
| Moldova | 2.5 | 2.1 | 1.9 | 2.4 | 2.1 | 1.6 | 2.1 |
| Mongolia | 2.7 | 3.1 | 2.1 | 2.2 | 2.1 | 2.0 | 2.4 |
| Montenegro | 2.7 | 2.6 | 2.7 | 2.7 | 2.5 | 2.4 | 2.6 |
| Morocco | 1.8 | 2.2 | 2.4 | 2.3 | 2.4 | 2.3 | 2.2 |
| Mozambique | 2.2 | 1.9 | 1.8 | 2.0 | 1.6 | 1.7 | 1.9 |
| Myanmar | 1.2 | 1.3 | 1.3 | 1.2 | 1.3 | 1.6 | 1.3 |
| Namibia | 3.1 | 3.1 | 2.8 | 2.4 | 2.7 | 2.8 | 2.8 |
| Nauru | 3.1 | 3.1 | 1.9 | 1.3 | 1.3 | 2.0 | 2.1 |
| Nepal | 2.1 | 1.6 | 1.5 | 1.7 | 1.8 | 2.0 | 1.8 |
| Netherlands | 4.1 | 3.4 | 4.3 | 4.3 | 4.4 | 4.4 | 4.2 |
| New Zealand | 4.1 | 4.0 | 4.4 | 4.4 | 4.5 | 4.8 | 4.4 |
| Nicaragua | 2.1 | 2.5 | 1.7 | 2.1 | 1.8 | 1.6 | 2.0 |
| Niger | 2.2 | 1.5 | 1.9 | 1.8 | 1.9 | 1.9 | 1.9 |
| Nigeria | 2.1 | 0.4 | 1.5 | 1.7 | 1.5 | 1.4 | 1.4 |
| Norway | 4.2 | 3.6 | 4.4 | 4.1 | 4.5 | 4.8 | 4.3 |
| Oman | 1.5 | 3.2 | 2.6 | 3.1 | 3.0 | 2.7 | 2.7 |
| Pakistan | 1.7 | 0.0 | 1.8 | 1.9 | 1.7 | 1.7 | 1.5 |
| Palau | 3.7 | 3.5 | 1.9 | 1.7 | 2.7 | 2.0 | 2.6 |
| Panama | 3.0 | 2.9 | 2.8 | 2.9 | 2.4 | 2.2 | 2.7 |
| Papua New Guinea | 2.6 | 1.9 | 1.9 | 1.9 | 1.6 | 1.5 | 1.9 |
| Paraguay | 2.4 | 2.5 | 1.6 | 2.2 | 1.8 | 1.6 | 2.0 |
| Peru | 2.7 | 2.0 | 2.2 | 3.0 | 2.0 | 1.9 | 2.3 |
| Philippines | 2.6 | 1.7 | 2.6 | 2.5 | 2.2 | 2.1 | 2.3 |
| Poland | 3.5 | 3.4 | 3.3 | 3.5 | 3.3 | 3.1 | 3.3 |
| Portugal | 3.6 | 3.4 | 3.7 | 3.4 | 3.6 | 3.4 | 3.5 |
| Puerto Rico | 2.9 | 3.3 | 2.9 | 3.5 | 3.3 | 2.6 | 3.1 |
| Qatar | 1.5 | 3.5 | 3.5 | 3.2 | 3.4 | 3.5 | 3.1 |
| Romania | 2.9 | 2.7 | 2.5 | 3.1 | 2.7 | 2.5 | 2.7 |
| Russia | 1.4 | 1.5 | 2.3 | 2.0 | 1.8 | 1.6 | 1.8 |
| Rwanda | 1.4 | 2.4 | 2.5 | 2.7 | 2.6 | 3.2 | 2.5 |
| Samoa | 3.2 | 3.7 | 3.0 | 2.4 | 3.2 | 2.8 | 3.1 |
| São Tomé and Principe | 2.8 | 2.7 | 1.7 | 1.7 | 1.7 | 2.3 | 2.2 |
| Saudi Arabia | 0.7 | 2.0 | 2.7 | 2.5 | 2.8 | 2.6 | 2.2 |
| Senegal | 2.8 | 2.3 | 2.1 | 2.3 | 2.3 | 2.5 | 2.4 |
| Serbia | 2.7 | 2.7 | 2.6 | 2.6 | 2.4 | 2.3 | 2.6 |
| Seychelles | 2.6 | 3.2 | 2.9 | 2.4 | 2.7 | 3.4 | 2.9 |
| Sierra Leone | 2.2 | 2.4 | 1.2 | 1.6 | 1.6 | 1.7 | 1.8 |
| Singapore | 2.4 | 3.7 | 4.8 | 4.8 | 4.4 | 4.6 | 4.1 |
| Slovak Republic | 3.5 | 3.5 | 3.3 | 3.3 | 3.0 | 2.6 | 3.2 |
| Slovenia | 3.4 | 3.4 | 3.5 | 3.1 | 3.5 | 3.2 | 3.4 |
| Solomon Islands | 2.8 | 3.2 | 1.5 | 1.5 | 2.0 | 2.1 | 2.2 |
| Somalia | 0.5 | 0.0 | 0.3 | 0.4 | 0.2 | 0.9 | 0.4 |
| South Africa | 3.1 | 2.3 | 2.8 | 2.8 | 2.6 | 2.5 | 2.7 |
| South Sudan | 1.0 | 0.1 | 0.3 | 0.8 | 0.7 | 0.8 | 0.6 |
| Spain | 3.5 | 2.8 | 3.7 | 3.3 | 3.4 | 3.0 | 3.3 |
| Sri Lanka | 2.1 | 2.5 | 2.5 | 2.4 | 2.6 | 2.1 | 2.4 |
| St. Kitts and Nevis | 3.5 | 3.2 | 2.6 | 2.7 | 2.8 | 2.8 | 2.9 |
| St. Lucia | 3.7 | 3.4 | 2.4 | 2.8 | 3.1 | 2.9 | 3.1 |
| St. Vincent and the Grenadines | 3.7 | 3.4 | 2.5 | 2.8 | 3.1 | 3.1 | 3.1 |
| Sudan | 0.7 | 0.3 | 1.0 | 1.0 | 1.3 | 1.0 | 0.9 |
| Suriname | 3.0 | 2.7 | 2.1 | 1.8 | 2.3 | 1.9 | 2.3 |
| Swaziland | 1.2 | 2.0 | 2.0 | 2.0 | 2.2 | 2.2 | 1.9 |
| Sweden | 4.1 | 3.5 | 4.3 | 4.3 | 4.5 | 4.7 | 4.2 |
| Switzerland | 4.1 | 3.8 | 4.5 | 4.3 | 4.5 | 4.7 | 4.3 |
| Syria | 0.7 | -0.4 | 0.9 | 0.9 | 1.1 | 1.0 | 0.7 |
| Taiwan | 3.4 | 3.3 | 3.9 | 3.8 | 3.7 | 3.3 | 3.6 |
| Tajikistan | 1.0 | 1.6 | 1.7 | 1.5 | 1.5 | 1.5 | 1.5 |
| Tanzania | 2.3 | 2.1 | 1.9 | 2.1 | 2.1 | 1.8 | 2.0 |
| Thailand | 1.6 | 1.5 | 2.9 | 2.8 | 2.4 | 2.1 | 2.2 |
| Timor-Leste | 2.6 | 2.3 | 1.5 | 1.5 | 1.3 | 1.8 | 1.8 |
| Togo | 1.8 | 2.3 | 1.3 | 1.7 | 1.7 | 1.8 | 1.8 |
| Tonga | 3.2 | 3.4 | 2.2 | 2.1 | 2.3 | 2.1 | 2.5 |
| Trinidad and Tobago | 3.0 | 2.8 | 2.7 | 2.6 | 2.4 | 2.0 | 2.6 |
| Tunisia | 2.7 | 1.6 | 2.4 | 2.1 | 2.4 | 2.4 | 2.3 |
| Turkey | 2.1 | 1.2 | 2.7 | 2.8 | 2.4 | 2.4 | 2.3 |
| Turkmenistan | 0.3 | 2.4 | 1.6 | 0.4 | 1.1 | 1.2 | 1.2 |
| Tuvalu | 3.6 | 3.9 | 1.5 | 1.8 | 2.8 | 2.5 | 2.7 |
| Uganda | 1.9 | 1.6 | 2.0 | 2.3 | 2.2 | 1.4 | 1.9 |
| Ukraine | 2.5 | 0.6 | 2.0 | 1.9 | 1.7 | 1.5 | 1.7 |
| United Arab Emirates | 1.4 | 3.3 | 4.0 | 3.6 | 3.2 | 3.6 | 3.2 |
| United Kingdom | 3.8 | 3.1 | 4.2 | 4.4 | 4.3 | 4.4 | 4.0 |
| United States of America | 3.6 | 3.2 | 4.0 | 3.8 | 4.1 | 3.9 | 3.8 |
| Uruguay | 3.6 | 3.5 | 3.0 | 3.0 | 3.2 | 3.8 | 3.3 |
| Uzbekistan | 0.6 | 2.1 | 1.8 | 0.8 | 1.4 | 1.3 | 1.3 |
| Vanuatu | 3.2 | 3.1 | 1.7 | 2.1 | 2.5 | 2.3 | 2.5 |
| Venezuela | 1.4 | 1.5 | 1.3 | 0.6 | 0.5 | 1.2 | 1.1 |
| Vietnam | 1.2 | 2.5 | 2.6 | 2.0 | 2.2 | 2.1 | 2.1 |
| West Bank and Gaza | 1.6 | 0.4 | 2.0 | 2.6 | 1.9 | 1.8 | 1.7 |
| Yemen | 1.0 | -0.1 | 0.9 | 1.4 | 1.3 | 1.1 | 0.9 |
| Zambia | 2.4 | 2.6 | 1.9 | 2.1 | 2.2 | 2.1 | 2.2 |
| Zimbabwe | 1.3 | 1.9 | 1.4 | 0.9 | 1.2 | 1.2 | 1.3 |

### Diversity

Table S12: The diversity of molluscs cultured in nations around the world, as calculated by the Shannon Index, which balances the richness (species count) and evenness (production tonnage per species in 2014 (FAO 2016) within a national mollusc sector to measure diversity (Shannon and Weaver 1949). The Shannon Index was calculated by the equation: $H^{'}= - \sum_{i=1}^{R} p_{i}lnp_{i}$, where *p_i_* is the proportion of production (t) belonging to the *i*th species farmed in 2014 for a given country (Shannon and Weaver 1949). The mollusc mariculture diversity index (H’) increases when the number of species increases and/or evenness of production between species increases; values of zero represent a monoculture.

| Nation | Species | Production (t) 2014 | Proportion (i) | Shannon (H’) |
| --- | --- | --- | --- | --- |
| Argentina | Pacific cupped oyster *Crassostrea gigas* | 7 | 0.5 |  |
|  | Blue mussel *Mytilus edulis* | 4 | 0.3 |  |
|  | River Plata mussel *Mytilus platensis* | 2 | 0.2 |  |
|  |  | **13** |  | **1.0** |
| Australia | Flat and cupped oysters Ostreidae | 11403 | 0.7 |  |
|  | Australian mussel *Mytilus planulatus* | 3237 | 0.2 |  |
|  | Abalones *Haliotis spp.* | 859 | 0.1 |  |
|  |  | **15499** |  | **0.7** |
| Bosnia and Herzegovina | Mediterranean mussel *Mytilus galloprovincialis* | 40 | 1.0 |  |
|  |  | **40** |  | **0.0** |
| Brazil | South American rock mussel *Perna perna* | 19292 | 0.9 |  |
|  | Cupped oysters *Crassostrea spp.* | 2800 | 0.1 |  |
|  |  | **22092** |  | **0.4** |
| Bulgaria | Mediterranean mussel *Mytilus galloprovincialis* | 2520 | 1.0 |  |
|  |  | **2520** |  | **0.0** |
| Cambodia | Green mussel *Perna viridis* | 2300 | 0.6 |  |
|  | Blood cockle *Tegillarca granosa* | 1300 | 0.4 |  |
|  |  | **3600** |  | **0.7** |
| Canada | Blue mussel *Mytilus edulis* | 25464 | 0.6 |  |
|  | Pacific cupped oyster *Crassostrea gigas* | 8108 | 0.2 |  |
|  | American cupped oyster *Crassostrea virginica* | 4496 | 0.1 |  |
|  | Japanese carpet shell *Ruditapes philippinarum* | 1327 | 0.0 |  |
|  | Butter clam *Saxidomus giganteus* | 299 | 0.0 |  |
|  | Marine molluscs Mollusca | 119 | 0.0 |  |
|  | Scallops Pectinidae | 114 | 0.0 |  |
|  |  | **39927** |  | **1.0** |
| Channel Islands | Pacific cupped oyster *Crassostrea gigas* | 1275 | 1.0 |  |
|  | Blue mussel *Mytilus edulis* | 39 | 0.0 |  |
|  | Great Atlantic scallop *Pecten maximus* | 2 | 0.0 |  |
|  | European flat oyster *Ostrea edulis* | 1 | 0.0 |  |
|  |  | **1317** |  | **0.2** |
| Chile | Chilean mussel *Mytilus chilensis* | 238088 | 1.0 |  |
|  | Peruvian calico scallop *Argopecten purpuratus* | 4146 | 0.0 |  |
|  | Choro mussel *Choromytilus chorus* | 1561 | 0.0 |  |
|  | Cholga mussel *Aulacomya ater* | 1172 | 0.0 |  |
|  | Red abalone *Haliotis rufescens* | 1130 | 0.0 |  |
|  | Chilean flat oyster *Ostrea chilensis* | 225 | 0.0 |  |
|  | Pacific cupped oyster *Crassostrea gigas* | 83 | 0.0 |  |
|  | Japanese abalone *Haliotis discus* | 16 | 0.0 |  |
|  |  | **246421** |  | **0.2** |
| China | Cupped oysters *Crassostrea spp* | 4352694 | 0.3 |  |
|  | Japanese carpet shell *Ruditapes philippinarum* | 3967033 | 0.3 |  |
|  | Scallops Pectinidae | 1649399 | 0.1 |  |
|  | Marine molluscs Mollusca | 885443 | 0.1 |  |
|  | Sea mussels Mytilidae | 805583 | 0.1 |  |
|  | Constricted tagelus *Sinonovacula constricta* | 786828 | 0.1 |  |
|  | Blood cockle *Tegillarca granosa* | 353388 | 0.0 |  |
|  | Sea snails *Rapana spp* | 232849 | 0.0 |  |
|  | Abalones *Haliotis spp* | 115458 | 0.0 |  |
|  | Pacific cupped oyster *Crassostrea gigas* | 25276 | 0.0 |  |
|  | Pen shells *Atrina spp.* | 17618 | 0.0 |  |
|  | Japanese hard clam *Meretrix lusoria* | 871 | 0.0 |  |
|  | Marine molluscs Mollusca | 55 | 0.0 |  |
|  | Diphos sanguin *Soletellina diphos* | 24 | 0.0 |  |
|  |  | **13192519** |  | **1.7** |
| Cook Islands | Giant clams *Tridacna spp* | 6 | 1.0 |  |
|  |  | **6** |  | **0.0** |
| Croatia | Mediterranean mussel *Mytilus galloprovincialis* | 714 | 1.0 |  |
|  | European flat oyster *Ostrea edulis* | 32 | 0.0 |  |
|  | Great Mediterranean scallop *Pecten jacobaeus* | 0 | 0.0 |  |
|  |  | **746** |  | **0.2** |
| Cuba | Mangrove cupped oyster *Crassostrea rhizophorae* | 1500 | 1.0 |  |
|  |  | **1500** |  | **0.0** |
| Denmark | Blue mussel *Mytilus edulis* | 1810 | 1.0 |  |
|  |  | **1810** |  | **0.0** |
| El Salvador | Cupped oysters *Crassostrea spp* | 10 | 0.6 |  |
|  | Black ark *Anadara sp.* | 5 | 0.3 |  |
|  | Grand ark *Anadara grandis* | 2 | 0.1 |  |
|  |  | **17** |  | **0.9** |
| France | Pacific cupped oyster *Crassostrea gigas* | 75100 | 0.5 |  |
|  | Blue mussel *Mytilus edulis* | 61000 | 0.4 |  |
|  | Mediterranean mussel *Mytilus galloprovincialis* | 14100 | 0.1 |  |
|  | Common edible cockle *Cerastoderma edule* | 1510 | 0.0 |  |
|  | European flat oyster *Ostrea edulis* | 1510 | 0.0 |  |
|  | Japanese carpet shell *Ruditapes philip pinarum* | 1000 | 0.0 |  |
|  | Clams, etc. Bivalvia | 250 | 0.0 |  |
|  |  | **154470** |  | **1.1** |
| Germany | Blue mussel *Mytilus edulis* | 5280 | 1.0 |  |
|  | Pacific cupped oyster *Crassostrea gigas* | 80 | 0.0 |  |
|  |  | **5360** |  | **0.1** |
| Greece | Mediterranean mussel *Mytilus galloprovincialis* | 16678 | 1.0 |  |
|  |  | **16678** |  | **0.0** |
| Iceland | Blue mussel *Mytilus edulis* | 144 | 1.0 |  |
|  |  | **144** |  | **0.0** |
| India | Green mussel *Perna viridis* | 9500 | 0.7 |  |
|  | Indian backwater oyster *Crassostrea madrasensis* | 4700 | 0.3 |  |
|  |  | **14200** |  | **0.6** |
| Ireland | Blue mussel *Mytilus edulis* | 11375 | 0.5 |  |
|  | Pacific cupped oyster *Crassostrea gigas* | 8887 | 0.4 |  |
|  | European flat oyster *Ostrea edulis* | 555 | 0.0 |  |
|  | Great Atlantic scallop *Pecten maximus* | 26 | 0.0 |  |
|  |  | **20843** |  | **0.8** |
| Italy | Mediterranean mussel *Mytilus galloprovincialis* | 79000 | 1.0 |  |
|  |  | **79000** |  | **0.0** |
| Japan | Yesso scallop *Patinopecten yessoensis* | 192300 | 0.5 |  |
|  | Pacific cupped oyster *Crassostrea gigas* | 184100 | 0.5 |  |
|  | Clams, etc. Bivalvia | 400 | 0.0 |  |
|  |  | **376800** |  | **0.7** |
| North Korea | Marine molluscs Mollusca | 60000 | 1.0 |  |
|  | Yesso scallop *Patinopecten yessoensis* | 200 | 0.0 |  |
|  |  | **60200** |  | **0.0** |
| South Korea | Pacific cupped oyster *Crassostrea gigas* | 283232 | 0.8 |  |
|  | Korean mussel *Mytilus coruscus* | 51463 | 0.1 |  |
|  | Abalones *Haliotis spp* | 8977 | 0.0 |  |
|  | Japanese carpet shell *Ruditapes philippinarum* | 7300 | 0.0 |  |
|  | Inflated ark Arcidae | 2921 | 0.0 |  |
|  | Yesso scallop *Patinopecten yessoensis* | 956 | 0.0 |  |
|  | Blood cockle *Tegillarca granosa* | 954 | 0.0 |  |
|  | Oriental cyclina *Cyclina sinensis* | 99 | 0.0 |  |
|  | Japanese hard clam *Meretrix lusoria* | 47 | 0.0 |  |
|  | Marine molluscs Mollusca | 33 | 0.0 |  |
|  |  | **355982** |  | **0.7** |
| Malaysia | Blood cockle *Tegillarca granosa* | 40454 | 0.9 |  |
|  | Green mussel *Perna viridis* | 1415 | 0.0 |  |
|  | Cupped oysters *Crassostrea spp* | 780 | 0.0 |  |
|  |  | **42649** |  | **0.2** |
| Mauritius | Hooded oyster *Saccostrea cuccullata* | 3 | 1.0 |  |
|  |  | **3** |  | **0.0** |
| Mexico | Pacific cupped oyster *Crassostrea gigas* | 3525 | 0.7 |  |
|  | Clams, etc. Bivalvia | 1025 | 0.2 |  |
|  | Sea mussels Mytilidae | 408 | 0.1 |  |
|  | Red abalone *Haliotis rufescens* | 22 | 0.0 |  |
|  | Gastropods Gastropoda | 15 | 0.0 |  |
|  | Abalones *Haliotis spp* | 0 | 0.0 |  |
|  |  | **4995** |  | **0.8** |
| Montenegro | Mediterranean mussel *Mytilus galloprovincialis* | 178 | 1.0 |  |
|  | European flat oyster *Ostrea edulis* | 1 | 0.0 |  |
|  |  | **179** |  | **0.0** |
| Namibia | Pacific cupped oyster *Crassostrea gigas* | 670 | 1.0 |  |
|  | Perlemoen abalone *Haliotis midae* | 15 | 0.0 |  |
|  | European flat oyster *Ostrea edulis* | 9 | 0.0 |  |
|  | Blue mussel *Mytilus edulis* | 1 | 0.0 |  |
|  |  | **695** |  | **0.2** |
| Netherlands | Blue mussel *Mytilus edulis* | 54300 | 1.0 |  |
|  | Cupped oysters *Crassostrea spp* | 2300 | 0.0 |  |
|  | European flat oyster *Ostrea edulis* | 200 | 0.0 |  |
|  |  | **56800** |  | **0.2** |
| New Caledonia | Pacific cupped oyster *Crassostrea gigas* | 20 | 1.0 |  |
|  |  | **20** |  | **0.0** |
| New Zealand | New Zealand mussel *Perna canaliculus* | 97438 | 1.0 |  |
|  | Pacific cupped oyster *Crassostrea gigas* | 1509 | 0.0 |  |
|  | Abalones *Haliotis spp* | 87 | 0.0 |  |
|  |  | **99034** |  | **0.1** |
| Norway | Pacific cupped oyster *Crassostrea gigas* | 1983 | 1.0 |  |
|  | Scallops Pectinidae | 15 | 0.0 |  |
|  | Blue mussel *Mytilus edulis* | 13 | 0.0 |  |
|  | European flat oyster *Ostrea edulis* | 4 | 0.0 |  |
|  |  | **2016** |  | **0.1** |
| Palau | Elongate giant clam *Tridacna maxima* | 3 | 0.4 |  |
|  | Smooth giant clam *Tridacna derasa* | 3 | 0.4 |  |
|  | Fluted giant clam *Tridacna squamosa* | 1 | 0.2 |  |
|  | Bear paw clam *Hippopus hippopus* | 0 | 0.0 |  |
|  |  | **7** |  | **1.1** |
| Peru | Peruvian calico scallop *Argopecten purpuratus* | 55096 | 1.0 |  |
|  |  | **55096** |  | **0.0** |
| Philippines | Slipper cupped oyster *Crassostrea iredalei* | 22355 | 0.5 |  |
|  | Green mussel *Perna viridis* | 18762 | 0.5 |  |
|  |  | **41117** |  | **0.7** |
| Portugal | Grooved carpet shell *Ruditapes decussatus* | 2235 | 0.5 |  |
|  | Sea mussels Mytilidae | 1297 | 0.3 |  |
|  | Flat and cupped oysters Ostreidae | 570 | 0.1 |  |
|  | Common edible cockle *Cerastoderma edule* | 263 | 0.1 |  |
|  | Solen razor clams *Solen spp* | 4 | 0.0 |  |
|  | Pullet carpet shell *Venerupis pullastra* | 2 | 0.0 |  |
|  | Golden carpet shell *Venerupis aurea* | 1 | 0.0 |  |
|  | Marine molluscs Mollusca | 0 | 0.0 |  |
|  |  | **4371** |  | **1.1** |
| Romania | Mediterranean mussel *Mytilus galloprovincialis* | 2 | 1.0 |  |
|  |  | **2** |  | **0.0** |
| Russia | Yesso scallop *Patinopecten yessoensis* | 2400 | 0.7 |  |
|  | Sea mussels Mytilidae | 798 | 0.2 |  |
|  | Cupped oysters *Crassostrea spp* | 57 | 0.0 |  |
|  |  | **3255** |  | **0.6** |
| Senegal | Gasar cupped oyster *Crassostrea gasar* | 157 | 0.6 |  |
|  | Pacific cupped oyster *Crassostrea gigas* | 75 | 0.3 |  |
|  | Blue mussel *Mytilus edulis* | 14 | 0.1 |  |
|  |  | **246** |  | **0.8** |
| Singapore | Green mussel *Perna viridis* | 467 | 1.0 |  |
|  | Pacific cupped oyster *Crassostrea gigas* | 1 | 0.0 |  |
|  |  | **469** |  | **0.0** |
| Slovenia | Mediterranean mussel *Mytilus galloprovincialis* | 423 | 1.0 |  |
|  |  | **423** |  | **0.0** |
| South Africa | Perlemoen abalone *Haliotis midae* | 1150 | 0.5 |  |
|  | Mediterranean mussel *Mytilus galloprovincialis* | 860 | 0.4 |  |
|  | Pacific cupped oyster *Crassostrea gigas* | 245 | 0.1 |  |
|  |  | **2255** |  | **1.0** |
| Spain | Sea mussels Mytilidae | 220449 | 1.0 |  |
|  | Pacific cupped oyster *Crassostrea gigas* | 507 | 0.0 |  |
|  | European flat oyster *Ostrea edulis* | 419 | 0.0 |  |
|  | Japanese carpet shell *Ruditapes philippinarum* | 163 | 0.0 |  |
|  | Grooved carpet shell *Ruditapes decussatus* | 25 | 0.0 |  |
|  | Pullet carpet shell *Venerupis pullastra* | 22 | 0.0 |  |
|  | Common edible cockle *Cerastoderma edule* | 17 | 0.0 |  |
|  | Queen scallop *Aequipecten opercularis* | 6 | 0.0 |  |
|  | Gastropods Gastropoda | 1 | 0.0 |  |
|  | Variegated scallop *Chlamys varia* | 1 | 0.0 |  |
|  | Warty venus *Venus verrucosa* | 0 | 0.0 |  |
|  | Marine molluscs Mollusca | 0 | 0.0 |  |
|  | Solen razor clams *Solen spp* | 0 | 0.0 |  |
|  |  | **221610** |  | **0.0** |
| St. Pierre and Miquelon | Scallops Pectinidae | 56 | 0.9 |  |
|  | Sea mussels Mytilidae | 3 | 0.1 |  |
|  |  | **59** |  | **0.2** |
| Sweden | Blue mussel *Mytilus edulis* | 1746 | 1.0 |  |
|  |  | **1746** |  | **0.0** |
| Thailand | Green mussel *Perna viridis* | 127030 | 0.6 |  |
|  | Blood cockle *Tegillarca granosa* | 65350 | 0.3 |  |
|  | Cupped oysters *Crassostrea spp* | 17187 | 0.1 |  |
|  |  | **209567** |  | **0.9** |
| Tonga | Elongate giant clam *Tridacna maxima* | 0 | 1.0 |  |
|  | Fluted giant clam *Tridacna squamosa* | 0 | 0.0 |  |
|  |  | **0** |  | **0.1** |
| Ukraine | Mediterranean mussel *Mytilus galloprovincialis* | 70 | 1.0 |  |
|  |  | **70** |  | **0.0** |
| United Kingdom | Blue mussel *Mytilus edulis* | 22500 | 0.9 |  |
|  | Pacific cupped oyster *Crassostrea gigas* | 1250 | 0.1 |  |
|  | European flat oyster *Ostrea edulis* | 60 | 0.0 |  |
|  | Northern quahog *Mercenaria mercenaria* | 10 | 0.0 |  |
|  | Great Atlantic scallop *Pecten maximus* | 10 | 0.0 |  |
|  | Common edible cockle *Cerastoderma edule* | 5 | 0.0 |  |
|  | Japanese carpet shell *Ruditapes philippinarum* | 5 | 0.0 |  |
|  | Queen scallop *Aequipecten opercularis* | 1 | 0.0 |  |
|  |  | **23841** |  | **0.2** |
| United States of America | American cupped oyster *Crassostrea virginica* | 93697 | 0.6 |  |
|  | Pacific cupped oyster *Crassostrea gigas* | 29116 | 0.2 |  |
|  | Northern quahog *Mercenaria mercenaria* | 28403 | 0.2 |  |
|  | Japanese carpet shell *Ruditapes philippinarum* | 3374 | 0.0 |  |
|  | Cupped oysters *Crassostrea spp* | 2173 | 0.0 |  |
|  | Blue mussel *Mytilus edulis* | 1744 | 0.0 |  |
|  | Sand gaper *Mya arenaria* | 964 | 0.0 |  |
|  | Pacific geoduck *Panopea generosa* | 613 | 0.0 |  |
|  | Abalones *Haliotis spp* | 341 | 0.0 |  |
|  | Pacific littleneck clam *Leukoma staminea* | 24 | 0.0 |  |
|  | Butter clam *Saxidomus giganteus* | 13 | 0.0 |  |
|  | Cockles Cardiidae | 1 | 0.0 |  |
|  | Pacific horse clam *Tresus nuttallii* | 1 | 0.0 |  |
|  |  | **160464** |  | **1.2** |
| Venezuela | South American rock mussel *Perna perna* | 1 | 1.0 |  |
|  |  | **1** |  | **0.0** |
| Vietnam | Marine molluscs Mollusca | 188881 | 1.0 |  |
|  |  | **188881** |  | **0.0** |

## Overall Adaptive capacity

Table S13: Adaptive capacity to CC-OA for nations with current mollusc aquaculture operations, measured as nation-specific mean of indices within the Adaptive capacity sub-layer, where 1 = Very-low, 2 = Low, 3 = Moderate, 4 = High, 5 = Very-high). Blank cells indicate nations where no data were available.

| Nation | Governance | Industry diversity | Adaptive capacity (AC) |
| --- | --- | --- | --- |
| Afghanistan | 4 |  | 4 |
| Albania | 3 |  | 3 |
| Algeria | 4 |  | 4 |
| Andorra | 2 |  | 2 |
| Angola | 4 |  | 4 |
| Antigua and Barbuda | 2 |  | 2 |
| Argentina | 3 | 2 | 3 |
| Armenia | 3 |  | 3 |
| Aruba | 2 |  | 2 |
| Australia | 1 | 3 | 2 |
| Austria | 2 |  | 2 |
| Azerbaijan | 4 |  | 4 |
| Bahamas | 2 |  | 2 |
| Bahrain | 3 |  | 3 |
| Bangladesh | 4 |  | 4 |
| Barbados | 2 |  | 2 |
| Belarus | 4 |  | 4 |
| Belgium | 2 |  | 2 |
| Belize | 3 |  | 3 |
| Benin | 3 |  | 3 |
| Bhutan | 3 |  | 3 |
| Bolivia | 4 |  | 4 |
| Bosnia and Herzegovina | 3 | 5 | 4 |
| Botswana | 2 |  | 2 |
| Brazil | 3 | 4 | 4 |
| Brunei Darussalam | 2 |  | 2 |
| Bulgaria | 3 | 5 | 4 |
| Burkina Faso | 3 |  | 3 |
| Burundi | 4 |  | 4 |
| Cabo Verde |  |  | 3 |
| Cambodia | 4 | 4 | 4 |
| Cameroon | 4 |  | 4 |
| Canada | 1 | 3 | 2 |
| Cape Verde | 2 |  | 2 |
| Cayman Islands | 2 |  | 2 |
| Central African Republic | 5 |  | 5 |
| Chad | 4 |  | 4 |
| Channel Islands |  | 5 | 5 |
| Chile | 2 | 5 | 4 |
| China | 3 | 1 | 2 |
| Colombia | 3 |  | 3 |
| Comoros | 4 |  | 4 |
| Congo | 5 |  | 5 |
| Cook Islands |  | 5 | 5 |
| Costa Rica | 2 |  | 2 |
| Croatia | 3 | 5 | 4 |
| Cuba | 3 | 5 | 4 |
| Cyprus | 2 |  | 2 |
| Czech Republic | 2 |  | 2 |
| Democratic Republic of the Congo | 4 |  | 4 |
| Denmark | 1 | 5 | 3 |
| Djibouti | 4 |  | 4 |
| Dominica | 2 |  | 2 |
| Dominican Republic | 3 |  | 3 |
| Ecuador | 4 |  | 4 |
| Egypt | 4 |  | 4 |
| El Salvador | 3 | 3 | 3 |
| Equatorial Guinea | 4 |  | 4 |
| Eritrea | 5 |  | 5 |
| Estonia | 2 |  | 2 |
| Ethiopia | 4 |  | 4 |
| Fiji | 3 |  | 3 |
| Finland | 1 |  | 1 |
| France | 2 | 2 | 2 |
| French Guiana | 2 |  | 2 |
| Gabon | 4 |  | 4 |
| Gambia | 4 |  | 4 |
| Georgia | 3 |  | 3 |
| Germany | 1 | 5 | 3 |
| Ghana | 3 |  | 3 |
| Greece | 3 | 5 | 4 |
| Greenland | 2 |  | 2 |
| Grenada | 3 |  | 3 |
| Guatemala | 4 |  | 4 |
| Guinea | 4 |  | 4 |
| Guinea-Bissau | 4 |  | 4 |
| Guyana | 3 |  | 3 |
| Haiti | 4 |  | 4 |
| Honduras | 4 |  | 4 |
| Hong Kong | 1 |  | 1 |
| Hungary | 2 |  | 2 |
| Iceland | 1 | 5 | 3 |
| India | 3 | 4 | 4 |
| Indonesia | 3 |  | 3 |
| Iran | 4 |  | 4 |
| Iraq | 4 |  | 4 |
| Ireland | 1 | 3 | 2 |
| Israel | 2 |  | 2 |
| Italy | 3 | 5 | 4 |
| Ivory Coast | 4 |  | 4 |
| Jamaica | 3 |  | 3 |
| Japan | 2 | 3 | 3 |
| Jersey | 2 |  | 2 |
| Jordan | 3 |  | 3 |
| Kazakhstan | 3 |  | 3 |
| Kenya | 4 |  | 4 |
| Kiribati | 3 |  | 3 |
| Kosovo | 3 |  | 3 |
| Kuwait | 3 |  | 3 |
| Kyrgyzstan | 4 |  | 4 |
| Lao | 4 |  | 4 |
| Latvia | 2 |  | 2 |
| Lebanon | 4 |  | 4 |
| Lesotho | 3 |  | 3 |
| Liberia | 4 |  | 4 |
| Libya | 5 |  | 5 |
| Liechtenstein | 1 |  | 1 |
| Lithuania | 2 |  | 2 |
| Luxembourg | 1 |  | 1 |
| Macao | 2 |  | 2 |
| Macedonia | 3 |  | 3 |
| Madagascar | 4 |  | 4 |
| Malawi | 3 |  | 3 |
| Malaysia | 3 | 5 | 4 |
| Maldives | 3 |  | 3 |
| Mali | 4 |  | 4 |
| Malta | 2 |  | 2 |
| Marshall Islands | 3 |  | 3 |
| Mauritania | 4 |  | 4 |
| Mauritius | 2 | 5 | 4 |
| Mexico | 3 | 3 | 3 |
| Micronesia | 3 |  | 3 |
| Moldova | 3 |  | 3 |
| Mongolia | 3 |  | 3 |
| Montenegro | 3 | 5 | 4 |
| Morocco | 3 |  | 3 |
| Mozambique | 4 |  | 4 |
| Myanmar | 4 |  | 4 |
| Namibia | 3 | 5 | 4 |
| Nauru | 3 |  | 3 |
| Nepal | 4 |  | 4 |
| Netherlands | 1 | 5 | 3 |
| New Caledonia |  | 5 | 5 |
| New Zealand | 1 | 5 | 3 |
| Nicaragua | 4 |  | 4 |
| Niger | 4 |  | 4 |
| Nigeria | 4 |  | 4 |
| North Korea | 5 | 5 | 5 |
| Norway | 1 | 5 | 3 |
| Oman | 3 |  | 3 |
| Pakistan | 4 |  | 4 |
| Palau | 3 | 2 | 3 |
| Palestine | NA |  | 3 |
| Panama | 3 |  | 3 |
| Papua New Guinea | 4 |  | 4 |
| Paraguay | 3 |  | 3 |
| Peru | 3 | 5 | 4 |
| Philippines | 3 | 4 | 4 |
| Poland | 2 |  | 2 |
| Portugal | 2 | 2 | 2 |
| Puerto Rico | 2 |  | 2 |
| Qatar | 2 |  | 2 |
| Romania | 3 | 5 | 4 |
| Russia | 4 | 4 | 4 |
| Rwanda | 3 |  | 3 |
| Saint Kitts and Nevis | 3 |  | 3 |
| Saint Lucia | 2 |  | 2 |
| Saint Vincent and the Grenadines | 2 |  | 2 |
| Samoa | 2 |  | 2 |
| Sao Tome and Principe | 3 |  | 3 |
| Saudi Arabia | 3 |  | 3 |
| Senegal | 3 | 3 | 3 |
| Serbia | 3 |  | 3 |
| Seychelles | 3 |  | 3 |
| Sierra Leone | 4 |  | 4 |
| Singapore | 1 | 5 | 3 |
| Slovakia | 2 |  | 2 |
| Slovenia | 2 | 5 | 4 |
| Solomon Islands | 3 |  | 3 |
| Somalia | 5 |  | 5 |
| South Africa | 3 | 3 | 3 |
| South Korea | 2 | 3 | 3 |
| South Sudan | 5 |  | 5 |
| Spain | 2 | 5 | 4 |
| Sri Lanka | 3 |  | 3 |
| St. Pierre and Miquelon |  | 5 | 5 |
| Sudan | 5 |  | 5 |
| Suriname | 3 |  | 3 |
| Swaziland | 4 |  | 4 |
| Sweden | 1 | 5 | 3 |
| Switzerland | 1 |  | 1 |
| Syria | 5 |  | 5 |
| Taiwan | 2 |  | 2 |
| Tajikistan | 4 |  | 4 |
| Tanzania | 3 |  | 3 |
| Thailand | 3 | 3 | 3 |
| Timor-Leste | 4 |  | 4 |
| Togo | 4 |  | 4 |
| Tonga | 3 | 5 | 4 |
| Trinidad and Tobago | 3 |  | 3 |
| Tunisia | 3 |  | 3 |
| Turkey | 3 |  | 3 |
| Turkmenistan | 4 |  | 4 |
| Tuvalu | 3 |  | 3 |
| Uganda | 4 |  | 4 |
| Ukraine | 4 | 5 | 5 |
| United Arab Emirates | 2 |  | 2 |
| United Kingdom | 1 | 5 | 3 |
| United States | 2 | 2 | 2 |
| Uruguay | 2 |  | 2 |
| Uzbekistan | 4 |  | 4 |
| Vanuatu | 3 |  | 3 |
| Venezuela | 4 | 5 | 5 |
| Vietnam | 3 | 5 | 4 |
| West bank and Gaza | 4 |  | 4 |
| Yemen | 5 |  | 5 |
| Zambia | 3 |  | 4 |
| Zimbabwe | 4 |  | 4 |

# Vulnerability

Table S14: Overall Vulnerability to CC-OA for coastal nations, measured as nation-specific mean for Exposure, Sensitivity, and Adaptive capacity sub-layers for decades from 2020 to 2100, where 1 = Very-low, 2 = Low, 3 = Moderate, 4 = High, 5 = Very-high. Blank cells indicate the absence of data.

| Nation | 2020 | 2030 | 2040 | 2050 | 2060 | 2070 | 2080 | 2090 | 2100 |
| --- | --- | --- | --- | --- | --- | --- | --- | --- | --- |
| Albania | 3 | 3 | 3 | 3 | 3 | 3 | 3 | 4 | 3 |
| Algeria | 3 | 3 | 3 | 3 | 3 | 3 | 3 | 3 | 3 |
| American Samoa | 1 | 1 | 1 | 1 | 1 | 1 | 1 | 1 | 1 |
| Angola | 3 | 2 | 2 | 2 | 2 | 3 | 3 | 3 | 3 |
| Antarctica | 2 | 2 | 2 | 2 | 3 | 3 | 3 | 3 | 3 |
| Antigua and Barbuda | 4 | 4 | 4 | 3 | 4 | 4 | 4 | 4 | 4 |
| Argentina | 3 | 3 | 3 | 3 | 3 | 3 | 3 | 3 | 3 |
| Australia | 3 | 3 | 3 | 3 | 3 | 3 | 3 | 3 | 3 |
| Bahamas | 3 | 3 | 3 | 2 | 3 | 3 | 3 | 3 | 3 |
| Bahrain | 2 | 2 | 3 | 3 | 3 | 3 | 3 | 3 | 3 |
| Bangladesh | 3 | 3 | 3 | 3 | 3 | 3 | 3 | 3 | 3 |
| Barbados | 2 | 2 | 2 | 2 | 2 | 2 | 2 | 2 | 2 |
| Belgium | 3 | 3 | 3 | 3 | 3 | 3 | 3 | 3 | 3 |
| Belize | 3 | 3 | 3 | 3 | 3 | 3 | 3 | 4 | 3 |
| Benin | 2 | 2 | 2 | 2 | 2 | 3 | 3 | 3 | 3 |
| Bermuda | 4 | 4 | 4 | 4 | 4 | 4 | 4 | 4 | 4 |
| Bosnia and Herzegovina | 4 | 4 | 4 | 4 | 4 | 4 | 4 | 4 | 4 |
| Brazil | 3 | 3 | 3 | 3 | 3 | 3 | 3 | 3 | 4 |
| Bulgaria | 4 | 4 | 4 | 4 | 4 | 4 | 4 | 4 | 4 |
| Cambodia | 3 | 3 | 3 | 3 | 3 | 4 | 4 | 4 | 4 |
| Cameroon | 3 | 3 | 3 | 3 | 3 | 3 | 3 | 3 | 3 |
| Canada | 3 | 3 | 3 | 3 | 3 | 3 | 3 | 3 | 3 |
| Cape Verde | 2 | 2 | 2 | 2 | 2 | 2 | 3 | 3 | 2 |
| Chile | 3 | 3 | 3 | 3 | 3 | 3 | 3 | 4 | 4 |
| China | 4 | 3 | 3 | 3 | 3 | 4 | 4 | 4 | 4 |
| Colombia | 2 | 2 | 2 | 2 | 2 | 2 | 2 | 2 | 2 |
| Comoros | 4 | 4 | 4 | 4 | 4 | 4 | 4 | 4 | 4 |
| **Cook Islands** | **5** | **5** | **5** | **5** | **5** | **5** | **5** | **5** | **5** |
| Costa Rica | 2 | 2 | 2 | 2 | 2 | 2 | 2 | 3 | 3 |
| Croatia | 3 | 3 | 4 | 4 | 4 | 4 | 4 | 4 | 4 |
| Cuba | 4 | 4 | 4 | 4 | 4 | 4 | 4 | 4 | 4 |
| Cyprus | 2 | 2 | 2 | 2 | 2 | 3 | 2 | 3 | 3 |
| Democratic Republic of the Congo | 3 | 3 | 3 | 3 | 3 | 3 | 3 | 3 | 3 |
| Denmark | 2 | 2 | 2 | 2 | 2 | 2 | 2 | 3 | 3 |
| Djibouti | 3 | 3 | 3 | 3 | 3 | 3 | 3 | 3 | 3 |
| Dominica | 2 | 2 | 2 | 2 | 2 | 2 | 2 | 2 | 2 |
| Dominican Republic | 3 | 3 | 3 | 3 | 3 | 3 | 3 | 3 | 3 |
| Ecuador | 2 | 2 | 2 | 2 | 2 | 2 | 3 | 3 | 3 |
| Egypt | 3 | 3 | 3 | 3 | 3 | 3 | 3 | 3 | 3 |
| El Salvador | 3 | 3 | 2 | 2 | 2 | 3 | 3 | 3 | 3 |
| Equatorial Guinea | 2 | 3 | 3 | 3 | 3 | 3 | 3 | 3 | 3 |
| Eritrea | 3 | 3 | 4 | 4 | 4 | 4 | 4 | 4 | 4 |
| Estonia | 2 | 2 | 2 | 2 | 2 | 2 | 2 | 2 | 2 |
| Faroe Islands | 1 | 1 | 1 | 1 | 1 | 1 | 1 | 1 | 1 |
| Fiji | 3 | 3 | 3 | 3 | 3 | 3 | 3 | 3 | 3 |
| Finland | 1 | 1 | 1 | 1 | 1 | 1 | 1 | 1 | 1 |
| France | 3 | 3 | 3 | 3 | 3 | 3 | 3 | 3 | 3 |
| French Polynesia | 2 | 2 | 2 | 2 | 2 | 2 | 2 | 2 | 2 |
| Gabon | 2 | 2 | 3 | 3 | 3 | 3 | 3 | 3 | 3 |
| Gambia | 3 | 3 | 3 | 3 | 3 | 3 | 3 | 3 | 3 |
| Georgia | 3 | 3 | 3 | 3 | 3 | 3 | 3 | 3 | 3 |
| Germany | 3 | 3 | 3 | 3 | 3 | 3 | 3 | 3 | 3 |
| Ghana | 2 | 2 | 2 | 2 | 2 | 3 | 3 | 3 | 3 |
| Greece | 4 | 4 | 3 | 4 | 4 | 4 | 4 | 4 | 4 |
| Grenada | 2 | 2 | 2 | 2 | 2 | 2 | 2 | 3 | 3 |
| Guam | 1 | 1 | 1 | 1 | 1 | 1 | 1 | 1 | 1 |
| Guatemala | 3 | 2 | 2 | 3 | 2 | 3 | 3 | 3 | 3 |
| Guinea | 3 | 3 | 3 | 3 | 3 | 3 | 3 | 3 | 3 |
| Guinea-Bissau | 3 | 3 | 3 | 3 | 3 | 3 | 3 | 3 | 3 |
| Guyana | 2 | 2 | 2 | 2 | 2 | 2 | 2 | 2 | 2 |
| Haiti | 3 | 3 | 3 | 3 | 3 | 3 | 3 | 3 | 3 |
| Honduras | 3 | 3 | 3 | 3 | 3 | 3 | 3 | 3 | 3 |
| Hong Kong | 1 | 1 | 1 | 1 | 1 | 1 | 1 | 1 | 1 |
| Iceland | 2 | 2 | 2 | 2 | 2 | 3 | 3 | 3 | 3 |
| India | 3 | 3 | 3 | 3 | 3 | 3 | 3 | 3 | 3 |
| Indonesia | 3 | 3 | 3 | 3 | 3 | 3 | 3 | 3 | 3 |
| Iran | 3 | 3 | 3 | 3 | 3 | 3 | 3 | 3 | 3 |
| Iraq | 3 | 3 | 3 | 3 | 3 | 3 | 3 | 3 | 3 |
| Ireland | 3 | 3 | 3 | 3 | 3 | 3 | 3 | 3 | 4 |
| Israel | 2 | 2 | 2 | 2 | 2 | 2 | 2 | 2 | 2 |
| Italy | 4 | 4 | 4 | 4 | 4 | 4 | 4 | 4 | 4 |
| Ivory Coast | 3 | 3 | 3 | 3 | 3 | 3 | 3 | 4 | 4 |
| Jamaica | 3 | 3 | 3 | 3 | 3 | 3 | 3 | 3 | 3 |
| Japan | 4 | 4 | 4 | 4 | 4 | 4 | 4 | 4 | 4 |
| Jordan | 2 | 2 | 2 | 2 | 2 | 2 | 2 | 2 | 2 |
| Kenya | 2 | 2 | 3 | 3 | 3 | 3 | 3 | 3 | 3 |
| Kiribati | 3 | 3 | 3 | 3 | 3 | 3 | 3 | 3 | 3 |
| Kuwait | 3 | 3 | 3 | 3 | 3 | 3 | 3 | 3 | 3 |
| Latvia | 2 | 2 | 2 | 2 | 2 | 2 | 2 | 2 | 2 |
| Lebanon | 3 | 3 | 3 | 3 | 3 | 3 | 3 | 3 | 3 |
| Liberia | 3 | 3 | 3 | 3 | 3 | 3 | 3 | 3 | 3 |
| Libya | 3 | 3 | 3 | 3 | 3 | 4 | 3 | 4 | 4 |
| Lithuania | 2 | 2 | 2 | 2 | 2 | 2 | 2 | 2 | 2 |
| Madagascar | 3 | 3 | 3 | 3 | 3 | 3 | 3 | 3 | 3 |
| Malaysia | 3 | 3 | 3 | 3 | 3 | 3 | 3 | 3 | 3 |
| Maldives | 2 | 2 | 2 | 2 | 2 | 2 | 2 | 2 | 3 |
| Malta | 3 | 3 | 3 | 3 | 3 | 4 | 3 | 4 | 3 |
| Marshall Islands | 3 | 2 | 3 | 3 | 3 | 3 | 3 | 3 | 3 |
| Mauritania | 3 | 3 | 3 | 3 | 3 | 3 | 3 | 3 | 3 |
| Mauritius | 3 | 3 | 3 | 3 | 3 | 3 | 3 | 3 | 3 |
| Mexico | 3 | 3 | 3 | 3 | 3 | 3 | 3 | 3 | 3 |
| Micronesia | 3 | 3 | 3 | 3 | 3 | 4 | 4 | 4 | 4 |
| Montenegro | 4 | 4 | 4 | 4 | 4 | 4 | 4 | 4 | 4 |
| Morocco | 2 | 2 | 2 | 2 | 2 | 3 | 2 | 3 | 3 |
| Mozambique | 3 | 3 | 3 | 3 | 3 | 3 | 3 | 3 | 3 |
| Namibia | 3 | 4 | 4 | 4 | 4 | 4 | 4 | 4 | 4 |
| Nauru | 3 | 3 | 3 | 3 | 3 | 3 | 3 | 3 | 3 |
| Netherlands | 3 | 3 | 3 | 3 | 3 | 3 | 3 | 3 | 3 |
| New Caledonia | 4 | 4 | 4 | 4 | 4 | 4 | 4 | 4 | 4 |
| New Zealand | 3 | 3 | 3 | 3 | 3 | 3 | 4 | 4 | 4 |
| Nicaragua | 3 | 3 | 3 | 3 | 3 | 3 | 3 | 3 | 3 |
| Nigeria | 3 | 3 | 3 | 3 | 3 | 3 | 3 | 3 | 3 |
| North Korea | 4 | 4 | 4 | 4 | 4 | 4 | 4 | 4 | 4 |
| Norway | 3 | 3 | 3 | 3 | 3 | 3 | 3 | 3 | 3 |
| Oman | 2 | 2 | 2 | 2 | 2 | 2 | 3 | 3 | 3 |
| Pakistan | 2 | 2 | 2 | 2 | 2 | 3 | 3 | 3 | 3 |
| Palau | 4 | 4 | 4 | 4 | 4 | 4 | 4 | 4 | 4 |
| Panama | 3 | 3 | 3 | 3 | 3 | 3 | 3 | 3 | 3 |
| Papua New Guinea | 3 | 3 | 3 | 3 | 3 | 3 | 3 | 3 | 3 |
| Peru | 4 | 4 | 4 | 4 | 4 | 4 | 4 | 4 | 4 |
| Philippines | 4 | 4 | 4 | 4 | 4 | 4 | 4 | 4 | 4 |
| Poland | 2 | 2 | 2 | 2 | 2 | 2 | 2 | 2 | 3 |
| Portugal | 3 | 3 | 3 | 3 | 3 | 4 | 3 | 4 | 4 |
| Puerto Rico | 1 | 1 | 1 | 1 | 1 | 1 | 1 | 1 | 1 |
| Qatar | 2 | 2 | 2 | 2 | 2 | 3 | 3 | 2 | 3 |
| Congo | 1 | 2 | 1 | 2 | 2 | 2 | 2 | 2 | 2 |
| Romania | 3 | 3 | 3 | 3 | 3 | 4 | 4 | 4 | 4 |
| Russia | 4 | 4 | 4 | 4 | 4 | 4 | 4 | 4 | 4 |
| Saint Kitts and Nevis | 3 | 3 | 3 | 3 | 3 | 3 | 3 | 3 | 3 |
| Saint Lucia | 2 | 2 | 2 | 2 | 2 | 2 | 2 | 2 | 2 |
| Saint Pierre and Miquelon | 3 | 3 | 3 | 3 | 3 | 3 | 3 | 3 | 3 |
| Saint Vincent and the Grenadines | 2 | 2 | 2 | 2 | 2 | 2 | 3 | 3 | 3 |
| Samoa | 2 | 2 | 2 | 2 | 3 | 3 | 3 | 3 | 3 |
| Sao Tome and Principe | 2 | 2 | 2 | 2 | 2 | 2 | 2 | 2 | 3 |
| Saudi Arabia | 2 | 2 | 2 | 2 | 2 | 2 | 2 | 3 | 3 |
| Senegal | 3 | 3 | 3 | 3 | 3 | 3 | 3 | 3 | 3 |
| Seychelles | 2 | 2 | 3 | 3 | 3 | 3 | 3 | 3 | 3 |
| Sierra Leone | 3 | 3 | 3 | 3 | 3 | 3 | 3 | 3 | 3 |
| Singapore | 3 | 3 | 3 | 3 | 3 | 3 | 3 | 3 | 3 |
| Slovenia | 4 | 4 | 4 | 4 | 4 | 4 | 4 | 4 | 4 |
| Solomon Islands | 3 | 3 | 3 | 3 | 3 | 3 | 3 | 3 | 3 |
| **Somalia** | **5** | **5** | **5** | **5** | **5** | **5** | **5** | **5** | **5** |
| South Africa | 3 | 3 | 3 | 3 | 3 | 3 | 3 | 3 | 3 |
| South Korea | 4 | 4 | 4 | 3 | 4 | 4 | 4 | 4 | 4 |
| Spain | 4 | 4 | 4 | 4 | 4 | 4 | 4 | 4 | 4 |
| Sri Lanka | 3 | 3 | 3 | 3 | 3 | 3 | 3 | 3 | 3 |
| Sudan | 3 | 3 | 3 | 3 | 3 | 3 | 3 | 3 | 3 |
| Suriname | 2 | 2 | 2 | 2 | 2 | 2 | 2 | 2 | 2 |
| Sweden | 2 | 2 | 2 | 2 | 3 | 3 | 3 | 3 | 3 |
| Syria | 2 | 2 | 2 | 2 | 3 | 3 | 2 | 3 | 3 |
| Taiwan | 4 | 4 | 4 | 3 | 4 | 4 | 4 | 4 | 4 |
| Tanzania | 1 | 1 | 1 | 1 | 1 | 1 | 2 | 2 | 2 |
| Thailand | 3 | 3 | 3 | 3 | 3 | 3 | 4 | 4 | 4 |
| Timor-Leste | 3 | 3 | 3 | 3 | 3 | 3 | 3 | 3 | 3 |
| Togo | 3 | 3 | 3 | 3 | 3 | 3 | 3 | 3 | 3 |
| Tonga | 4 | 4 | 4 | 3 | 4 | 4 | 4 | 4 | 4 |
| Trinidad and Tobago | 3 | 3 | 3 | 3 | 3 | 3 | 3 | 3 | 3 |
| Tunisia | 3 | 3 | 3 | 3 | 3 | 3 | 3 | 3 | 3 |
| Turkey | 3 | 3 | 3 | 3 | 3 | 3 | 3 | 3 | 4 |
| Tuvalu | 3 | 2 | 2 | 3 | 3 | 3 | 3 | 3 | 3 |
| Ukraine | 4 | 4 | 4 | 4 | 4 | 4 | 4 | 4 | 4 |
| United Arab Emirates | 2 | 2 | 2 | 2 | 3 | 3 | 3 | 3 | 3 |
| United Kingdom | 3 | 3 | 3 | 3 | 3 | 3 | 3 | 3 | 3 |
| United States | 3 | 3 | 3 | 3 | 3 | 3 | 3 | 3 | 3 |
| Uruguay | 2 | 2 | 2 | 2 | 2 | 2 | 2 | 3 | 3 |
| Vanuatu | 3 | 3 | 3 | 3 | 3 | 3 | 3 | 3 | 3 |
| Venezuela | 2 | 2 | 2 | 2 | 2 | 2 | 2 | 3 | 3 |
| Vietnam | 3 | 3 | 3 | 3 | 4 | 4 | 4 | 4 | 4 |
| Western Sahara | 2 | 2 | 2 | 2 | 2 | 2 | 2 | 3 | 3 |
| Yemen | 3 | 4 | 4 | 4 | 4 | 4 | 4 | 4 | 4 |

## Tipping points

Table S15: Correlation (R) between MDS axis scores for indices within the exposure (E) sub layer for top-15 mollusc mariculture producing nations, from 2020 to 2100. Results of NA are for indices that do not change exposure score over time. Blank cells represent nations where data for an index are not available. Values in bold represent the index with the highest correlation with the MDS axes and highest explanatory power for calculation of tipping points for decades 2020 to 2100.

| **Nation** | **Sea surface temperature** | | **Aragonite saturation depth** | | **Primary productivity** | |
| --- | --- | --- | --- | --- | --- | --- |
|  | MDS1 | MDS2 | MDS1 | MDS2 | MDS1 | MDS2 |
| Chile | **0.74** | 0.13 | **0.74** | 0.13 | 0.48 | 0.27 |
| China | **0.94** | 0.00 | 0.20 | 0.52 | 0.85 | 0.08 |
| France | 0.67 | 0.03 | **0.92** | 0.08 | NA | NA |
| Italy | 0.25 | 0.03 | 0.36 | 0.03 | 0.11 | **0.44** |
| Japan | **0.34** | 0.04 | 0.27 | 0.05 | 0.19 | 0.04 |
| Netherlands | **0.39** | 0.00 | NA | NA | NA | NA |
| North Korea | **0.89** | 0.00 | NA | NA | 0.33 | 0.56 |
| New Zealand | 0.06 | 0.02 | 0.14 | 0.05 | NA | NA |
| Peru | **0.96** | 0.00 | NA | NA | 0.89 | 0.05 |
| South Korea | **0.96** | 0.00 | NA | NA | 0.89 | 0.04 |
| Spain | **0.96** | 0.03 | **0.96** | 0.03 | NA | NA |
| Taiwan | 0.86 | 0.01 | 0.41 | 0.20 | **0.94** | 0.01 |
| Thailand | **0.99** | 0.00 | NA | NA | 0.89 | 0.00 |
| United States of America | **0.99** | 0.00 | 0.10 | 0.00 | 0.81 | 0.00 |
| Vietnam | **0.82** | 0.01 | - | - | 0.68 | 0.00 |

# Data availability

Table S16: Summary of data availability for sub-layers (Exposure in 2100, Sensitivity, and Adaptive capacity) used to calculate overall Vulnerability of all nations in 2100 and data availability across the sub-models.

| **Nation** | **Exposure 2100** | **Sensitivity** | **Adaptive capacity** | **Vulnerability 2100** | **Data availability (%)** |
| --- | --- | --- | --- | --- | --- |
| Afghanistan |  | 2 | 4 | 3 | 67 |
| Albania | 4 | 2 | 3 | 3 | 100 |
| Algeria | 4 | 1 | 4 | 3 | 100 |
| American Samoa |  | 1 |  | 1 | 33 |
| Andorra | 2 |  | 2 | 2 | 67 |
| Angola | 3 | 1 | 4 | 3 | 100 |
| Antigua and Barbuda | 4 | 5 | 2 | 5 | 100 |
| Argentina | 3 | 2 | 3 | 3 | 100 |
| Armenia |  | 1 | 3 | 2 | 67 |
| Aruba |  |  | 2 | 2 | 33 |
| Australia | 3 | 3 | 2 | 3 | 100 |
| Austria |  | 2 | 2 | 2 | 67 |
| Azerbaijan |  | 1 | 4 | 3 | 67 |
| Bahamas | 5 | 2 | 2 | 3 | 100 |
| Bahrain | 4 | 1 | 3 | 3 | 100 |
| Bangladesh | 4 | 1 | 4 | 3 | 100 |
| Barbados | 2 | 2 | 2 | 2 | 100 |
| Belarus |  | 1 | 4 | 3 | 67 |
| Belgium |  | 3 | 2 | 3 | 67 |
| Belize | 3 | 3 | 3 | 3 | 100 |
| Benin | 4 | 1 | 3 | 3 | 100 |
| Bermuda |  | 4 |  | 4 | 33 |
| Bhutan |  | 1 | 3 | 2 | 67 |
| Bolivia |  | 1 | 4 | 3 | 67 |
| Bosnia and Herzegovina |  | 3 | 4 | 4 | 67 |
| Botswana |  | 1 | 2 | 2 | 67 |
| Brazil | 3 | 3 | 4 | 4 | 100 |
| Brunei Darussalam |  | 3 | 2 | 3 | 67 |
| Bulgaria | 4 | 3 | 4 | 4 | 100 |
| Burkina Faso |  | 1 | 3 | 2 | 67 |
| Burundi |  | 1 | 4 | 3 | 67 |
| Cabo Verde |  | 2 | 3 | 3 | 67 |
| Cambodia | 3 | 3 | 4 | 4 | 100 |
| Cameroon | 3 | 1 | 4 | 3 | 100 |
| Canada | 4 | 3 | 2 | 3 | 100 |
| Cape Verde | 2 |  | 2 | 2 | 67 |
| Cayman Islands |  |  | 2 | 2 | 33 |
| Central African Republic |  | 1 | 5 | 3 | 67 |
| Chad |  | 1 | 4 | 3 | 67 |
| Channel Islands |  | 4 | 5 | 5 | 67 |
| Chile | 3 | 3 | 4 | 4 | 100 |
| China | 4 | 4 | 2 | 4 | 100 |
| Colombia | 2 | 1 | 3 | 2 | 100 |
| Comoros |  |  | 4 | 4 | 33 |
| Congo |  | 1 | 5 | 3 | 67 |
| Cook Islands |  | 5 | 5 | 5 | 67 |
| Costa Rica | 3 | 2 | 2 | 3 | 100 |
| Croatia | 4 | 3 | 4 | 4 | 100 |
| Cuba | 3 | 3 | 4 | 4 | 100 |
| Cyprus | 3 | 2 | 2 | 3 | 100 |
| Czech Republic |  | 1 | 2 | 2 | 67 |
| Democratic Republic of the Congo | 2 |  | 4 | 3 | 67 |
| Denmark | 2 | 2 | 3 | 3 | 100 |
| Djibouti | 3 | 1 | 4 | 3 | 100 |
| Dominica | 3 | 1 | 2 | 2 | 100 |
| Dominican Republic | 4 | 2 | 3 | 3 | 100 |
| Ecuador | 2 | 1 | 4 | 3 | 100 |
| Egypt | 2 | 1 | 4 | 3 | 100 |
| El Salvador | 2 | 2 | 3 | 3 | 100 |
| Equatorial Guinea | 2 | 1 | 4 | 3 | 100 |
| Eritrea | 3 |  | 5 | 4 | 67 |
| Estonia |  | 1 | 2 | 2 | 67 |
| Ethiopia |  | 1 | 4 | 3 | 67 |
| Faroe Islands |  | 1 |  | 1 | 33 |
| Fiji | 4 | 2 | 3 | 3 | 100 |
| Finland |  | 1 | 1 | 1 | 67 |
| France | 3 | 4 | 2 | 3 | 100 |
| French Guiana |  | 1 | 2 | 2 | 67 |
| French Polynesia |  | 2 |  | 2 | 33 |
| Gabon | 2 | 1 | 4 | 3 | 100 |
| Gambia | 4 | 1 | 4 | 3 | 100 |
| Georgia | 5 | 1 | 3 | 3 | 100 |
| Germany | 4 | 2 | 3 | 3 | 100 |
| Ghana | 3 | 1 | 3 | 3 | 100 |
| Greece | 4 | 3 | 4 | 4 | 100 |
| Grenada | 3 | 1 | 3 | 3 | 100 |
| Guadeloupe |  | 1 |  | 1 | 33 |
| Guam |  | 1 |  | 1 | 33 |
| Guatemala | 2 | 1 | 4 | 3 | 100 |
| Guinea | 3 | 1 | 4 | 3 | 100 |
| Guinea-Bissau | 3 | 1 | 4 | 3 | 100 |
| Guyana | 2 | 1 | 3 | 2 | 100 |
| Haiti | 3 | 1 | 4 | 3 | 100 |
| Honduras | 3 | 1 | 4 | 3 | 100 |
| Hong Kong |  |  | 1 | 1 | 33 |
| Hungary |  | 1 | 2 | 2 | 67 |
| Iceland | 3 | 2 | 3 | 3 | 100 |
| India | 3 | 2 | 4 | 3 | 100 |
| Indonesia | 3 | 2 | 3 | 3 | 100 |
| Iran | 3 | 1 | 4 | 3 | 100 |
| Iraq | 4 | 1 | 4 | 3 | 100 |
| Ireland | 4 | 4 | 2 | 4 | 100 |
| Israel | 2 | 1 | 2 | 2 | 100 |
| Italy | 4 | 4 | 4 | 4 | 100 |
| Ivory coast | 3 | 1 | 4 | 3 | 100 |
| Jamaica | 2 | 2 | 3 | 3 | 100 |
| Japan | 5 | 4 | 3 | 4 | 100 |
| Jersey |  |  | 2 | 2 | 33 |
| Jordan |  | 1 | 3 | 2 | 67 |
| Kazakhstan |  | 1 | 3 | 2 | 67 |
| Kenya | 2 | 1 | 4 | 3 | 100 |
| Kiribati | 4 | 1 | 3 | 3 | 100 |
| Kosovo |  |  | 3 | 3 | 33 |
| Kuwait | 3 | 2 | 3 | 3 | 100 |
| Kyrgyzstan |  | 1 | 4 | 3 | 67 |
| Lao |  | 1 | 4 | 3 | 67 |
| Latvia |  | 2 | 2 | 2 | 67 |
| Lebanon | 2 | 2 | 4 | 3 | 100 |
| Lesotho |  | 1 | 3 | 2 | 67 |
| Liberia | 3 | 1 | 4 | 3 | 100 |
| Libya | 4 | 1 | 5 | 4 | 100 |
| Liechtenstein |  |  | 1 | 1 | 33 |
| Lithuania |  | 1 | 2 | 2 | 67 |
| Luxembourg |  | 3 | 1 | 2 | 67 |
| Macao |  |  | 2 | 2 | 33 |
| Macedonia |  | 1 | 3 | 2 | 67 |
| Madagascar | 3 | 1 | 4 | 3 | 100 |
| Malawi |  | 1 | 3 | 2 | 67 |
| Malaysia | 2 | 3 | 4 | 3 | 100 |
| Maldives | 3 | 1 | 3 | 3 | 100 |
| Mali |  | 1 | 4 | 3 | 67 |
| Malta | 4 | 3 | 2 | 3 | 100 |
| Marshall Islands | 4 | 1 | 3 | 3 | 100 |
| Martinique |  | 1 |  | 1 | 33 |
| Mauritania | 3 | 1 | 4 | 3 | 100 |
| Mauritius |  | 2 | 4 | 3 | 67 |
| Mayotte |  | 1 |  | 1 | 33 |
| Mexico | 3 | 2 | 3 | 3 | 100 |
| Micronesia | 4 |  | 3 | 4 | 67 |
| Moldova |  | 1 | 3 | 2 | 67 |
| Mongolia |  | 1 | 3 | 2 | 67 |
| Montenegro | 4 | 3 | 4 | 4 | 100 |
| Morocco | 3 | 1 | 3 | 3 | 100 |
| Mozambique | 3 | 1 | 4 | 3 | 100 |
| Myanmar |  | 1 | 4 | 3 | 67 |
| Namibia | 4 | 3 | 4 | 4 | 100 |
| Nauru | 4 | 1 | 3 | 3 | 100 |
| Nepal |  | 1 | 4 | 3 | 67 |
| Netherlands | 3 | 3 | 3 | 3 | 100 |
| New Caledonia |  | 3 | 5 | 4 | 67 |
| New Zealand | 3 | 4 | 3 | 4 | 100 |
| Nicaragua | 2 | 2 | 4 | 3 | 100 |
| Niger |  | 1 | 4 | 3 | 67 |
| Nigeria | 3 | 1 | 4 | 3 | 100 |
| Northern Mariana Is. |  | 1 |  | 1 | 33 |
| North Korea | 4 | 3 | 5 | 4 | 100 |
| Norway | 3 | 2 | 3 | 3 | 100 |
| Oman | 3 | 1 | 3 | 3 | 100 |
| Pakistan | 2 | 1 | 4 | 3 | 100 |
| Palau | 4 | 4 | 3 | 4 | 100 |
| Palestine |  | 1 | 3 | 2 | 67 |
| Panama | 2 | 2 | 3 | 3 | 100 |
| Papua New Guinea | 4 | 1 | 4 | 3 | 100 |
| Paraguay |  | 1 | 3 | 2 | 67 |
| Peru | 3 | 5 | 4 | 4 | 100 |
| Philippines | 4 | 3 | 4 | 4 | 100 |
| Poland | 4 | 1 | 2 | 3 | 100 |
| Portugal | 4 | 4 | 2 | 4 | 100 |
| Puerto Rico | 4 | 1 | 2 | 3 | 100 |
| Qatar | 2 | 1 | 2 | 2 | 100 |
| Réunion |  | 1 |  | 1 | 33 |
| Romania | 4 | 2 | 4 | 4 | 100 |
| Russia | 4 | 2 | 4 | 4 | 100 |
| Rwanda |  | 1 | 3 | 2 | 67 |
| Saint Kitts and Nevis | 3 | 2 | 3 | 3 | 100 |
| Saint Lucia | 3 | 1 | 2 | 2 | 100 |
| St. Vincent and the Grenadines | 3 | 2 | 2 | 3 | 100 |
| Samoa | 3 | 2 | 2 | 3 | 100 |
| Sao Tome and Principe | 3 | 1 | 3 | 3 | 100 |
| Saudi Arabia | 3 | 1 | 3 | 3 | 100 |
| Senegal | 3 | 3 | 3 | 3 | 100 |
| Serbia |  | 1 | 3 | 2 | 67 |
| Seychelles | 2 |  | 3 | 3 | 67 |
| Sierra Leone | 3 | 2 | 4 | 3 | 100 |
| Singapore |  | 3 | 3 | 3 | 67 |
| Slovakia |  | 2 | 2 | 2 | 67 |
| Slovenia |  | 3 | 4 | 4 | 67 |
| Solomon Islands | 4 | 2 | 3 | 3 | 100 |
| Somalia |  |  | 5 | 5 | 33 |
| South Africa | 2 | 3 | 3 | 3 | 100 |
| South Korea | 4 | 4 | 3 | 4 | 100 |
| South Sudan |  | 1 | 5 | 3 | 67 |
| Spain | 3 | 4 | 4 | 4 | 100 |
| Sri Lanka | 3 | 2 | 3 | 3 | 100 |
| Saint Pierre and Miquelon |  | 3 | 5 | 4 | 67 |
| Sudan | 3 | 1 | 5 | 3 | 100 |
| Suriname | 2 | 1 | 3 | 2 | 100 |
| Swaziland |  | 1 | 4 | 3 | 67 |
| Sweden | 3 | 2 | 3 | 3 | 100 |
| Switzerland |  | 2 | 1 | 2 | 67 |
| Syria | 4 | 1 | 5 | 4 | 100 |
| Taiwan | 5 | 3 | 2 | 4 | 100 |
| Tajikistan |  | 1 | 4 | 3 | 67 |
| Tanzania | 2 | 1 | 3 | 2 | 100 |
| Thailand | 3 | 4 | 3 | 4 | 100 |
| Timor-Leste |  | 1 | 4 | 3 | 67 |
| Togo | 3 | 1 | 4 | 3 | 100 |
| Tonga | 4 | 3 | 4 | 4 | 100 |
| Trinidad and Tobago | 2 | 2 | 3 | 3 | 100 |
| Tunisia | 5 | 1 | 3 | 3 | 100 |
| Turkey | 5 | 2 | 3 | 4 | 100 |
| Turkmenistan |  | 1 | 4 | 3 | 67 |
| Tuvalu | 3 | 1 | 3 | 3 | 100 |
| Uganda |  | 1 | 4 | 3 | 67 |
| Ukraine | 4 | 3 | 5 | 4 | 100 |
| United Arab Emirates | 4 | 2 | 2 | 3 | 100 |
| United Kingdom | 3 | 3 | 3 | 3 | 100 |
| United States | 4 | 3 | 2 | 3 | 100 |
| Uruguay | 3 | 2 | 2 | 3 | 100 |
| US Virgin Islands |  | 1 | 4 | 3 | 67 |
| Uzbekistan |  | 1 | 3 | 2 | 67 |
| Vanuatu | 4 | 2 | 5 | 4 | 100 |
| Venezuela | 3 | 3 | 4 | 4 | 100 |
| Vietnam | 5 | 3 |  | 4 | 67 |
| West Bank and Gaza |  | 1 | 4 | 3 | 67 |
| Yemen | 2 | 1 | 5 | 3 | 100 |
| Zambia |  | 1 | 4 | 3 | 67 |
| Zimbabwe |  | 1 | 4 | 3 | 67 |

Allison, E. H., A. L. Perry, M.-C. Badjeck, W. Neil Adger, K. Brown, D. Conway, A. S. Halls, G. M. Pilling, J. D. Reynolds, N. L. Andrew and N. K. Dulvy (2009). "Vulnerability of national economies to the impacts of climate change on fisheries." Fish and Fisheries **10**(2): 173-196.

Allison, E. H., A. L. Perry, M. C. Badjeck, W. Neil Adger, K. Brown, D. Conway, A. S. Halls, G. M. Pilling, J. D. Reynolds and N. L. Andrew (2009). "Vulnerability of national economies to the impacts of climate change on fisheries." Fish and fisheries **10**(2): 173-196.

CCCMA. (2016, 2005). "Canadian Centre for Climate Modelling and Analysis." 2016, from <http://www.cccma.bc.ec.gc.ca/data/data.shtml>.

Collins, W., N. Bellouin, M. Doutriaux-Boucher, N. Gedney, T. Hinton, C. Jones, S. Liddicoat, G. Martin, F. O’Connor and J. Rae (2008). "Evaluation of the HadGEM2 model." Hadley Cent. Tech. Note **74**.

Cooley, S. R., H. L. Kite-Powell and S. C. Doney (2009). "Ocean acidification’s potential to alter global marine ecosystem services."

Cooley, S. R., N. Lucey, H. Kite-Powell and S. C. Doney (2012). "Nutrition and income from molluscs today imply vulnerability to ocean acidification tomorrow." Fish and Fisheries **13**(2): 182-215.

FAO (2012). FAOSTAT. Rome, Italy, FAO.

FAO. (2016). " Fishery and Aquaculture Statistics." from <http://www.fao.org/fishery/statistics/global-aquaculture-production/en>.

Grassle, J. F. (2000). "The Ocean Biogeographic Information System (OBIS): an on-line, worldwide atlas for accessing, modeling and mapping marine biological data in a multidimensional geographic context." Oceanography **13**(3): 5-7.

Handisyde, N., L. Ross, M. Badjeck and E. Allison (2006). "The effects of climate change on world aquaculture: a global perspective." Aquaculture and Fish Genetics Research Programme, Stirling Institute of Aquaculture. Final Technical Report, DFID, Stirling. 151pp.

Henson, S. A., C. Beaulieu and R. Lampitt (2016). "Observing climate change trends in ocean biogeochemistry: when and where." Global change biology **22**(4): 1561-1571.

Kaufmann, D., A. Kraay and M. Mastruzzi (2011). "The worldwide governance indicators: methodology and analytical issues." Hague Journal on the Rule of Law **3**(02): 220-246.

Moss, R. H., J. A. Edmonds, K. A. Hibbard, M. R. Manning, S. K. Rose, D. P. Van Vuuren, T. R. Carter, S. Emori, M. Kainuma and T. Kram (2010). "The next generation of scenarios for climate change research and assessment." Nature **463**(7282): 747-756.

Shannon, C. E. and W. Weaver (1949). "The mathematical theory of information."
